# Profiling maize embryonic leaf development and discovering new genes using high-resolution spatial long-read isoform sequencing

**DOI:** 10.1038/s41477-026-02364-y

**Published:** 2026-08-17

**Authors:** Wye-Lup Kong, Chi-Chih Wu, Yi-Hua Chen, Jeng-Yi Li, Kai Xuan Tin, Yi-Chen Lee, Zhi Thong Soh, Chun-Shiu Wu, Yao-Ming Chang, Wen-Hsiung Li, Mei-Yeh Jade Lu

**Affiliations:** 1 Biodiversity Research Center, Academia Sinica, Taipei, Taiwan; 2 Institute of Plant and Microbial Biology, Academia Sinica, Taipei, Taiwan; 3 Department of Agronomy, National Taiwan University, Taipei, Taiwan; 4 Institute of Biomedical Science, Academia Sinica, Taipei, Taiwan; 5 Department of Ecology and Evolution, University of Chicago, Chicago, IL USA

**Keywords:** Plant embryogenesis, RNA sequencing

## Abstract

Profiling transcriptome isoforms in their spatial context is instrumental for deciphering plant embryogenesis. By combining high-throughput full-length isoform sequencing and spatial transcriptomics (spatial MAS-IsoSeq) in maize embryogenesis, we identified 285,639 isoforms, 72.87% of which were previously uncharacterized. Gene models based on these full-length isoforms increased short-read exon mapping by 5.52%. Furthermore, spatial transcription expression detection improved by up to 97.45% in an extreme example. Using these isoforms, we constructed a new gene-model database (MaizeV5_IsoAnn) by integrating 5,228 novel genes and 1,674 genes with 5′- and/or 3′-flanking region extensions into the current maize reference gene models. Leveraging MaizeV5_IsoAnn, we reanalysed embryonic leaf cell transcriptomes to construct a refined time-ordered regulatory network and integrated it into multi-omics analyses with chromatin accessibility dynamics profiling, providing new insights into maize embryonic leaf development. Moreover, we propose *LBD26* as an essential transcription factor in maize embryonic vein development. This study underscores the power of spatial MAS-IsoSeq to construct gene-model databases and elucidate developmental processes and mechanisms.

## Abstract (web summary)

Kong et al. integrate replicated spatial long reads, epigenomics and in situ validation to build MaizeV5_IsoAnn, a rich resource cataloguing 5,228 novel genes and functional isoforms to resolve regulatory networks for the plant community.

## Main

This study aims to show the power of combining spatial transcriptomics and high-throughput full-length isoform sequencing (spatial MAS-IsoSeq) in revealing plant developmental processes and mechanisms. Understanding gene expression in its spatial context is instrumental for deciphering complex developmental processes such as plant embryogenesis. Indeed, spatial transcriptomics technologies offer the advantage of mapping gene expression patterns directly in intact tissue sections, providing insights into developmental transitions in plants such as maize ear primordium^1^ and wheat inflorescence^2^. However, these applications are limited to gene-level expression profiles, overlooking the critical biological information conveyed by transcript isoforms. Transcript isoforms, arising from alternative splicing and alternative transcription start/termination sites, markedly contribute to transcriptome complexity by encoding distinct proteins tailored to specific developmental stages and tissue types^3–7^. Current reference gene-model databases, often compiled from specific tissues or life stages, frequently lack comprehensive isoform annotations, particularly for less-characterized tissues and dynamic developmental conditions^8,9^.

The advent of long-read sequencing technologies, by Pacific Biosciences (PacBio)^10^ and Oxford Nanopore Technologies^11^, offers a solution to this isoform-level challenge. PacBio’s Iso-Seq method generates ultra-long reads that span entire transcripts, enabling the direct identification of full-length isoforms with high accuracy, an important advantage over short-read RNA-seq, which requires error-prone computational reconstruction^12^. The recent Multiplexed Array Isoform Sequencing (MAS-IsoSeq)^13^ further enhances this capability by dramatically increasing throughput by five- to eightfold. This was achieved by concatenating 16 full-length complementary DNA segments into a single long molecule for HiFi sequencing. This overcomes traditional Iso-Seq’s throughput limitation, which has been a major factor driving researchers to alternative long-read sequencing platforms such as Oxford Nanopore Technologies^14^. While these long-read technologies excel at isoform discovery, they traditionally lack spatial information. Conversely, existing spatial transcriptomics approaches in plants^1,2,15–18^, while providing spatial context, are limited by short-read detection or constrained gene probes, thus failing to capture the full landscape of isoform expression. Other cellular resolution techniques such as laser capture microdissection (LCM)^19^ are laborious and lack spatial continuity, and single-cell RNA sequencing^20^ lacks spatial information, relying on marker genes for cell type identification.

**Fig. 1:**
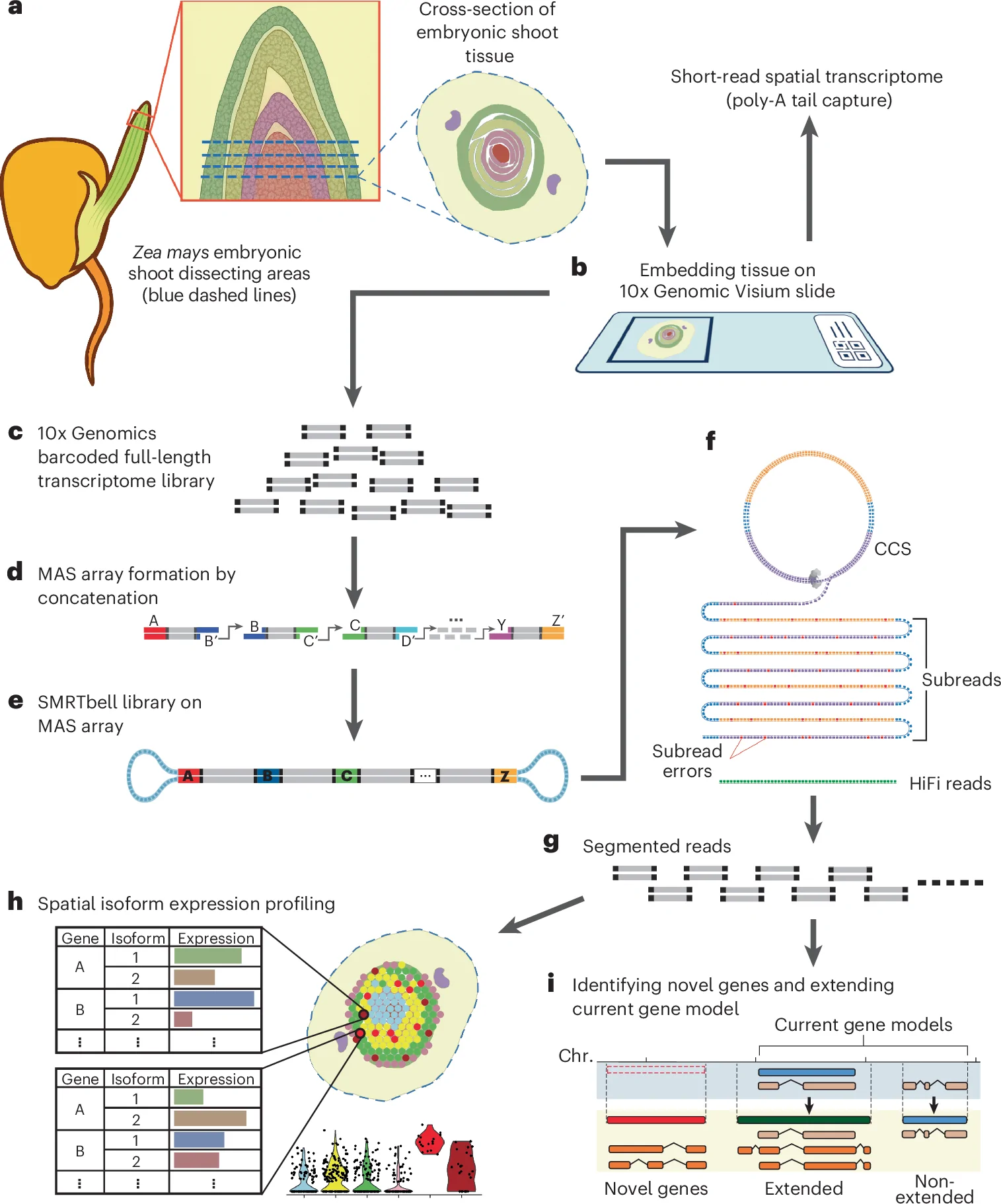
Schematic workflow of spatial MAS-IsoSeq. **a**, Embryonic shoots of *Z. mays* seedlings are horizontally sliced into four serial sections and mounted on a 10x Genomics Visium Gene Expression (GE) slide. **b**, Full-length RNA transcripts are released onto the Visium GE slide surface and captured by oligo-dT primers containing spatial barcodes and UMIs. **c**, This process produces a 10x Genomics barcoded full-length cDNA library, and a portion of this library can be used for short-read transcriptomics sequencing. **d**, The transcripts are then concatenated into multiplexed linear arrays, each containing up to 16 full-length cDNA molecules. **e**, The multiplexed arrays are annealed to SMRTbell hairpin adapters at both ends, forming the Multiplexed Array Sequencing (MAS) library. **f**, During PacBio circular consensus sequencing (CCS), each cDNA undergoes multiple passes to generate consensus subreads, resulting in highly accurate HiFi reads (*Q* > 40). HiFi illustration adapted with permission from PacBio. **g**, The HiFi arrayed reads are then deconcatenated to obtain individual full-length cDNA S-reads with 10x spatial barcodes and UMIs. **h**, Isoform expression of each spatial spot was profiled, enabling spatial spot clustering at higher resolution. **i**, Isoform-assisted identification of novel genes and end extensions of 5′ and/or 3′ exons using the full-length transcriptome data. These gene models are integrated into the currently available database to construct an isoform-annotated gene-model database, named MaizeV5_IsoAnn. The schematic illustrations of the MAS workflow (**c**–**e**) are adapted with permission from PacBio.

To overcome these limitations, we propose the integrated spatial MAS-IsoSeq approach, which provides accurate spatial profiling of isoform expression, enabling the identification of novel genes and extending current gene models (Fig. 1). To demonstrate the power and utility of spatial MAS-IsoSeq, we apply it to investigate embryonic leaf development in maize (*Zea mays*), including the formation of its specialized Kranz anatomy^21,22^. A key objective is the construction of MaizeV5_IsoAnn, a comprehensive isoform-annotated gene-model database built on B73 RefGen_v5, the current maize gene-model database, by leveraging the high-quality full-length isoforms generated in this study along with advanced bioinformatics tools. We explored the putative functions of the isoforms by investigating their protein-coding potential and protein domain retention/loss, identifying tentative conserved noncoding sequences, and examined the chromatin accessibility tentatively associated with the isoforms, providing integrated multi-omics aspects of the spatial isoforms.

Furthermore, to illustrate the practical utility and analytical power of this new resource, we reanalyse 30 published transcriptomic datasets from LCM-isolated maize embryonic leaf cells^19^. This reanalysis, enabled by MaizeV5_IsoAnn, aims to refine existing regulatory networks and identify key developmental regulators through detailed computational analyses, leading to our finding of LBD26 as a vein development transcriptional regulator, which is subsequently validated in the promoter binding analysis. This study is designed to establish spatial MAS-IsoSeq as a powerful tool for dissecting complex plant tissue development and, critically, to provide a robust and comprehensive gene-model resource to shed novel biological insights into plant development.

## Results

### Spatial full-length transcriptome of the maize embryonic leaf

We generated a comprehensive full-length spatial transcriptome dataset from maize embryonic leaf sections using PacBio MAS-IsoSeq. From three MAS-IsoSeq runs of the same spatial transcriptome library, we obtained a total of 92,530,097 HiFi full-length transcriptome segmented reads (S-reads), with a mean read length of 12,627 bp (Supplementary Table 1). Each long-read transcript in this dataset contained a unique molecular identifier (UMI) coupled with a spatial barcode, enabling precise localization on the Visium tissue map.

**Fig. 2:**
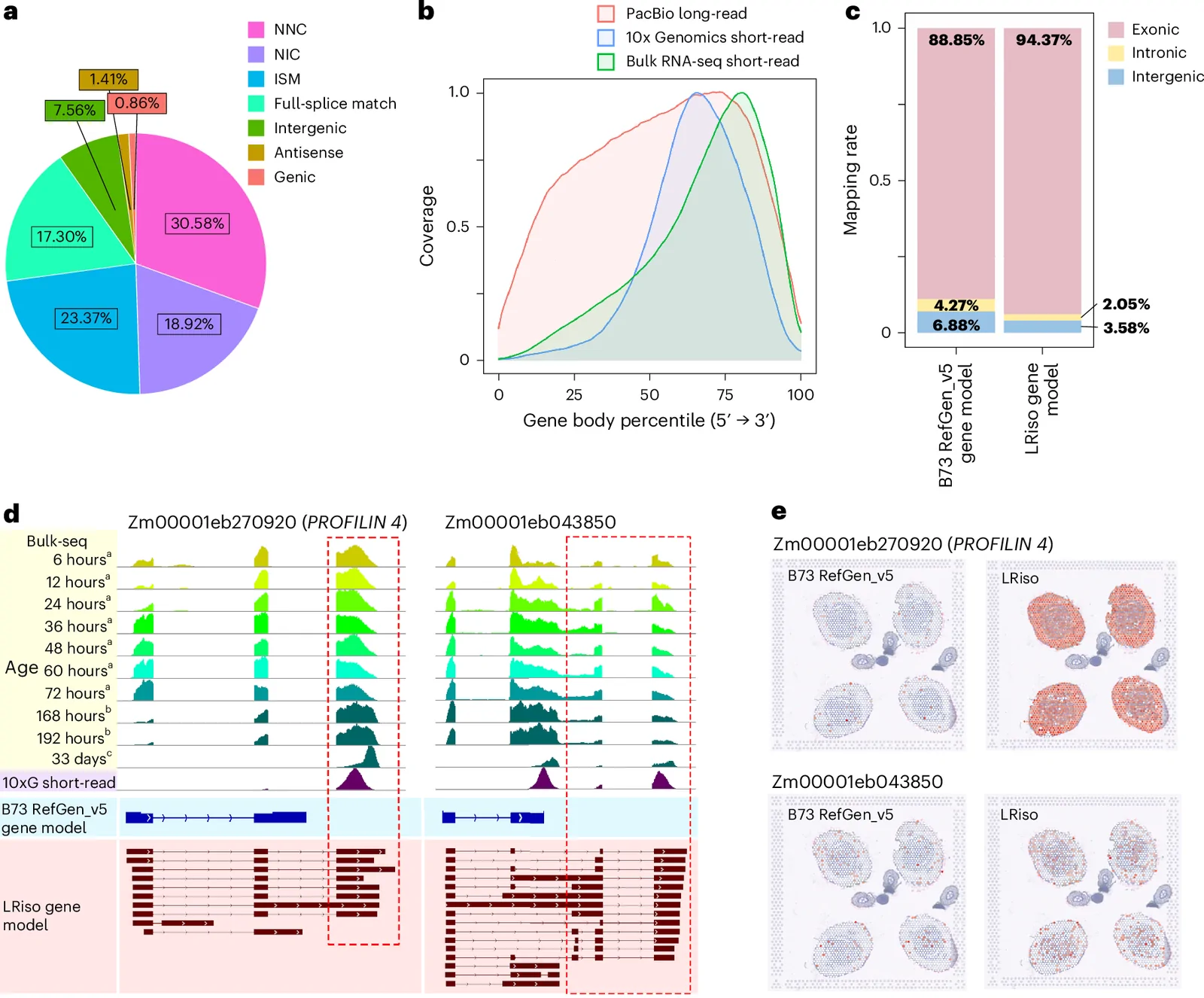
Characterization of full-length isoform transcripts from maize embryonic leaf tissues. **a**, Classification of isoform structure categories into a pie chart. The novel isoforms included in the categories NIC, NNC and ISM account for 72.87% of the total isoforms detected. **b**, Comparison of 5′ end coverage: PacBio long-read (red) mapping shows much higher coverage at the 5′ end of the gene body than both 10x Genomics (blue) and bulk RNA-seq (green) short-read mapping. **c**, Short-read exonic mapping shows a noticeably higher rate onto the LRiso gene models than onto the *Z. mays* B73 RefGen_v5 gene models (94.37% versus 88.85%). **d**, Mapping coverages of bulk RNA-seq and 10x Genomics Visium short reads for two exemplary genes, Zm00001eb270920 (*PROFILIN 4*) and Zm00001eb043850. Both genes have isoforms with novel 3′-end exons in the LRiso gene model. A large portion of both bulk-seq and 10x spatial reads map to this novel region (in red rectangles). The bulk-seq data originated from LCM samples of maize leaf tissue at different developmental stages (post-imbibition time-course). The alphabet superscripts indicate the published data sources (superscripts ‘a’^25^, ‘b’^26^ and ‘c’^27^). **e**, Spatial gene expression profiles of the Zm00001eb270920 (top) and Zm00001eb043850 (bottom) genes on Visium spatial spots under two different reference gene models. In the LRiso model (right), both genes show notably higher expression levels in the barcoded spatial spots (the red spots) than in the B73 RefGen_v5 gene model (left). Only leaf tissues were included in this analysis (excluding the root tissue).

These reads were collapsed and mapped to the *Z. mays* B73 version 5.0 reference genome (GCF_902167145.1, accessed in October 2023) to construct a long-read isoform-based (LRiso) gene-model database using the standard PacBio Pigeon analysis pipeline. Reads not aligned to the reference genome, which account for a minor fraction (0.89%, 344,945 of 38,888,149 total reads), were excluded from further analysis. A total of 38,543,204 reads were uniquely mapped to their corresponding genomic coordinates. The LRiso database comprises 35,001 genes and 285,639 distinct isoform transcripts. Of these genes, 27,966 correspond to known loci documented in the B73 RefGen_v5 reference, whereas the remaining 7,035 represent previously unrecorded genes located in intergenic regions. Each full-length isoform in the LRiso gene models was systematically classified against B73 RefGen_v5 (Fig. 2a). Strikingly, 72.87% of the identified isoforms were previously unrecorded in the latest maize gene-model database (MaizeGDB)^23^. These isoforms, which fall into the structural categories of ‘incomplete-splice match’ (ISM), ‘novel in catalog’ (NIC) and ‘novel not in catalog’ (NNC), are hereafter collectively defined as ‘novel isoforms’ throughout this study (Supplementary Table 2). When we compared gene body coverage between long-read and short-read datasets, long reads exhibited considerably higher gene body coverage at both the 5′ and 3′ ends of transcripts. We observed this when comparing with conventional 10x Genomics short-read sequencing from this study and 81 combined LCM bulk-RNAseq short-read datasets retrieved from Liu et al.^19^ (Fig. 2b). This enhanced coverage increases the likelihood of identifying isoforms with alternative transcription start sites (TSSs) near the 5′ end and alternative transcription termination sites at the 3′ end^14^.

Leveraging the LRiso database as the reference for mapping 10x Genomics short-read datasets revealed improvements in gene mapping sensitivity. Compared with mapping against B73 RefGen_v5, the exon mapping rate for short reads increased from 88.85% to 94.37% (Fig. 2c). The enhanced sensitivity was accompanied by a reduction in reads mapped to intronic and intergenic regions by 2.43% and 3.30%, respectively (Fig. 2c). Among the 26,430 genes common to both gene models, 1,241 (4.70%) genes showed higher mapped read counts with LRiso. However, 1,498 (5.67%) genes exhibited reduced mapped read counts, while 23,691 (89.63%) showed no change. A closer examination indicated that decreases in mapped read counts in these genes were primarily due to novel isoforms from other genes overlapping with existing gene bodies in LRiso. This can cause reads mapping to overlapping regions to be ambiguously labelled and excluded from counting. This finding underscores the importance of careful annotation and filtering of overlapping gene models when building comprehensive transcriptomic resources to prevent potential underestimation of gene expression.

### Novel 3′ exons dramatically raise short-read mapping rates

To investigate the basis behind the superior mapping sensitivity, we inspected the short-read mapping results of 10x Genomic Visium 3′-captured libraries on the LRiso gene models. Using Integrative Genomics Viewer^24^, we identified genes containing novel isoforms with previously uncharacterized 3′ exons, which drastically increased the detection of reads mapping to these expanded genic regions. When we compared the 3′-end locations of gene models between the B73 RefGen_v5 and LRiso databases, 13,144 (49.73%) out of 26,430 gene models present in both databases had extended 3′ ends (Supplementary Table 3). Of these extended gene models, 5,230 showed an increase in mapped read count.

Here we provide two prominent examples, Zm00001eb270920 and Zm00001eb043850, which consist of isoforms possessing novel 3′ exons in the LRiso gene models (Fig. 2d). The inclusion of 10x Genomics short reads mapped to these novel 3′ exons in the LRiso gene models increased mapped read counts by 30-fold (from 1,014 to 30,993 mapped reads) for Zm0001eb270920 and by 2-fold (from 834 to 1,554 mapped reads) for Zm00001eb043850. The spatial detection rate of these two genes’ transcripts across all Visium spatial spots dramatically increased from 2.94% to 97.45% for Zm00001eb270920 and from 6.85% to 26.21% for Zm00001eb043850 (Fig. 2e).

To investigate whether such mapping rate enhancement is unique to certain stages in maize embryonic leaf development, we mapped ten bulk-seq datasets of various time points from post imbibition to mature leaf (6, 12, 24, 36, 48, 60, 72 (ref. ^25^), 168 and 192 hours^26^, and 33 days^27^). The mapping results show that the bulk-seq datasets of all development time points consistently contain a noticeable portion of short reads mapping to the regions with novel 3′ exons in the LRiso gene models (Fig. 2d).

### Functional and multi-omics assessment of novel isoforms

To validate the 208,148 inferred novel isoforms (comprising the ISM, NIC and NNC categories), we evaluated their protein-coding potential and identified conserved functional domains. We further characterized isoforms featuring alternative 5′ TSSs and novel 3′ exons by analysing the presence of proximal accessible chromatin regions (ACRs) and canonical polyadenylation (poly-A) motifs.

**Extended Data Fig. 1:**
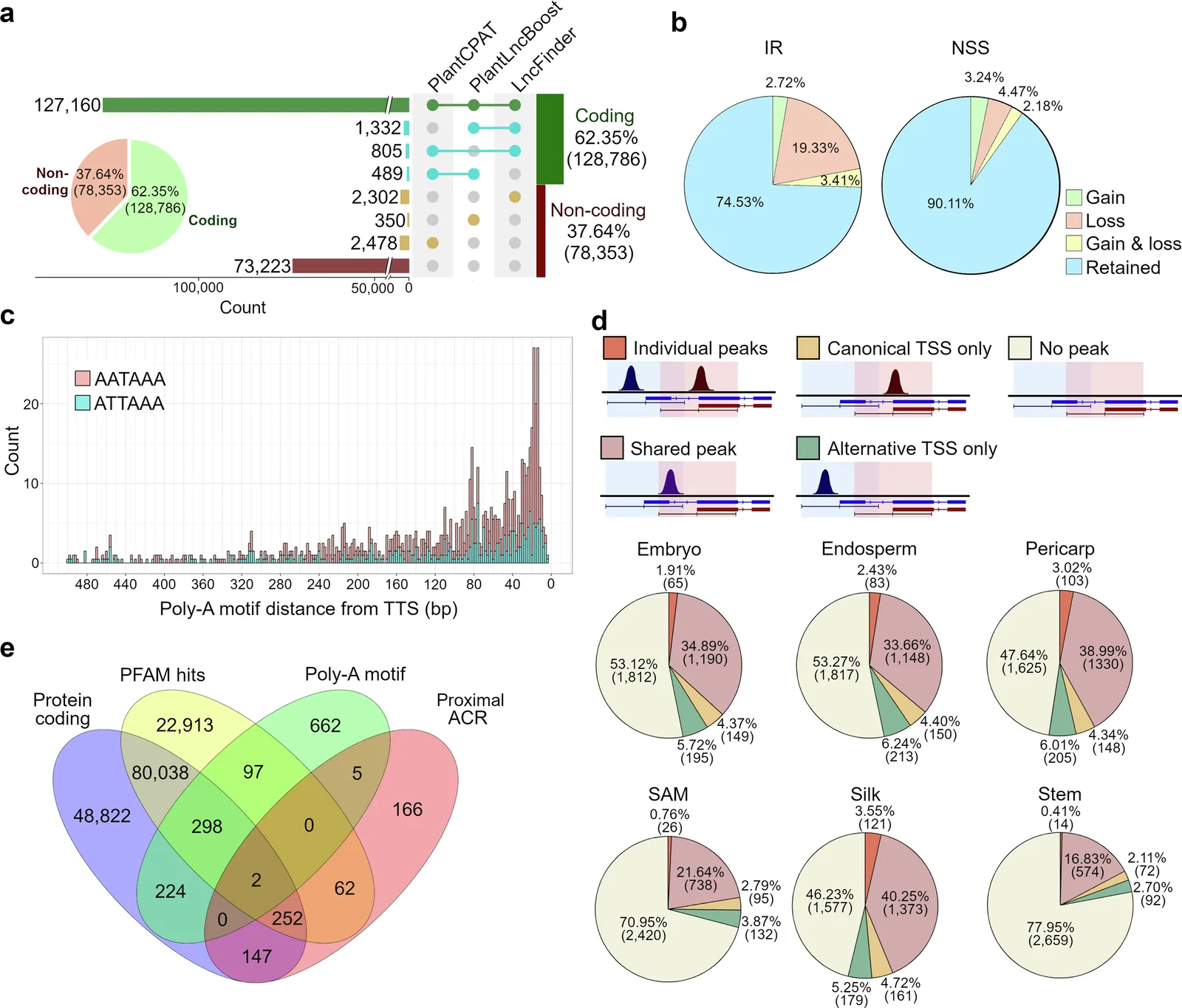
Multi-omics assessment and functional characterization of unannotated transcripts. **a**, Assessment of protein-coding probability for 127,160 novel isoforms—representing the subset of transcripts associated with known genes—using a consensus of three distinct bioinformatic algorithms. A total of 62.35% of these isoforms were predicted to be protein-coding based on the support by at least two tools. **b**, Functional impact of alternative splicing on PFAM domains within novel isoforms. The analysis compares isoforms affected by novel splice sites (NSS) versus those with intron retention (IR). While a large percentage of novel isoforms in both categories retained their PFAM domains, NSS isoforms exhibited higher retention rates (90.11%) compared to IR isoforms (74.53%). **c**, Positional distribution of polyadenylation (poly-A) motifs within novel 3’ exons. The *x*-axis indicates the distance (bp) upstream of the transcription termination site (TTS), revealing a distinct enrichment peak between 10 and 40 bp upstream of the TSS. **d**, Evaluation of accessible chromatin regions (ACRs, ±1000 bp of TSS) at canonical and alternative transcription start sites (TSSs) using the integrated bulk ATAC-seq datasets retrieved from Liu et al.^33^. Canonical TSS is defined specifically as the most upstream TSS among previously known isoforms, whereas alternative TSSs represent novel sites located at least 100 bp upstream of the canonical position. Schematic diagrams (top) categorize the different types of ATAC-seq peak support as ‘Individual’, ‘Shared’, ‘Canonical TSS only’, or ‘Alternative TSS only’. **e**, Venn diagram integrating four independent lines of evidence: protein-coding potential, PFAM domain hits, poly-A motif presence, and proximal ACR support. The intersection highlights that the majority of unannotated isoforms—80,590 out of the153,688 isoforms possessing at least one supporting feature—are corroborated by both protein-coding potential and PFAM domain hits.

Using three independent algorithms—Plant-CPAT^28^, PlantLncBoost^29^ and LncFinder^30^—we assessed the coding potential of each novel isoform. Our analysis revealed that 62.35% (128,786) of the 208,148 inferred novel isoforms were classified as protein-coding (Extended Data Fig. 1a). Among these, 127,160 (61.09%) isoforms were consistently predicted as coding by all three tools, representing a high-confidence set of novel coding transcripts. Conversely, only 73,223 isoforms were identified as noncoding by all three algorithms.

To determine the functional consequences of alternative splicing, we scanned all novel isoforms for protein domains using PFAMscan^31^. We identified functional domains in 49.80% (104,482/208,148) of the novel isoforms (Extended Data Fig. 1b). Novel isoforms characterized by intron retention (IR) showed a 19.33% loss of PFAM domains compared with their canonical counterparts. In contrast, 90.11% of isoforms containing at least one novel splice site retained the original PFAM domains, a notably higher retention rate than the 74.43% observed for IR isoforms (Extended Data Fig. 1b).

For the 5,445 isoforms featuring novel 3′ exons, we searched for the canonical poly-A motif (AATAAA) and its most common maize variant (ATTAAA)^32^. We found that 23.65% (1,288/5,445) of these novel 3′ exons harboured at least one poly-A motif (Extended Data Fig. 1c). The distribution of these motifs showed a distinct enrichment peak between 10 and 40 bp upstream of the transcription termination site, consistent with established poly-A patterns in *Z. mays*^32^. Further analysis of the open reading frames (ORFs) revealed 190 novel 3′ exons that represent coding sequence fragments associated with extended 3′ untranslated regions (UTRs).

To investigate the transcriptional regulation of alternative TSSs, we integrated public ATAC-seq datasets (PRJNA1048297)^33^ across six maize tissues to identify and compare ACRs within promoter regions (±1,000 bp of the TSS) of canonical and alternative TSSs (Extended Data Fig. 1d). In this analysis, the canonical TSS is defined specifically as the most upstream TSS among previously known isoforms, whereas alternative TSSs represent novel sites located at least 100 bp upstream of the canonical position. We categorized ATAC-seq peaks into four types: ‘Individual’, featuring separate, distinct ATAC-seq peaks at the promoter regions of both the canonical and alternative TSSs; ‘Shared’, where a single broad peak spans the overlapping promoter regions; ‘Canonical TSS-only’, where peaks are exclusive to canonical TSS; and ‘Alternative TSS-only’, where peaks are detected solely at the alternative TSS. Peaks in the ‘Individual’ and ‘Alternative TSS-only’ categories indicate potential regulatory context for novel transcription initiation by confirming chromatin accessibility specifically at the alternative site. Among 3,411 genes with alternative TSSs, 7.62% (260/3,411) possessed ATAC peaks unique to the alternative TSS in embryo tissue (Extended Data Fig. 1d). This trend was consistent across endosperm (8.67%), pericarp (9.03%) and silk (8.80%), with slightly lower rates in the shoot apical meristem (SAM) (4.63%) and stem (3.11%).

Finally, we integrated these four independent lines of evidence—coding potential, PFAM domains, poly-A motifs and ATAC-seq peaks—into a unified computational assessment framework (Supplementary Table 4). Overall, 73.84% (153,688/208,148) of novel isoforms were corroborated by at least one experimental or computational feature. Most notably, 38.72% (80,590) of novel isoforms were supported by both protein-coding potential and PFAM domain hits (Extended Data Fig. 1e).

### Isoform clustering reveals leaf and vein developmental stages

**Fig. 3:**
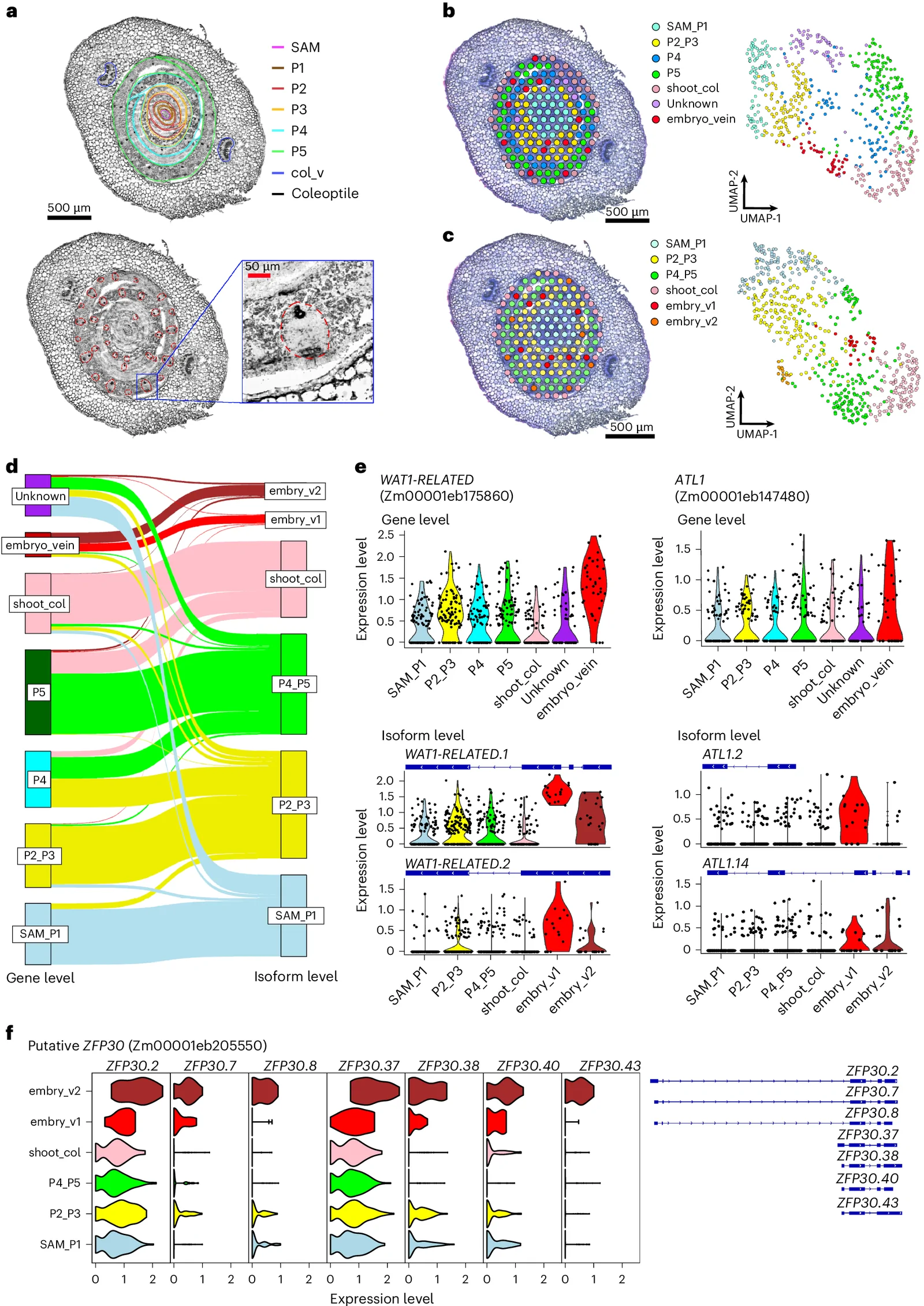
Unsupervised clustering of spatial transcriptomic spots. The clustering reveals differential expression between the SAM, the differentiated states of primordia leaves (P1–P5) and the embryonic veins. **a**, Histology image of a maize shoot cross-section. This is the representative tissue section from a total of 16 independent experiments at similar positions along the longitudinal axis of a previous study^41^. Top: histology image showing the tissue domains labelled according to the morphological observation in the cross-section. Bottom: red dashed circles in the histology image highlighting the location of the embryonic vein tissue. **b**,**c**, Unsupervised clustering of leaf tissue spots at the gene (**b**) and isoform (**c**) levels. The clustered tissue domain annotations are represented by different colours in the UMAP (left), and the spots are overlaid onto the tissue map (right), revealing separation of the veins into two developmental stages (embry_v1 and embry_v2) at the isoform level. **d**, Sankey plot showing the pairing of spatial spots between gene-level and isoform-level unsupervised clustering. Each line represents a single spatial spot. **e**, Violin plots depicting the gene- and isoform-level expression levels of the *WAT1-RELATED* (left) and *ATL1* (right) genes in different tissue clusters. **f**, Violin plot (left) showing the expression levels of seven highly expressed isoforms of the putative *ZFP30* gene (Zm00001eb205550). The *ZFP30* isoforms under the LRiso gene model are shown on the right.

Histological images of four cross-sections from the maize embryonic shoot clearly delineate distinct tissue domains (Fig. 3a). The SAM is centrally located, enveloped by five layers of primordia leaves (P1 to P5). The coleoptile is located at the outermost section, consisting of two kidney-shaped coleoptile vein tissues (col_v).

We first examined whether the overall expression profiles of the embryonic shoot tissue were consistent across different library construction methods and sequencing platforms. Uniform Manifold Approximation and Projection (UMAP) plots generated from short-read gene-level data (Supplementary Fig. 1a), long-read gene-level data (Supplementary Fig. 1b) and long-read isoform-level data (Supplementary Fig. 1c) all exhibited similar topological patterns, with distinct and similarly arranged ‘leaves’, ‘coleoptile’ and ‘coleoptile vein’ regions. This indicated small differences in the overall expression profiles between these datasets.

We then performed unsupervised clustering of spatial spots on the UMAP projections to identify distinct tissue domains. Coleoptile spots were excluded to achieve higher resolution for embryonic leaves and veins (Supplementary Fig. 1a–c). Unsupervised clustering revealed seven distinct clusters at the gene level (Fig. 3b) and six distinct clusters at the isoform level from the long-read datasets (Fig. 3c and Supplementary Table 5). Overlaying these clusters onto the histology images allowed for their annotation into specific tissue domains: SAM_P1, P2_P3, P4_P5 and shoot_col (the border layer between primordial leaf and coleoptile tissues) for both gene- and isoform-level analyses (Supplementary Table 5). Spatial spots from three clusters—one from gene-level analysis (red cluster in Fig. 3b) and two from isoform-level analysis (red and orange clusters in Fig. 3c)—aligned precisely with the embryonic vein tissue domain, identifiable by dark blue staining on the histology image (Fig. 3a). These were named ‘embryo_vein’ (gene-level), and ‘embry_v1’ and ‘embry_v2’ (isoform-level), respectively. One ‘unknown’ cluster (light purple in Fig. 3b) from gene-level analysis could not be precisely annotated on the basis of marker genes or histology (Supplementary Table 5).

In finding the corresponding spatial spots between gene-level and isoform-level unsupervised clustering, a Sankey plot (Fig. 3d) revealed that the gene-level embryo_vein cluster was distinctly resolved into two clusters, embry_v1 and embry_v2, at the isoform level. Further examination of marker genes and isoforms elucidated this distinction (Supplementary Fig. 2 and Supplementary Tables 6 and 7). For instance, the *WALL ARE THIN1-RELATED* (*WAT1-RELATED*) and *AUXIN TRANSPORTER-LIKE1* (*ATL1*) genes contain isoforms (*WAT1-RELATED.1*, *WAT1-RELATED.2*, *ATL1.2* and *ATL1.14*) with distinct expression patterns between embry_v1 and embry_v2 (Fig. 3e and Supplementary Fig. 3a,b).

**Extended Data Fig. 2:**
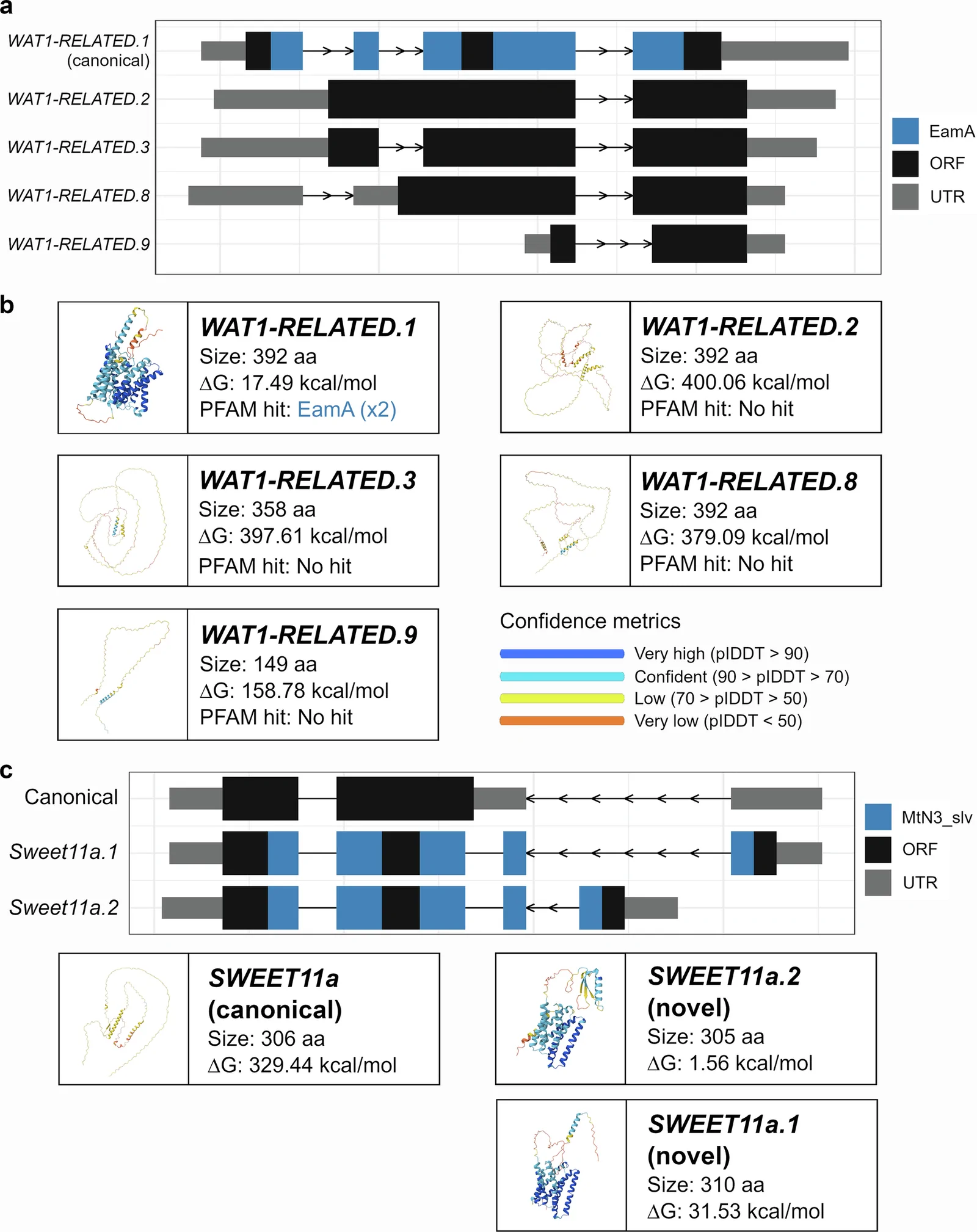
Functional impacts of alternative splicing on transcript and predicted protein isoforms of *WAT1-RELATED* and *SWEET11a*. **a**, Schematic representation of the open reading frame (ORF) and the untranslated (UTR) region for five *WAT1-RELATED* isoforms. The canonical isoform (*WAT1-RELATED.1*) contains two conserved EamA domains (blue), which are absent in the four novel isoforms due to alternative splicing events. **b**, Predicted protein structures for the five *WAT1-RELATED* isoforms, including their amino acid (aa) length, predicted folding free energy (*∆G*), and PFAM domain hits. Structural models are color-coded by the predicted local distance difference test (pLDDT) confidence scores, ranging from ‘Very high’ (blue, >90) to ‘Very low’ (orange, <50). **c**, Comparison of gene models and predicted protein structures between the canonical reference and novel isoforms of the *SWEET11a* gene. The canonical model is based on the MaizeGDB reference (Zm-B73-REFERENCE-NAM-5.0). Structural predictions demonstrate that both of the novel isoforms (*SWEET11a.1* and *SWEET11a.2*) exhibit relatively lower *∆G* values (1.56 and 31.53 kcal/mol) compared to the current canonical reference isoform (329.44 kcal/mol), suggesting higher structural stability for the newly identified isoforms.

Analysis of PFAM domains within the WAT1-RELATED isoforms revealed that the two EamA domains present in the canonical ORF (*WAT1-RELATED.1*) were entirely absent in the novel isoform *WAT1-RELATED.2* (Extended Data Fig. 2a). To evaluate the structural viability of these proteins, we assessed their thermostability by calculating the Gibbs free energy (∆*G*) of folding for structures predicted via AlphaFold3. The canonical WAT1-RELATED.1 protein exhibited a lower (more stable) ∆*G* (17.49 kcal mol^−1^), whereas the novel *WAT1-RELATED.2* isoform showed a much higher ∆*G* of 400.06 kcal mol^−1^ (Extended Data Fig. 2b). These results indicate that the protein encoded by *WAT1-RELATED.2* lacks critical functional domains and is structurally unstable, suggesting it is unlikely to contribute to embryonic vein initiation. In contrast to the *WAT1-RELATED* example, we identified cases where novel isoforms exhibited better structural stability than their canonical counterparts. For instance, in the gene *SWEET11a* (Zm00001eb036880), the canonical protein was predicted to be distinctively more unstable (∆*G* = 329.44 kcal mol^−1^) than the novel isoforms discovered in this study, which demonstrated much lower ∆*G* values (*SWEET1a.1*: 31.52 kcal mol^−1^; *SWEET1a.2*: 1.56 kcal mol^−1^) (Extended Data Fig. 2c). This suggests that some unannotated isoforms may represent the dominant, structurally functional variants in specific developmental contexts.

To further delineate the developmental progression of veins, we compared differentially expressed isoforms among the two embryonic vein tissue domains (embry_v1 and embry_v2) and the more mature coleoptile vein (col_v). Only two isoforms were shared across all three vein domains, whereas 31 differentially expressed isoforms were uniquely shared between embry_v2 and col_v (Supplementary Fig. 4a and Supplementary Table 8). This strong commonality between embry_v2 and mature veins suggests that embry_v2 represents a more mature stage of embryonic vein development than embry_v1. These findings thus indicate that an isoform-level transcriptome may offer the sensitivity required to resolve intricate developmental transitions within the embryonic vein lineage that might be overlooked at the gene level.

### Marker isoforms outline the progression of embryonic vein development

To understand the active biological processes occurring at the embry_v1 and embry_v2 stages of embryonic vein development, we performed Gene Ontology (GO) term annotation for their respective marker isoforms based on the MaizeGDB database^23^. We identified 43 and 24 significantly upregulated marker isoforms (adjusted *P* < 0.05, Bonferroni correction) in the embry_v1 and embry_v2 clusters, respectively (Supplementary Tables 9 and 10). While a considerable proportion of these marker isoforms were single dominant isoforms, with an isoform fraction (IF, the fraction of gene expression contributed by the associated isoform^34^) of at least 0.8, there were notable instances of non-dominant isoforms, suggesting a complex and dynamic splicing landscape.

In the embry_v1 cluster, several marker isoforms were associated with the biological process GO terms ‘auxin efflux’ (GO:0010315) and ‘auxin-activated signaling pathway’ (GO:0009734), including *WAT1-RELATED*, *TERASPANIN-6*, *AAAP71*, *IAA25* and *ATL1* (Supplementary Fig. 4b and Supplementary Table 9). This finding strongly supports the view that embry_v1 is an early stage of vein development heavily influenced by auxin^19,35^. Notably, the majority of these auxin-related isoforms (*WAT1-RELATED.1*, *WAT1-RELATED.3*, *AAAP71.1*, *IAA25.10* and *ATL1.16*) were not dominant (IF < 0.8) (Supplementary Fig. 4g–j), indicating that their roles in embryonic vein development could be highly isoform-specific. Additionally, isoforms of three *BROWN MIDRIB* genes (*BM1*, *BM3* and *BM5*), typically involved in lignin biosynthesis (GO:0009809) for secondary cell walls such as those in xylem tracheary elements^36^, were identified as embry_v1 markers (Supplementary Fig. 4c). Isoforms related to lipid transfer proteins (*IBP1.11*, *ITP1.1* and *PLT20.3*) were also differentially expressed in this cluster (Supplementary Fig. 4f).

For the embry_v2 cluster, marker isoforms included known vein-specific markers such as putative *ZINC FINGER PROTEIN 30* (*ZFP30*; Zm00001eb205550) (Fig. 3f) and *GLUTATHION S-TRANSFERASE* (*GST*) genes^37^ (Supplementary Fig. 4d and Supplementary Table 10). Notably, isoforms from two early nodulin-like protein genes (*ENOD-LIKE 9* and *ENOD-LIKE 3*) were identified as embry_v2 marker isoforms (Supplementary Fig. 4e and Supplementary Table 10). While nodulins are typically associated with symbiotic nitrogen fixation, previous studies reported their expression in non-nodulating plants and accumulation at the phloem sieve element plasma membrane–cell wall interface^38,39^. Given the broad functions of *ENOD-LIKE* genes in cell division and organ development, including phloem^40^, these findings point to a potential role for *ENOD-LIKE* genes in embryonic vein development. Thus, by combining marker isoform identification with spatial histological information, we could confidently associate these isoform clusters with specific embryonic vein developmental domains.

We further explored isoform resolution power by examining specific isoforms of the *ZFP30* gene, which is known to be highly expressed in phloem^1^. Of the 34 *ZFP30* isoforms identified, 7 were highly expressed. Isoforms 7, 8, 38, 40 and 43 were specifically expressed in embry_v1 and embry_v2, whereas isoforms 2 and 37 showed high expression across most clusters (Fig. 3f and Supplementary Fig. 3c). *ZFP30*’s higher expression in embry_v2 suggests that it may be more critical for matured veins, including coleoptile veins. This highlights how alternative splicing regulation for *ZFP30* differs between tissue domains, suggesting higher splicing activities in veins to generate diverse, potentially functionally distinct, isoforms.

### Assessment and spatial localization of novel isoforms

**Extended Data Fig. 3:**
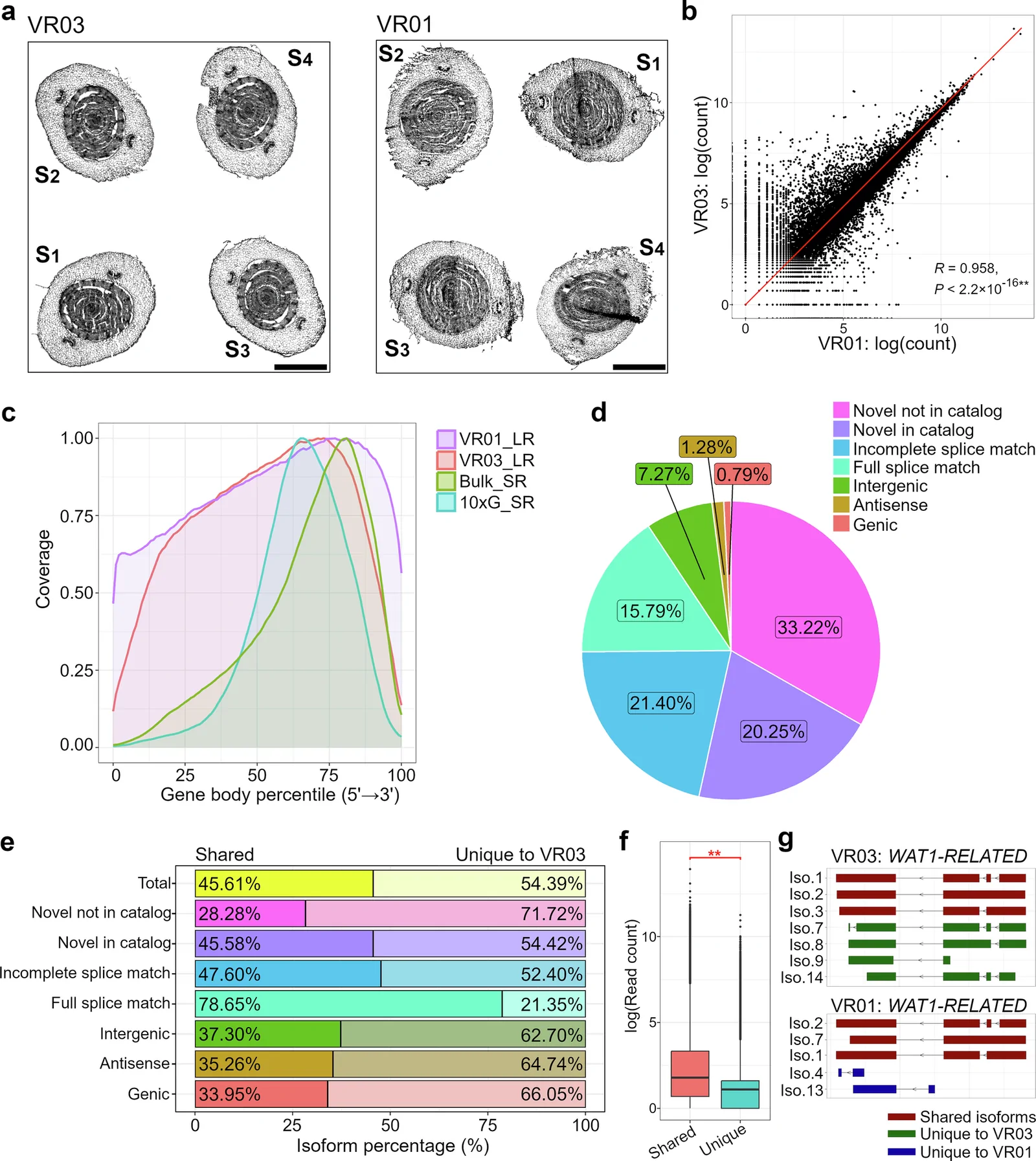
Quality assessment of the biological replicates and isoform validation. **a**, Cross-sectional histology of the maize embryonic shoot samples used in this study: the primary sample VR03 (left) and the independent biological replicate VR01 (right). Labels S1–S4 indicate the sequential order of the four tissue sections from the tip of SAM. Scale bars, 1 mm. **b**, Scatterplot comparing pseudo-bulk expression levels of all genes between samples VR01 and VR03, demonstrating high reproducibility (two-tailed Pearson’s *R* = 0.96, *P* < 2.2×10^−16^**). **c**, Comparison of gene body coverage (5′ to 3′) across samples and sequencing technologies. The long-read (LR) datasets from both VR01 and VR03 exhibit similar and superior coverage uniformity compared to the short-read (SR) datasets, which include LCM bulk RNA-seq and 10x Genomics Visium (VR03) short-read mapping. **d**, Classification of isoform structure categories of the VR01 replicate, highlighting a distribution similar to the primary VR03 dataset. **e**, Percentages of isoforms shared between VR03 and VR01, categorized by total isoforms and individual structural catergories. **f**, Boxplot comparing the read counts of long-read transcripts shared between samples (n = 130,284) versus those unique to VR03 (n = 155,354). Asterisks indicate significant difference based on a two-sided Wilcoxon rank-sum test (*W* = 6,546,863,309, *P* < 2.2×10^−16^**). The center line of the boxplot indicates the median, the lower and upper hinges represent the 25th and 75th percentiles, and the whiskers extend to the minimum and maximum values within 1.5 times the interquartile range. **g**, Isoform models of the *WAT1-RELATED* gene in both samples. Color coding indicates the isoforms shared between samples (red) and those unique to VR03 (green) or VR01 (blue). (Note: the isoform IDs are assigned independently between the two datasets.).

To provide biological affirmation of the novel isoforms identified in our primary dataset (sample VR03), we performed MAS-IsoSeq on an independent biological replicate (sample VR01) from the same experimental batch as described by Wu et al.^41^. These two replicates (VR03 and VR01) were collected at equivalent developmental stages and shared identical tissue compositions, each comprising four consecutive serial sections from the tip of the SAM (Extended Data Fig. 3a). Gene expression profiles between the two replicates were highly correlated (Pearson’s *R* = 0.958, *P* < 2.2 × 10^−16^; Extended Data Fig. 3b). This consistency extended to the long-read datasets, where gene body coverage and the distribution of isoform structural categories were nearly identical between samples (Extended Data Fig. 3c,d). Overall, the findings were highly reproducible, with 45.61% of all isoforms identified in VR03 also captured in VR01 (Extended Data Fig. 3e). The highest reproducibility was observed in the ‘Full Spliced Match’ category (78.65% shared), whereas the NNC category showed the lowest overlap (28.28%) (Extended Data Fig. 3e). Furthermore, the number of reads for shared isoforms was significantly higher than for those unique to VR03 (Wilcoxon rank-sum test (*W* = 6,546,863,309, *P* < 2.2 × 10^−16^); Extended Data Fig. 3f). For the *WAT1-RELATED* gene, all three isoforms identified in VR03 were consistently detected in the replicate; the canonical isoform (Iso.1) and two novel isoforms (Iso.2 and Iso.3) exhibited IFs of 0.75, 0.22 and 0.03, respectively (Extended Data Fig. 3g).

**Extended Data Fig. 4:**
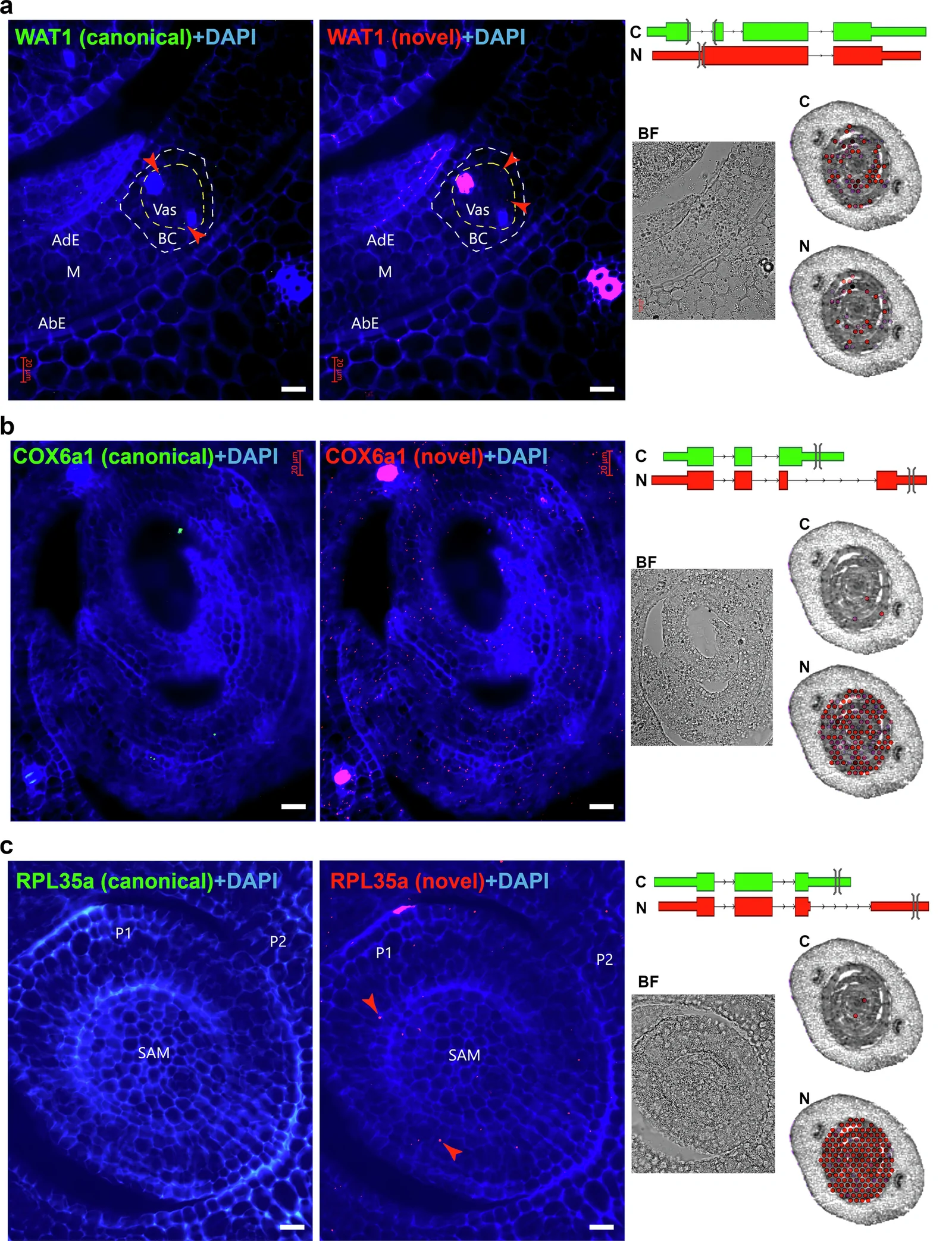
In situ expression patterns of target genes and isoforms revealed by rolling-circle amplification (RCA) of padlock probes. Each subfigure comprises two main microscopic images: the canonical isoform with DAPI (left) and the novel isoform with DAPI (middle). The right panel comprise of the bright-field, structural illustrations of the canonical and novel isoform (labeled with ‘C’ and ‘N’, respectively). The location of designed probes indicated by gray brackets, together with their respective long-reads expression levels on spatial maps. Each image is the representative of at least two independents in situ *hybridization* experiments yielding similar results for each probe. **a**, Both the canonical and novel *WAT1-RELATED* isoforms were highly expressed in vascular tissues; however, the novel isoform displayed a more spatially restricted expression pattern within the vasculature, consistent with the 10x spatial transcriptomics data. **b**, *COX6a1* shows sparse signals from the canonical isoform, while the novel isoform exhibits broader signal distribution in the developing leaf primordia. **c**, *RPL35a* shows signals for the novel isoform across the leaf domains, whereas no clear signal is observed for the canonical isoform, likely due to low read abundance. Red arrows indicate rolling circle amplification products. BF: bright field; SAM, shoot apical meristem; P1, the youngest leaf primordium; P2 and P3, the second and third developing leaf primordia; AbE, abaxial epidermis; AdE, adaxial epidermis; M, mesophyll cell; BC, bundle sheath cell (outlined with a white dashed line); Vas, vascular tissue (outlined with a yellow dashed line). Scale bars = 20 µm.

We further validated the presence and spatial distribution of isoforms with novel splice junctions or novel exons for three target genes using padlock-probe rolling circle amplification (RCA) in situ hybridization. This included specific probes for *WAT1-RELATED*, *CYTOCHROME C OXIDASE SUBUNIT 6a* (*COX6a1*; Zm00001eb058950) and *60S RIBOSOMAL PROTEIN L35a* (*RPL35a*; Zm00001374200). For *WAT1-RELATED*, both the canonical isoform (WAT1-RELATED.1) and the novel isoform (WAT1-RELATED.2) were highly detected in vascular tissues, in agreement with the 10x spatial transcriptomic data. However, the novel isoform displayed a more spatially restricted expression pattern within the vasculature, while the canonical isoform was more broadly expressed (Extended Data Fig. 4a). For *COX6a1*, the novel isoform containing a novel 3′ exon showed a stronger and broader signal in the developing leaf primordia than its canonical counterpart (Extended Data Fig. 4b). Similarly, the novel isoform of *RPL35a*, which also features a novel 3′ exon, demonstrated clear signals in early developing tissues, whereas the canonical isoform was not detected, probably due to low transcript abundance (Extended Data Fig. 4c).

### Isoform-level weighted gene co-expression network analysis uncovers stage-specific regulatory modules

**Fig. 4:**
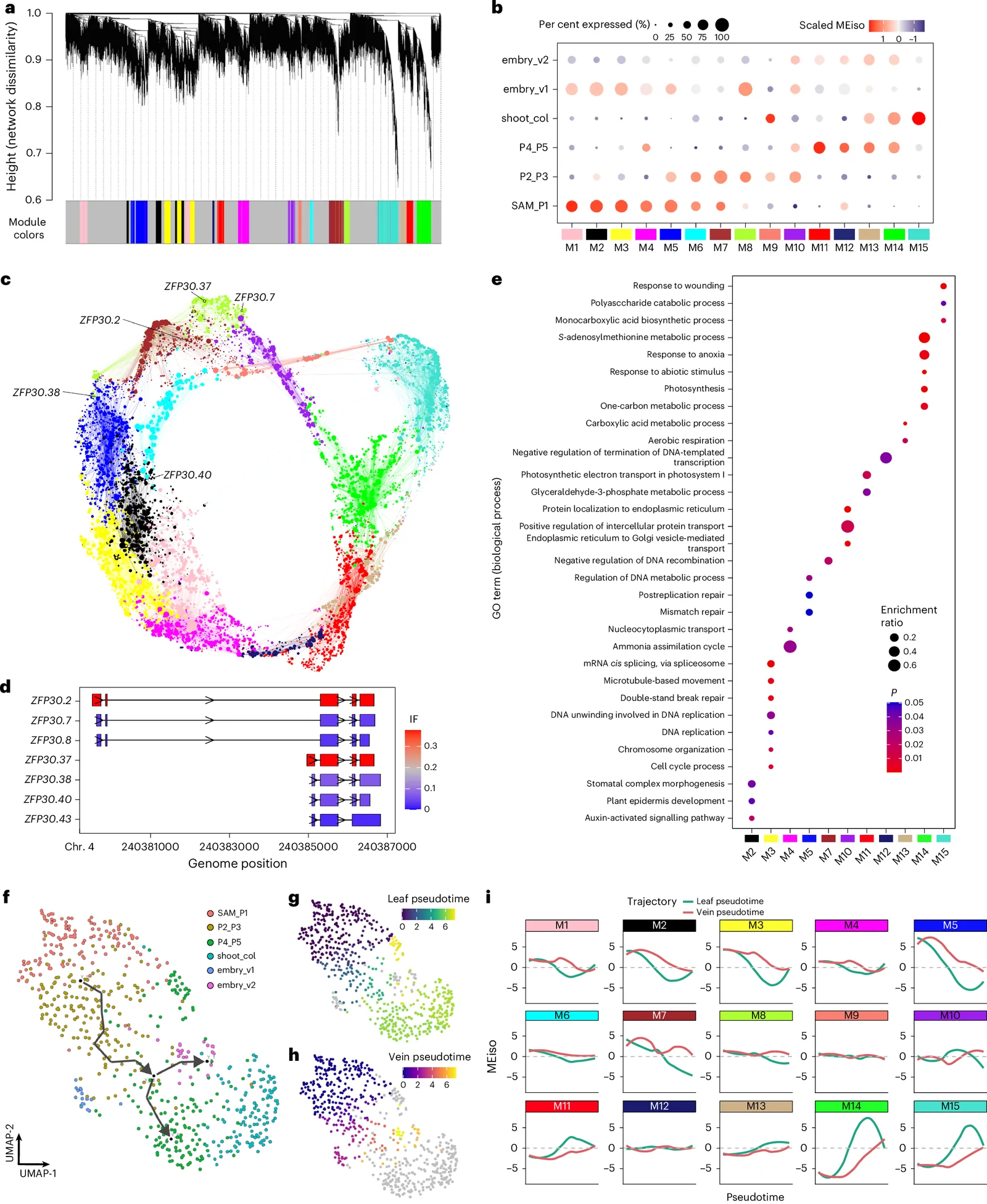
WGCNA of isoform expression data and pseudotime trajectory analysis. **a**, Fifteen modules from a WGCNA of the isoform expression data. **b**, Bubble plot showing the relationship between MEiso values and the clusters identified from unsupervised clustering. **c**, UMAP plot of the isoform co-expression network. Each node represents a single isoform, and the edges are co-expression links between module hub isoforms. The isoforms of *ZFP30* are specifically marked with the colour corresponding to their respective modules’ colour. **d**, IFs of *ZFP30* isoforms. *ZFP30.2* and *ZFP30.37* (bright red) show higher IFs than the other isoforms across the whole tissue section. **e**, Functional enrichment analysis hub isoforms of each module calculated via Fisher’s one-tailed test. Hub isoforms from 12 modules were found enriched in biological process GO terms. **f**, Pseudotime analysis performed on clusters of leaf tissues. The arrows on the UMAP indicate the pseudotime trajectory branching out towards the P4_P5 and embry_v2 clusters. **g**,**h**, UMAP plots for primordia leaf tissue (**g**) and embryonic vein (**h**) are coloured by pseudotime assignment, with the node at the SAM_P1 cluster selected as the principal node. **i**, Trajectories for primordial leaf (lime-green line) and embryonic vein (salmon line) development across the pseudotime. The MEiso value on the *y* axis correlates with the expression level of co-expressed isoforms within the developmental pseudotime.

To further explore the expression profiles and biological functions within each unsupervised cluster, we conducted weighted gene co-expression network analysis (WGCNA)^42^ at the isoform level. While marker isoforms provided initial insights, WGCNA captured the intricate co-expression relationships among isoforms. This analysis identified 15 distinct co-expression modules (Fig. 4a and Supplementary Table 11) from 17,722 isoforms. The remaining isoforms were unassigned to any co-expression modules because they were either lowly or not expressed in the majority of the spots, or because they exhibited weak correlations with other isoforms’ expression profiles. Each module was represented by a module eigen-isoform (MEiso), reflecting its overall expression profile.

The scaled MEiso values clearly highlighted module specificity within certain clusters (Fig. 4b). For example, modules M1 to M7 showed high MEiso values in the SAM_P1 cluster, while M15 was noticeably higher in the shoot_col cluster. Co-expression modules specific to the same cluster tended to cluster together in the UMAP plot (Fig. 4c). For instance, SAM_P1-specific modules (M1, M2, M3, M4, M5, M6 and M7) were spatially proximal in the 2D UMAP space. Each co-expression module comprised ‘hub isoforms’ (isoforms whose expression levels are highly correlated with those of other isoforms within their module), which potentially play a pivotal role in the module’s biological processes. We confirmed that the top 25 hub isoforms of each module exhibited the highest expression in their specified cluster (Supplementary Fig. 5). A gradual transition pattern was observed for all 15 co-expression modules’ MEiso values across the developmental stages of the embryonic shoot (SAM_P1 → P2_P3 → P4_P5 → shoot_col) (Fig. 4b and Supplementary Fig. 5).

Functional enrichment analysis of hub isoforms from SAM_P1 cluster modules (M2, M3, M4 and M5) revealed enrichment for biological process GO terms such as ‘cell cycle process’, ‘chromosome organization’, ‘DNA replication’ and ‘auxin-activated signaling pathway’ (Fig. 4e). This finding suggests that the SAM and early primordium leaf stages are actively engaged in cell differentiation. Photosynthesis-related GO terms were enriched in modules associated with the P4_P5 and shoot_col clusters (Fig. 4e and Supplementary Table 12), implying that photosynthetic genes are already expressed even before exposure to sunlight. However, we did not identify any modules exclusively specific to the embryonic vein clusters (embry_v1 and embry_v2), probably due to the limited number of spatial spots in these embryonic vein tissues.

Finally, we found that isoforms of a gene can be assigned to distinct co-expression modules; indeed, 2,652 out of 12,115 genes demonstrated this characteristic (Supplementary Fig. 5 and Supplementary Table 11). For example, for *ZFP30*, isoforms *ZFP30.2* and *ZFP30.27* were assigned to M7 (brown nodes) and M8 (green-yellow nodes) modules, respectively, despite having similar IFs (Fig. 4d and Supplementary Table 11). Different isoforms from the same gene can thus exhibit distinct expression patterns, highlighting the importance of isoform-level resolution in network analysis.

### Pseudotime analysis reveals divergent leaf and vein trajectories

To examine the dynamic gene expression changes across embryonic leaf and vein tissues, we applied the principal graph algorithm from Monocle 3 (ref. ^43^) to the UMAP projection (Fig. 4c). The SAM_P1 cluster was designated as the trajectory ‘root’, reflecting the role of the SAM as the central stem cell reservoir^44^. This designation was corroborated by CytoTRACE^45^, which estimated the differentiation states across all tissue domains (Supplementary Fig. 6).

The pseudotime analysis revealed a clear bifurcation from the primary developmental path (Fig. 4f). The main trajectory progressed sequentially from SAM_P1 to P2_P3, then P4_P5 and finally to the shoot_col clusters (Fig. 4g). This primary path captures the progressive maturation of leaf primordia as they differentiate from the SAM and develop towards the mesophyll of matured leaves. A second, divergent path branched from the common progenitor state (SAM_P1 to P2_P3) into embry_v1 and finally embry_v2. This trajectory represents the vein embryogenesis process, transitioning from early primordia into a newly differentiated embryonic vein stage (embry_v1), followed by a more mature stage (embry_v2) (Fig. 4h). Despite the limited number of spatial spots in the vein domains, the trajectory analysis successfully identified a distinct forked path associated with embryonic vein development.

We further compared these trajectories by examining the activity of isoform co-expression modules across pseudotime (Fig. 4i). For example, the M2 module contains isoforms primarily involved in the ‘auxin-activated signaling pathway’ (Fig. 4c). This module’s expression profile (represented by its MEiso) reaches a maximum during the early pseudotime stages associated with SAM differentiation, followed by a rapid decline as the tissue transitions into leaf primordia. Conversely, the M2 module in the vein trajectory exhibits sustained activity throughout both the early and intermediate stages. This pattern suggests that auxin-mediated signalling contributes to the initiation of vein embryogenesis well beyond the period of initial SAM differentiation, further validating the established importance of auxin in vegetative organogenesis^19,35,46,47^. Similar sustained activity patterns were observed in modules M3, M5 and M7, suggesting shared regulatory mechanisms between SAM differentiation and the initiation of vein development.

### MaizeV5_IsoAnn is a comprehensive isoform-annotated gene-model database

To create a more comprehensive maize transcriptome resource to facilitate accurate and comprehensive short-read mapping and quantification, we constructed MaizeV5_IsoAnn, an isoform-annotated gene-model database. This database was built by integrating extended gene models and novel genes into the existing maize B73 RefGen_v5 gene models.

**Fig. 5:**
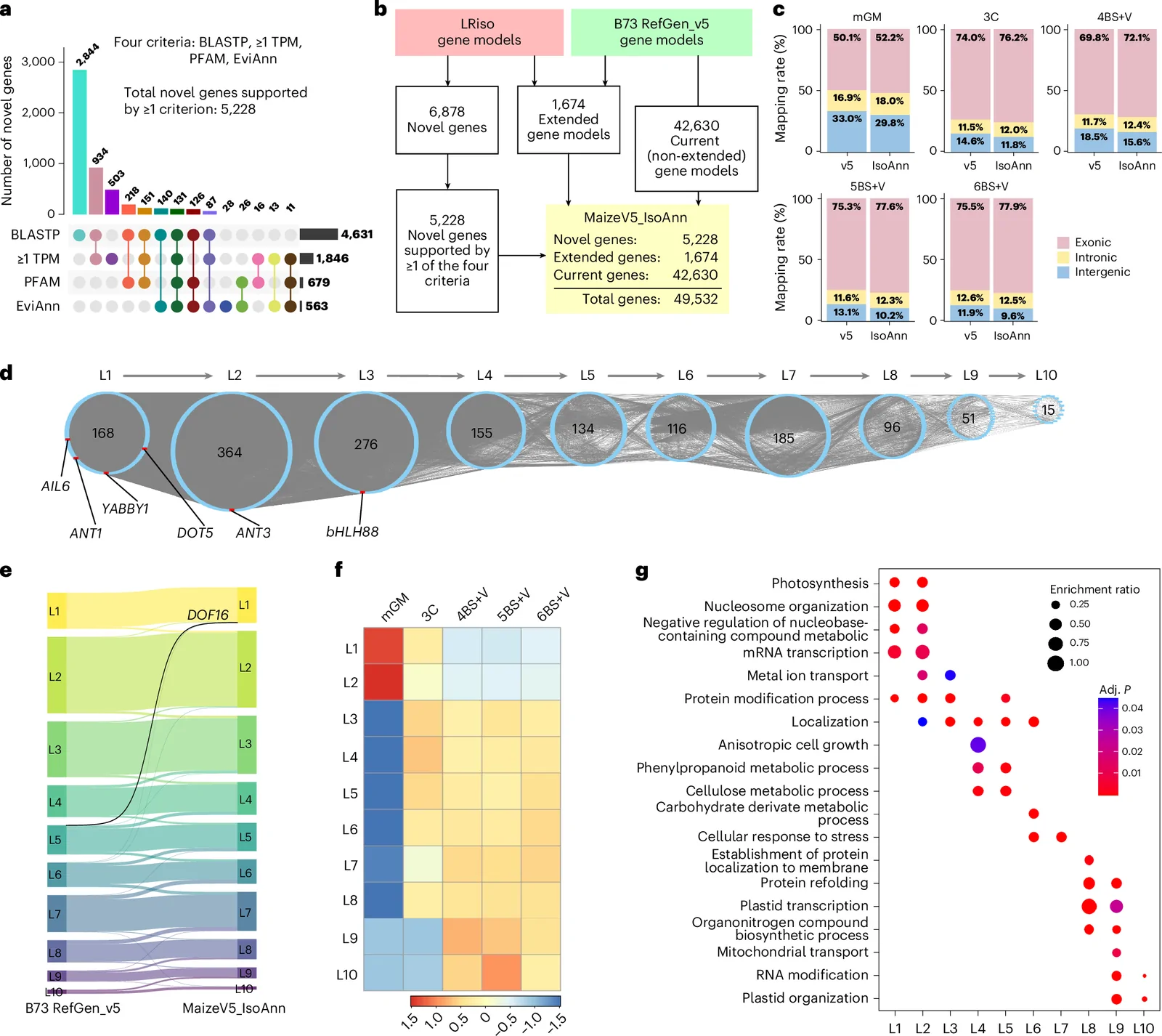
Analyses of short-read transcriptomic datasets of LCM-captured embryonic vein cells using the MaizeV5_IsoAnn gene-model database. **a**, Number of novel genes supported by at least one of four criteria. The top bar chart indicates the numbers of novel genes supported by a single criterion or a combination of two or more criteria. The total number of novel genes with at least one supporting criterion is the sum of all possible cases. **b**, Construction of a new isoform-annotated gene-model database (MaizeV5_IsoAnn). Numbers represent the numbers of gene models. Novel genes and gene models with extensions on 5′- or 3′-flanking regions or both (extended gene models) were extracted from the LRiso gene models and integrated into the MaizeV5_IsoAnn gene-model database. The MaizeV5_IsoAnn gene-model database also includes the non-extended gene models in the B73 RefGen_v5 gene-model database. **c**, Bar charts comparing the region-specific mapping rates using the two different gene models. The *x*-axis labels ‘v5’ and ‘IsoAnn’ represent B73 RefGen_v5 and MaizeV5_IsoAnn, respectively. **d**, TO-GCN of 1,797 maize TF genes (blue nodes). Each circle represents a TO-GCN level, with the number depicting the number of TF genes at that level. TF genes that are known to regulate the initiation of Kranz anatomy development are highlighted in red. **e**, Comparison between TF TO-GCN levels inferred from B73 RefGen_v5 and MaizeV5_IsoAnn reveals TF genes shifted in TO-GCN levels. *DOF16* (black line) is shown as an example. **f**, Heat map of the average normalized TPM at each point of TF genes at each TO-GCN level. The scale bar indicates the expression *Z* score. **g**, GO enrichment using Fisher’s one-tailed test of the co-expressed non-TF genes at each level is indicated by circles in the bubble plot.

Specifically, we identified a total of 1,674 extended gene models that featured 5′- and/or 3′-flanking region extensions compared with RefGen_v5 (Supplementary Table 13). Furthermore, we incorporated 5,228 previously unannotated (novel) genes into MaizeV5_IsoAnn (Supplementary Table 14). These novel genes were rigorously filtered from an initial pool of 6,878 putative novel genes located in intergenic regions, using four criteria to ensure their robustness: protein homology (BLASTP), expression support (≥1 transcripts per million (TPM)), protein domain prediction (InterProScan) and independent annotation (EviAnn) (Fig. 5a, Supplementary Table 15 and Methods).

**Extended Data Fig. 5:**
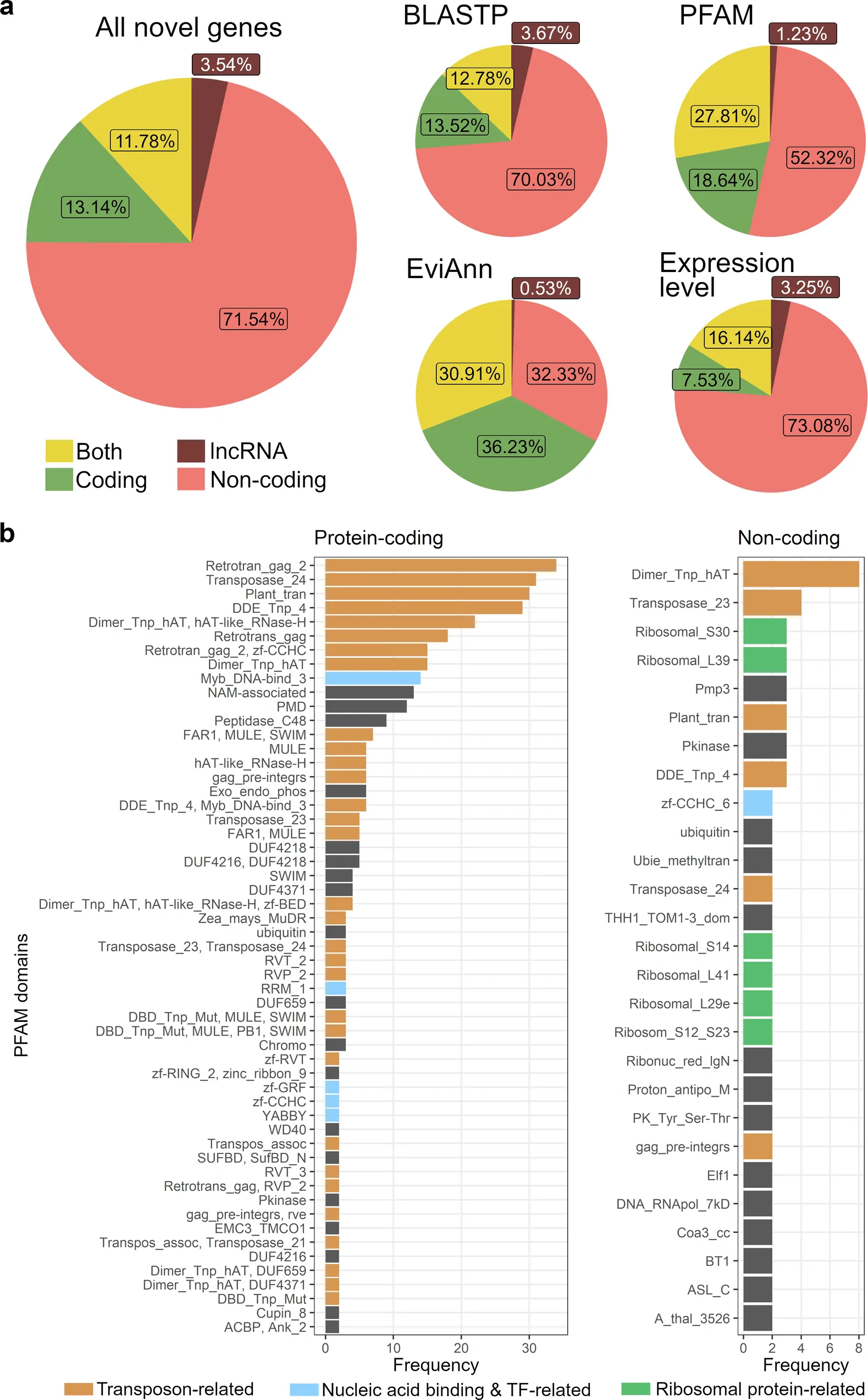
Coding biotypes and functional domain analysis of the novel genes. **a**, Classification and validation of novel genes integrated into the MaizeV5_IsoAnn database. Left panel: Distribution of novel genes categorized by biotype, where the ‘Both’ category represents novel genes containing both Coding and Non-coding isoforms. The fraction of putative lncRNA is also indicated. Right panel: Four pie charts showing the proportion of novel isoforms supported by specific validation criteria: (1) BLASTP hits (E-value ≤ 1 × 10^−5^) against the Viridiplantae protein database; (2) InterProScan functional signatures (E-value ≤ 1 × 10^−5^); (3) evidence-based support from the EviAnn annotation pipeline; and (4) expression (TPM value ≥ 1) in at least six of the 30 laser-captured microdissection (LCM) bulk RNA-seq datasets retrieved from Liu et. al.^19^. **b**, Cumulative frequencies of PFAM domains identified in protein-coding (left) and non-coding (right) novel genes. Bar colors correspond to functional categories: transposon-related (brown), nucleic acid binding and TF-related (blue), and ribosome protein-related (green). Grey bars represent domains with the other or uncharacterized functions.

Among the 5,228 novel genes identified, 1,309 (24.9%) were classified as protein-coding genes using the same approach as for novel isoforms (predictions using three protein-coding-potential assessment tools). In contrast, a majority (3,926; 75.09%) were classified as noncoding (Extended Data Fig. 5a). To explore their potential functions, we first characterized PFAM domains within their predicted protein sequences (Extended Data Fig. 5b and Supplementary Table 16). Of the 679 novel genes with PFAM hits, 147 (125 coding and 22 noncoding) were found to harbour transposon-related domains, while 38 (28 coding and 10 noncoding) contain domains associated with nucleic acid binding and transcription factor (TF) activity. Interestingly, ribosomal-protein-related domains were identified exclusively in 18 noncoding novel genes; these potentially represent ribosomal-protein pseudogenes derived from the retrotransposition of ribosomal-protein mRNAs^48,49^.

In parallel, to identify potential long noncoding RNAs (lncRNA) of all the identified novel transcripts, we performed a BLASTn search of all isoform sequences against maize lncRNA (Ensembl Plants 51) from the GreeNC database (accessed in April 2026)^50^. We identified 228 isoforms from 201 novel genes that exhibited significant alignment with known lncRNAs using stringent criteria (query coverage, ≥90%; identity, ≥90%; and *E*-value, ≤1 × 10^−5^) and confirmed 200 of these novel genes as noncoding via PFAM domain scanning (Extended Data Fig. 5a and Supplementary Table 14). Additionally, 1,178 novel isoforms from the previously known genes matched the lncRNA database (Supplementary Table 4), 897 of which lacked detectable PFAM domains, suggesting they may represent previously unannotated lncRNA variants.

Furthermore, to investigate the regulatory landscape of these novel genes, we leveraged the ATAC-seq datasets^33^ used for isoform assessment to identify ACRs in their promoter regions. We found that 1,689 (32.31%) of these genes possess at least one promoter-associated ACR in at least one tissue type. Of these, 358 genes are associated with constitutive ACRs detected across all six tissue types (Supplementary Fig. 7).

Finally, these validated novel genes, the extended gene models and the current (non-extended) B73 RefGen_v5 gene models were integrated to form the MaizeV5_IsoAnn gene-model database (Fig. 5b). This gene-model database encompasses a total of 49,532 genes, representing a profound expansion compared with the 44,304 genes in the B73 RefGen_v5 gene-model database.

### Reanalysis of LCM vein transcriptomes using MaizeV5_IsoAnn

To investigate the transcriptional regulation of Kranz anatomy development, we leveraged the newly constructed MaizeV5_IsoAnn database to reanalyse the 30 LCM-captured embryonic vein RNA-seq datasets of Liu et al.^19^. These datasets represent a developmental time series across five distinct stages of embryonic leaf cell types: median ground meristem (mGM), a three-cell (3C) stage marking vein initiation and three progressive phases of pre-Kranz anatomy (4BS+V, 5BS+V and 6BS+V), characterized by increasing numbers of pre-bundle-sheath (BS) cells and pre-vein cells following unequal periclinal divisions.

We mapped these 30 RNA-seq datasets to both the B73 RefGen_v5 and our MaizeV5_IsoAnn gene-model databases. The MaizeV5_IsoAnn database consistently yielded a 3% increase in the exonic mapping rate across all LCM cell stages (Fig. 5c and Supplementary Table 17), primarily attributed to the inclusion of extended 5′- and 3′-exonic regions and previously unannotated gene regions. This enhancement resulted in 929 genes showing elevated expression levels (≥1 TPM) when analysed with MaizeV5_IsoAnn. However, the principal component analysis plots of the transcriptomic profiles from both gene model references revealed largely similar overall distribution patterns across the embryonic vein cell stages (Supplementary Fig. 8), indicating that while the new gene model improves read mapping and gene quantification, it largely preserves the global transcriptomic relationships.

To assess how MaizeV5_IsoAnn improves the inference of transcriptional regulation, we performed a time-ordered gene co-expression network (TO-GCN) analysis using 1,797 TF genes retrieved from the PlantTFDB database^51^. These TF genes were categorized into ten TO-GCN levels on the basis of their expression profiles across the first five stages of embryonic vein development (Fig. 5d and Supplementary Table 18). The network accurately placed previously implicated TF genes in embryonic vein development, such as *AINTEGUMENTA-LIKE 6* (*AIL6*), *AINTEGUMENTA 1* (*ANT1*), *ANT3*, *BASIC HELIX-LOOP-HELIX 88* (*bHLH88*), *DEFECTIVELY ORGANIZED TRIBUTARIES 5* (*DOT5*) and *YABBY1*, within the earliest three levels (L1–L3) of the TO-GCN^19,35^. Importantly, when compared with a TO-GCN constructed using the B73 RefGen_v5 gene-model database, 176 TF genes exhibited shifts in their TO-GCN levels, and 6 TF genes were exclusively identified with the MaizeV5_IsoAnn database (Fig. 5e and Supplementary Table 19). In particular, the *DOF16* TF (Zm00001eb315950) shifted from L5 to L1 (Fig. 5e), suggesting its role as a much earlier regulator of embryonic vein development than previously recognized.

The gene expression profiles of TF genes across the ten TO-GCN levels revealed three distinct clusters (Fig. 5f): (1) L1 and L2 showed higher TF gene expression at the mGM and 3C stages; (2) L3 to L8 displayed TF gene expression across all developmental stages except mGM; and (3) L9 and L10 TFs were predominantly expressed in the 4BS+V, 5BS+V and 6BS+V stages (Fig. 5f). Functional enrichment analysis of non-TF genes co-expressed with TFs in each TO-GCN level provided further biological insights (Fig. 5g and Supplementary Table 20). The enrichment of photosynthesis-related GO terms in mGM (L1 and L2) was consistent with the presence of mesophyll cells prior to vein initiation^19^. Anisotropic cell growth was enriched at L4, aligning with the cellular elongation required for vein cell differentiation and metabolite transport^52^. Furthermore, plastid-related GO terms were enriched in later TO-GCN levels (L8 and L9: plastid transcription; L9 and L10: plastid organization), reflecting the differentiation of outer cell layers into bundle sheaths, which are rich in photosynthetic plastids during later vein development^53^.

### *LBD26* represents a putative regulator of embryonic vein development

**Fig. 6:**
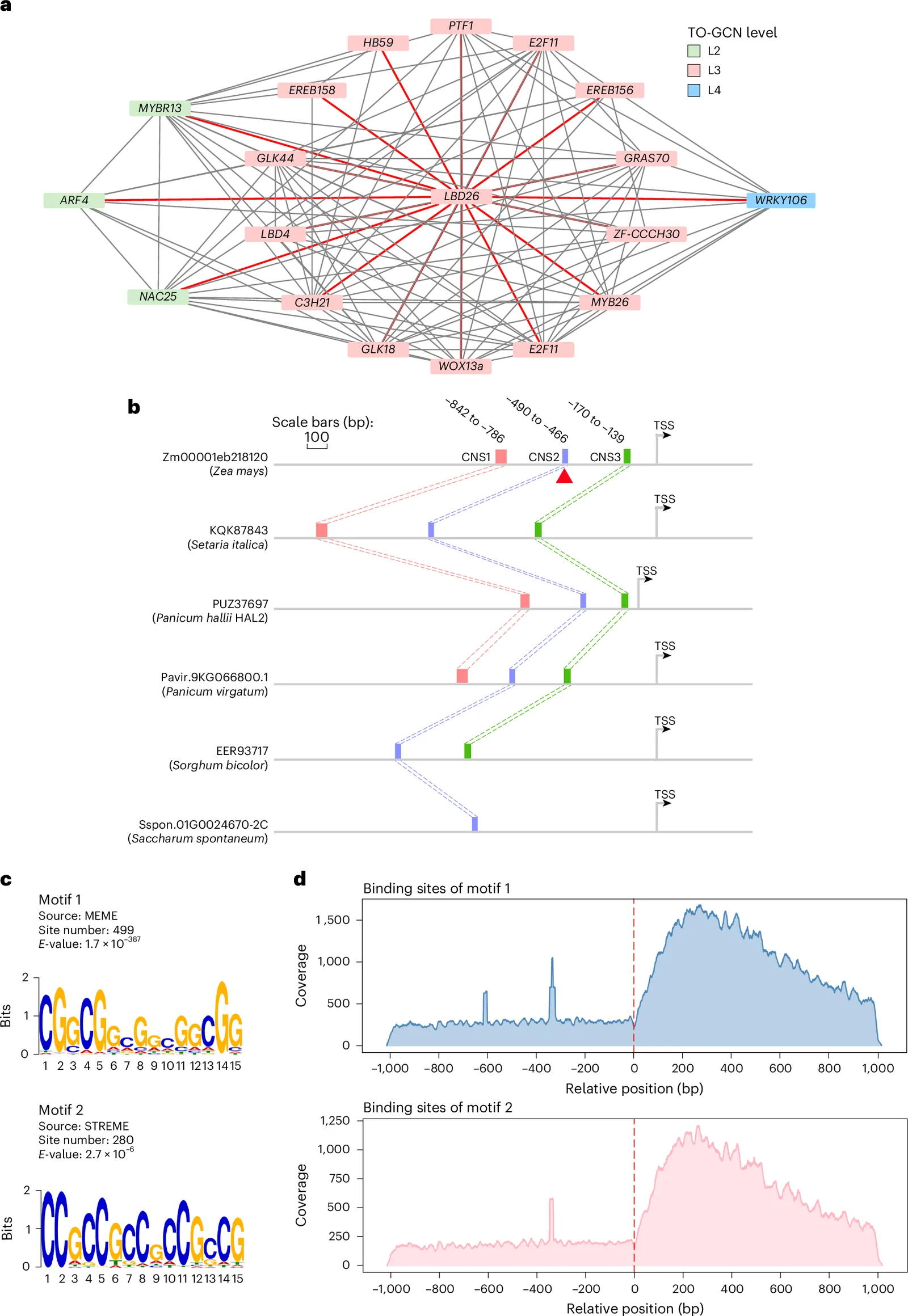
Regulatory network and DNA-binding characterization of the LBD26 protein. **a**, Co-expression network of TFs associated with LBD26. Node colours represent the specific TO-GCN levels assigned to these TFs. Red links highlight direct co-expression associations between *LBD26* and its neighbouring TF genes. **b**, CNSs identified in the promoter regions of *LBD26* homologues across six plant species. The relative positions of three distinct CNSs are mapped on the promoters of each species. The red triangle denotes a predicted binding site for *Z. mays* ARF4. **c**, Identification of two primary DNA-binding motifs for the LBD26 protein via *Setaria italica* DAP-seq analysis. Each motif is supported by more than 100 genome-wide binding site peaks identified across the *S. italica* genome (*E*-value < 1 × 10^−5^). **d**, Distribution of LBD26 binding motifs in the promoter regions (±1,000 bp relative to the TSS) of *Z. mays* genes, using the MaizeV5_IsoAnn annotation. The red vertical dashed line indicates the TSS; the enrichment profile demonstrates substantial binding coverage extending into the first exon (downstream of the TSS).

**Extended Data Fig. 6:**
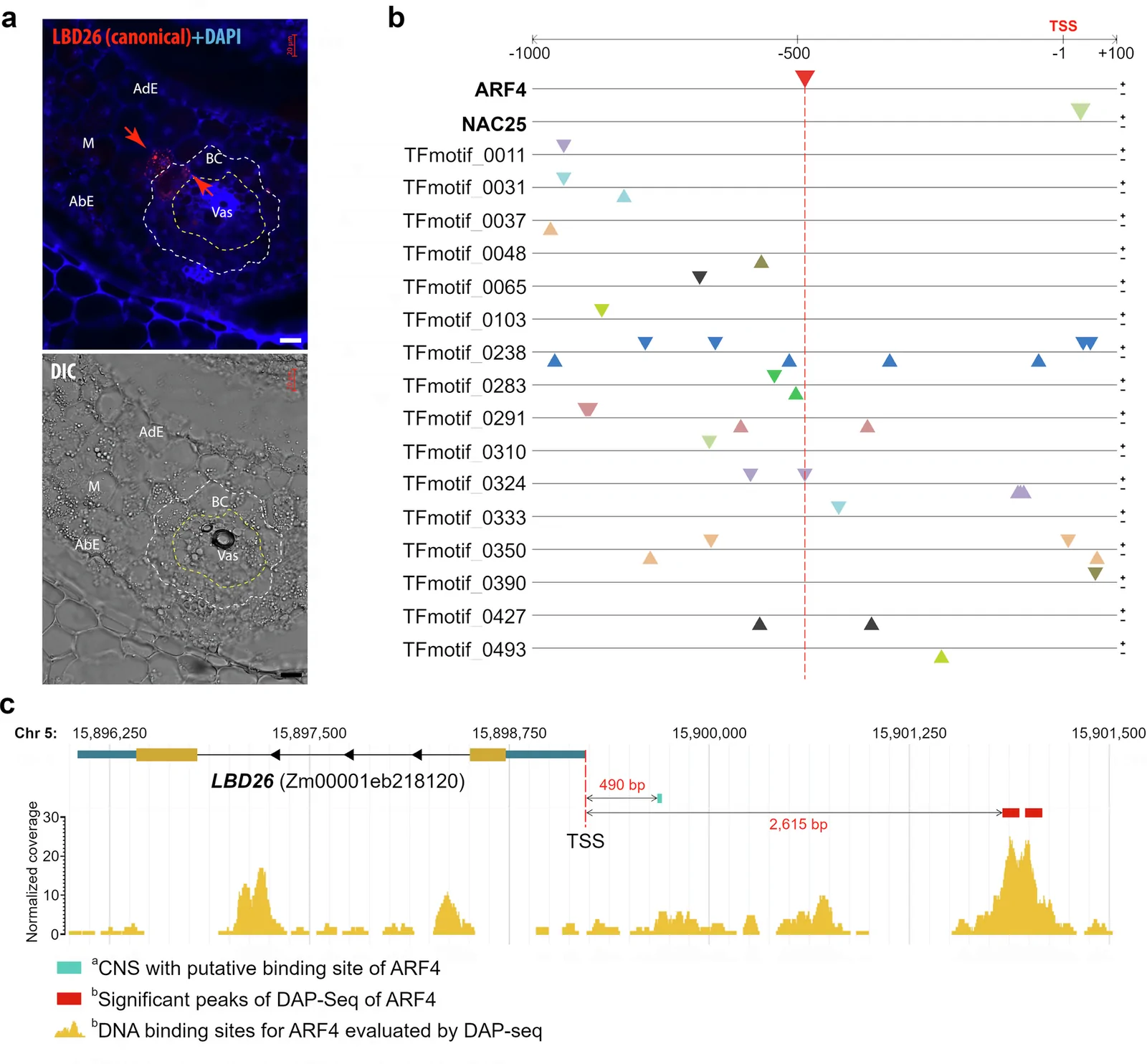
In situ localization of the *LBD26* transcript and characterization of the putative ARF4 binding sites within the *LBD26* promoter. **a**, Localization of *LBD26* transcripts in maize embryonic leaf section using padlock-based in situ hybridization. The signals exhibit highly restricted localization within a bundle sheath cell and an adjacent mesophyll cell proximal to the vascular tissue. DIC: differential interference contrast. Scale bars = 20 µm. The image of in situ hybridization is representative of at least two independents in situ hybridization experiments yielding similar results for each probe. **b**, Transcription factor (TF) binding site analysis within the *LBD26* promoter region (−1000 bp to +100 bp of TSS), retrieved from the PlantPAN4 database. Each binding motif is marked with an inverted triangle (+strand) or a triangle (− strand). The red dashed line highlights a predicted ARF4 binding site matching a TF motif in the promoter region of *LBD26*. **c**, Genome tracks displaying the *LBD26* gene model alongside ARF4 protein binding sites derived from the DAP-seq dataset retrieved from Ricci et al.^55^. Red bars indicate the significant DAP-seq peaks of ARF4 binding, while turquoise bar denotes the *LBD26* genomic location of Conserved Non-coding Sequences (CNS) containing a putative ARF4 binding site. Alphabetical superscripts indicate data sources: (**a**) this study and (**b**) Ricci et al.^55^. Distance from the TSS of *LBD26* to the proximal CNS (490 bp) and the distal DAP-seq peak (2,615 bp) are indicated.

Building on our previous findings, we specifically investigated the putative regulation by the *LATERAL ORGAN BOUNDARIES DOMAINS 26* (*LBD26*) TF (Zm00001eb218120) (Supplementary Table 9), as it was identified as a marker isoform in the early stage of embryonic vein development (the embry_v1 cluster). Previous work on *Arabidopsis thaliana LBD4*, a homologue of *Z. mays LBD26*, suggested its involvement in embryonic vein development and vascular proliferation^54^. To validate the spatial distribution of LBD26 within maize embryonic veins, we performed padlock-probe RCA in situ hybridization. This confirmed the localization of LBD26 transcripts in bundle sheath cells and adjacent mesophyll cells proximal to the vascular tissue. (Extended Data Fig. 6a). Additionally, our TO-GCN analysis assigned *Z. mays LBD26* to L3, suggesting its regulation by upstream TFs such as ARF4 (Zm00001eb067270), MYBR13 (Zm00001eb288890) and NAC25 (Zm00001405590) at L2 (Fig. 6a).

Investigating the TF binding site databases in PlantPAN4.0 (ref. ^55^), we discovered that the promoter region of *Z. mays LBD26* (−1,000 bp to +100 bp relative to the TSS) contains a predicted binding motif for ARF4, as was predicted from TO-GCN analysis, but not for MYBR13 and NAC25 TFs (Extended Data Fig. 6b). This result implicates direct transcriptional regulation of *LBD26* by ARF4. To ascertain the evolutionary conservation of this regulatory interaction, we used PlantPAN4.0 to identify conserved noncoding sequences (CNSs) in the *LBD26* promoter. We identified three CNSs (CNS1, CNS2 and CNS3) that are conserved in all or almost all of five related C_4_ plant species (*Setaria italica*, *Panicum hallii*, *P. virgatum*, *Sorghum bicolor* and *Saccharum spontaneum*) (Fig. 6b). The ARF4 binding site is specifically located in CNS2, and this binding motif was consistently detected in the homologous promoter regions of all five related species. We further validated this regulatory interaction by examining the ARF4 cistrome through a publicly available DNA affinity purification sequencing (DAP-seq) dataset^56^. The analysis revealed two distinct ARF4 binding peaks located 2,615 bp and 2,757 bp upstream of the nearest *LBD26* TSS (Extended Data Fig. 6c), suggesting a potential distal regulatory mechanism. Collectively, these findings strongly suggest that the ARF4 protein regulates *LBD26* expression in *trans*.

**Extended Data Fig. 7:**
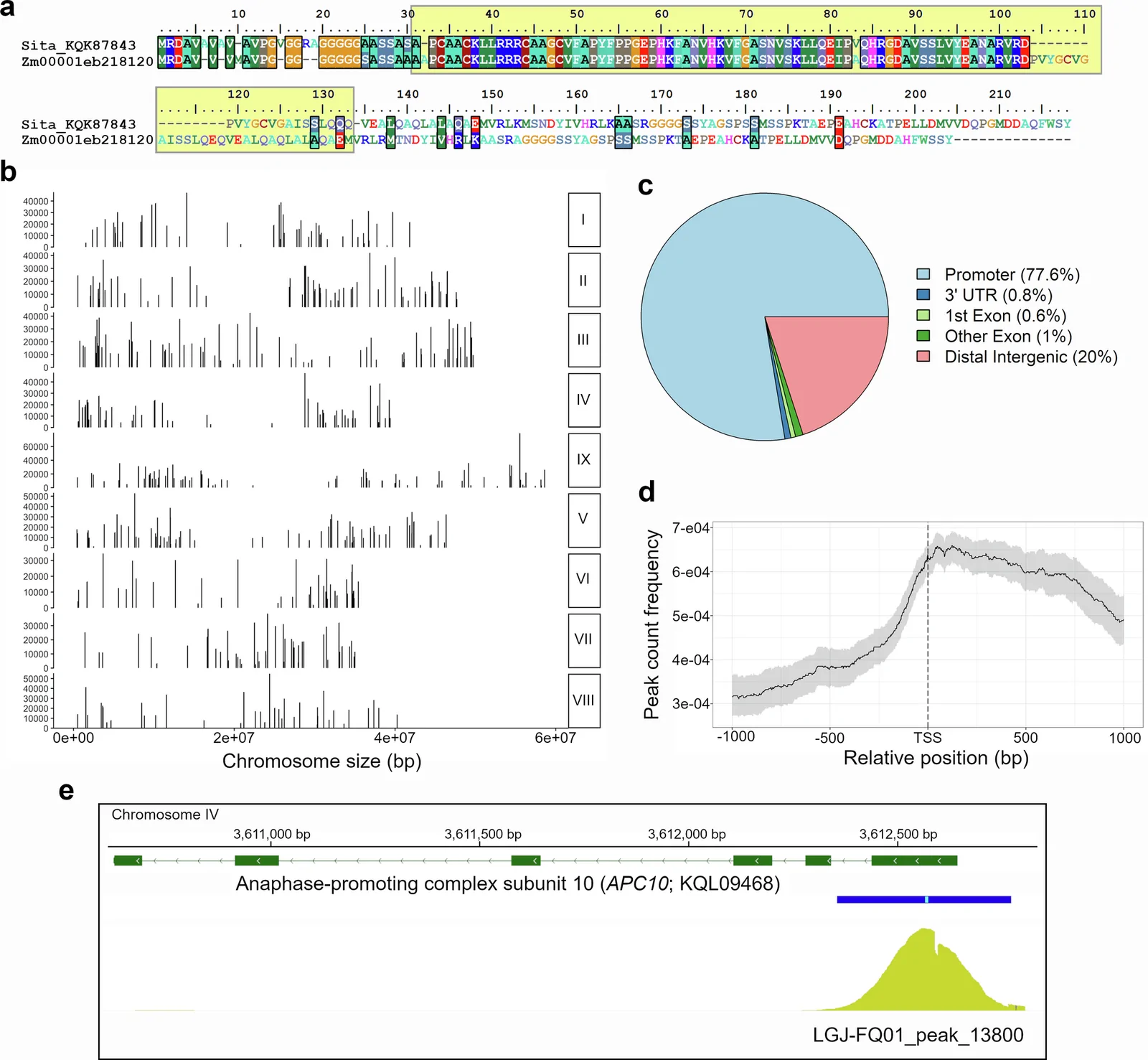
DAP-seq binding profiles of LBD26 protein across the *Setaria italica* genome. **a**, Sequence alignment of the LBD26 proteins from of *S. italica* (Sita_KQK87843) and *Z. mays* (Zm00001eb218120). Conserved and identical amino acid residues between the two sequences are indicated by color-filled boxes. The yellow highlighted region denotes the conserved Lateral Organ Boundaries (LOB) domain in Sita_KQK87843, as annotated by the PFAM database. **b**, Chromosomal distribution of the top 500 LBD26 DAP-seq binding peaks, ranked by summation score, across the nine chromosomes of the *S. italica* genome. The *x*-axis represents chromosomal position (bp), and the *y*-axis indicates peak confidence score (log_10_(*q-value*)*10). **c**, Genomic distribution of the top 500 LBD26 binding sites. The majority (77.6%) of the peaks are located within promoter regions. **d**, Frequency of LBD26 binding peaks relative to the TSS in the target promoter regions ( ± 1,000 bp). Significant peak enrichment is observed within the first exon, immediately downstream of the TSS (0 ~ 1000 bp). The grey shading represents the confident interval estimated by bootstrap method with 1,000 iterations. **e**, Representative JBrowse tracks illustrating a prominent LBD26 binding peak at the promoter region of the *APC10*, a gene involved in xylem and phloem cell wall biogenesis.

To identify the candidate genes potentially regulated by LBD26, we conducted DAP-seq to characterize its binding motifs in the *S. italica* (foxtail millet) genome as we did previously^57^ because we had a millet genomic DNA library, and a genomic DNA library was difficult to construct. To assess the protein-level conservation of LBD26, we performed sequence alignment between maize (Zm00001eb218120) and millet (Sita_KQK87843). The analysis revealed high overall similarity, specifically within the lateral organ boundaries domain, which remains highly conserved across both taxa (Extended Data Fig. 7a). A total of 36,605 binding peaks were identified using MACS3 (ref. ^58^). The top 500 peaks were widely distributed across the nine primary chromosomes of *S. italica* (Extended Data Fig. 7b) and were primarily localized (77.6%) to promoter regions, defined as the ±1,000-bp window flanking the TSS (Extended Data Fig. 7c). Within these promoters, binding peaks showed a notable enrichment in the exonic regions immediately downstream of the TSS (Extended Data Fig. 7d). Analysis of the sequences within 100 bp of the peak summits identified two significant, GC-rich binding motifs (position weight matrices), each supported by at least 100 peaks with an *E*-value ≤ 1 × 10^−5^ (Fig. 6c).

To identify putative LBD26 protein targets in maize, we used FIMO (Find Individual Motif Occurrences)^59^ to scan the promoter regions of all genes in the MaizeV5_IsoAnn database for the two identified putative binding motifs (Fig. 6c). Reflecting the high GC content of these sequences, we identified 2,282 genes containing at least one LBD26 binding motif using a stringent criterion (*q* ≤ 1 × 10^−5^, false discovery rate estimation) (Supplementary Table 21). Consistent with our findings in the millet genome, these motifs were highly enriched within the first exon and 5′ UTR regions of the maize genome (Fig. 6d).

To identify putative targets specifically involved in embryonic vein development, we cross-referenced these candidates with the 86 genes identified as downstream targets of LBD26 in our TO-GCN (levels L3 and L4). Among these, 6 genes were found to contain the putative LBD26 binding motifs, including *ANAPHASE-PROMOTING COMPLEX SUBUNIT 10* (*APC10*, Zm00001eb294590) which is associated with ‘phloem or xylem histogenesis’ (Supplementary Table 22). This regulatory interaction is further corroborated by a DAP-seq peak in the promoter of the *APC10* orthologue in *S. italica* (Extended Data Fig. 7d). Collectively, these results strongly suggest that LBD26 plays a role in embryonic vein development.

## Discussion

This study integrated spatial transcriptomics with high-throughput full-length isoform sequencing (spatial MAS-IsoSeq) to dissect maize embryonic leaf development, with a specific focus on vein differentiation. We leveraged HiFi full-length isoform sequencing in a spatial context to investigate embryonic vein development in *Z. mays*. Our spatial MAS-IsoSeq approach revealed that a remarkable 72.87% of detected isoforms were previously uncharacterized, comprising structural categories of ISM (23.37%), NIC (18.92%) and NNC (30.58%) (Fig. 2a). This high proportion of novel isoforms mirrors recent findings in humans (72.6%)^60^, underscoring a notable deficiency in current gene annotations across diverse organisms.

Further analysis indicated that 38.72% of these novel isoforms are potentially translated into proteins containing functional domains (Fig. 2e). However, our results also suggest a spectrum of biological or stochastic variation, as 45.61% of the isoforms were reproducible between biological replicates (Extended Data Fig. 3e). Given that non-reproducible isoforms generally exhibited lower expression levels (Extended Data Fig. 3f), their detection is probably influenced by Poisson sampling noise; consequently, capturing rare splice variants typically requires higher sequencing depth to reach saturation^61^. Furthermore, we identified 9% of novel isoforms with alternative TSSs associated with proximal ACRs (Extended Data Fig. 1d and Supplementary Table 13), providing valuable insight into the regulatory context for these novel isoforms.

Many of our newly identified isoforms contain novel 3′ exons, with 23.7% supported by the presence of poly-A motif sites (Fig. 2d, Extended Data Fig. 1c and Supplementary Table 13), leading to extensions of their current gene models. This finding holds particular relevance for short-read sequencing platforms, such as 10x Genomics 3′-based single-cell and spatial transcriptomics that rely on poly-A capture for detection and exhibit a 3′-end bias (Fig. 2b). This bias can inadvertently skew differential gene expression analysis, as reads mapped beyond current gene models are usually excluded from quantification (Fig. 2d,e). Our results strongly demonstrate that full-length long-read isoform sequencing offers a robust solution to this pervasive issue, especially critical when quantitative expression levels of target genes deviate from expectations or require robust validation. Additionally, examination of time-course bulk-seq mapping revealed a noticeable proportion of reads aligned outside of the current gene models (Fig. 2b), suggesting that the affected genes may be under-studied and/or lack sufficient supporting information for these novel isoforms to be included in the current B73 RefGen_v5 reference gene-model database.

From the isoform data, we identified 5,228 novel genes, substantially expanding the current maize reference gene database. Unlike short-read sequencing, which is prone to transcript assembly errors due to its inherent limitations in resolving complex or long transcripts, long-read HiFi sequencing generates highly accurate full-length transcripts^62^. This minimizes misalignment issues often associated with short and low-quality reads, ensuring reliable gene discovery. Furthermore, the high throughput of MAS^13^ provided sufficient sequencing depth to robustly support the expression of these novel genes with strong statistical confidence. To ensure the reliability of these discoveries, all putative novel genes underwent a rigorous filtering process (Fig. 5a), and assessments through various analyses confirmed their functional potential (Fig. 5a,b). Consequently, this study provides a comprehensive list of highly supported novel genes expressed in maize embryonic leaves (Supplementary Table 13), opening new avenues for future research into their biological roles.

Previous spatial transcriptomics studies on maize embryonic leaves, using platforms such as LCM^19^, 10x Genomics Visium^1,41^, Xenium^63^, STOmics Stereo-Seq^18^ and Resolve Biosciences Molecular Cartography^16^, offer varying degrees of resolution and transcriptome coverage. However, none of these studies provided isoform-level expression profiling in a spatial context. Our study demonstrates that integrating full-length isoform information enhances spatial spot clustering resolution (Fig. 3d). As demonstrated in this study, alternative splicing events and IR can lead to isoforms with drastically different protein functionalities due to domain gains or losses (Extended Data Figs. 1b and 2a,b), consistent with the report by Wang et al.^7^. Consequently, quantifying gene expression solely by summing across all isoforms may not fully capture a gene’s functional breadth. Our analyses notably improved the accuracy of function-specific clustering (Fig. 3e and Supplementary Table 7), reinforcing the functional relevance of isoform resolution.

Leveraging isoform-level resolution in unsupervised clustering, we successfully identified two distinct clusters (embry_v1 and embry_v2) representing sequential developmental stages of embryonic vein (Fig. 3b–d). This provides insights into the nuanced progression of early vein formation. These two embryonic vein stages exhibited clear differential isoform expression patterns, with the later stage demonstrating transcriptomic features resembling mature leaf veins. For instance, in the early vein initiation stage (embry_v1), the expression of *BM* genes and *WAT1-RELATED* (Supplementary Table 7) aligns with initial xylem and phloem formation via lignin biosynthesis and cell wall development. Concurrently, lipid transfer protein genes facilitate lipid accumulation in developing embryonic veins, potentially contributing to secondary cell wall lipid modification^64,65^.

**Extended Data Fig. 8:**
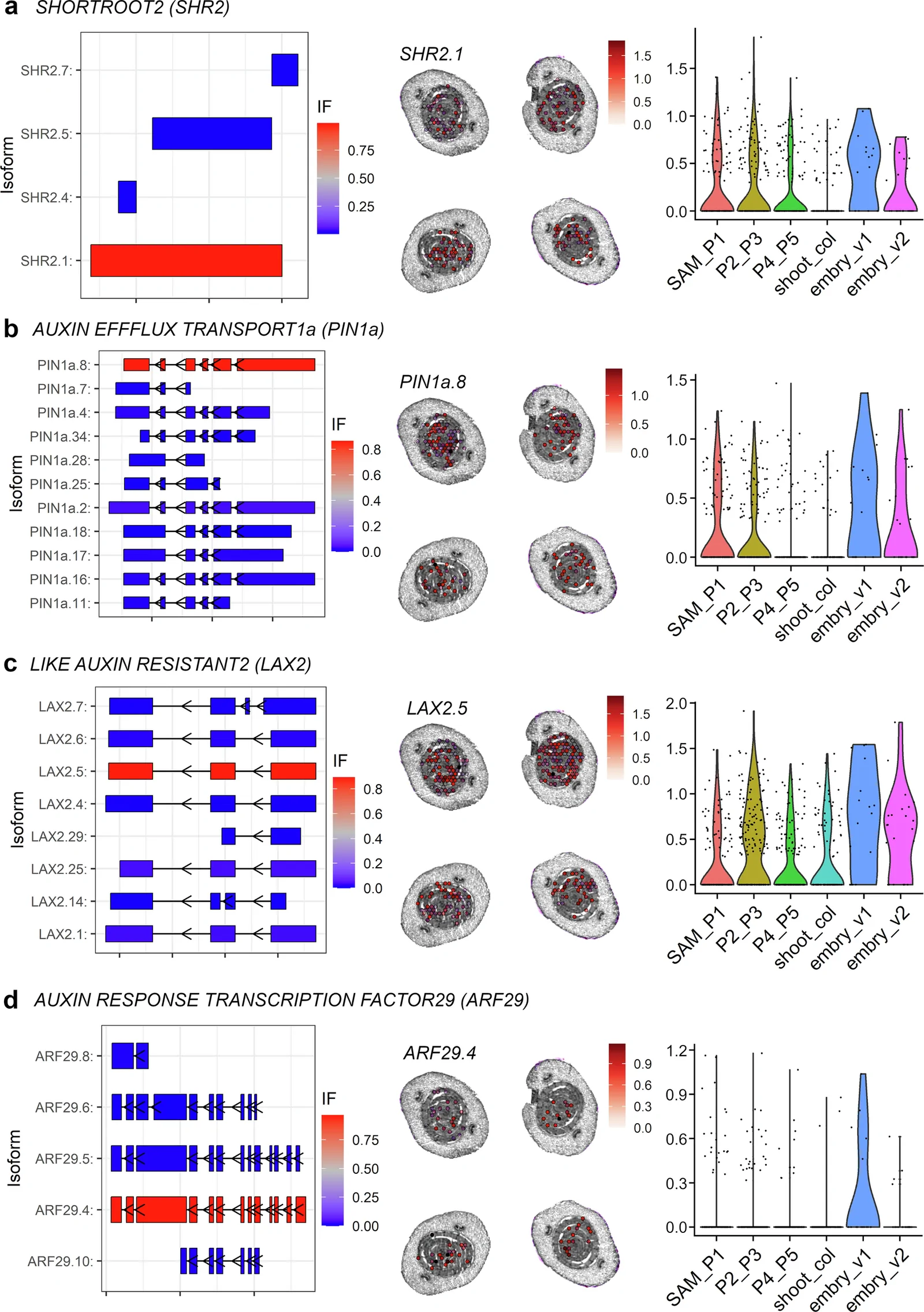
Representative isoforms and expression levels of marker genes associated with vein development. Transcript isoforms and spatial expression patterns are shown for **(a)** *SHR2*, **(b)** *PIN1a*, **(c)** *LAX2*, and **(d)** *ARF29*, which are known regulators of vein development in maize embryonic leaves. The transcript plots (left panel) display the isoform fraction (IF) for each transcript variant. The spatial plots (middle panel) illustrate the localization and expression levels of the single dominant isoform, with the scale bar indicating normalized expression values. The violin plots (right panel) summarize the expression levels across the distinct tissue domains. These marker genes exhibit high expression within embry_v1 and embry_v2 clusters, with the exception of *ARF29* which is uniquely expressed in the embry_v1 cluster.

Our findings are largely consistent with previous single-cell-resolution studies^16,18,41^, confirming the high expression of key vein development and initiation regulators (*SHR2*, *PIN1a*, *LAX2* and *ARF29*) in the early embryonic vein cluster (Extended Data Fig. 8). Similarly, our data corroborate earlier research showing pronounced expression of auxin-associated genes^46^ such as *WAT1-RELATED*, *TERASPANIN-6*, *AAAP71*, *IAA25* and *ATL1* (Supplementary Table 8) in this same cluster, mediating embryonic vein initiation and development. From the marker genes of sub-cell types provided by another single-cell-level spatial transcriptomics study^18^, we found that nine embry_v1 marker genes from this study matched the marker genes from three Kranz anatomy sub-cell types, including procambium, xylem and vascular sclerenchyma 1 (Supplementary Table 23). These concordances demonstrate that while our spatial resolution differs from single-cell approaches, it does not diminish the profound biological insights gained. Instead, our study uniquely provides supplementary isoform-level information that profoundly enriches our understanding of maize embryonic development.

This study markedly expands on previous investigations into the transcription profiles of LCM-isolated developing vein cells^19^, which primarily depicted expression profiles without spatial continuum. Unlike single-cell RNA-seq, which relies on individually isolated cells, our spatial transcriptomics approach provides critical contextual information from neighbouring mesophyll cells and the continuous tissue architecture. Crucially, the MaizeV5_IsoAnn gene-model database enabled a powerful reanalysis of existing LCM-isolated embryonic leaf cell transcriptome datasets. Integrating the extended gene models refined the expression levels of extended gene models, particularly for TF genes, leading to a more accurate reflection of their biological roles (Fig. 5f).

Through the TO-GCN analysis and cross-species CNS analysis, we identified a conserved regulation of *LBD26* by *ARF4* (Fig. 6a). This putative regulation appears critical for the early differentiation stages of embryonic vein development. Furthermore, we identified *APC10* as a gene potentially regulated by LBD26 through TO-GCN and further verified this using DAP-seq (Fig. 5 and Extended Data Fig. 7e), implying its role in promoting vascular formation (Supplementary Table 22). While the regulatory functions of LBD-family TF genes are well studied across various plant species, with diverse roles in organ development, photomorphogenesis and hormone response^66–68^, the specific role of maize *LBD26* in C_4_ plant embryonic vein development remained unclear despite its reported high expression in embryonic veins^18^. Our findings provide compelling evidence to hypothesize that the *LBD26* TF plays a crucial and evolutionarily conserved role during embryonic vein development in maize.

While this study demonstrates the power of integrated spatial and full-length isoform sequencing, certain inherent limitations should be acknowledged. First, although our sequencing depth effectively resolved developmental profiles and UMAP topology, spatial coverage was limited to 615 barcoded spots across four tissue slices within a single capture area. This sample size may restrict the statistical power required for advanced analyses, such as comprehensive differential transcript usage^69^. As an initial application of spatial MAS-IsoSeq in plants, we prioritized optimal tissue quality and histological staining for proof of concept over broader spatial scale. Second, the 55-µm spot diameters and 45-µm inter-spot gaps hinder the capture of fine-scale cellular transitions and may result in mixed signals from adjacent tissues. We therefore could not draw definitive conclusions regarding narrow vascular structures comprising fewer than ten cells, such as specific xylem or phloem lineages. Furthermore, transcripts within inter-spot gaps may be lost or under-represented. While these factors currently restrict cellular-level resolution of vein initiation, rapidly advancing technologies—including Visium HD^70^ and other sub-cellular high-throughput spatial platforms—are expected to overcome these constraints, enabling even finer dissection of plant developmental processes. Lastly, while our DAP-seq data provide valuable in vitro evidence of the DNA-binding capacity of LBD26, a recognized limitation of this study is the lack of in vivo functional validation, such as mutant analysis. Future functional genetic characterization and stable mutant analyses will be essential to definitively map the in vivo regulatory networks governed by LBD26 during embryonic vein development.

## Methods

### Plant tissue preparation, cryosectioning and staining

The samples used in this study are also described in Wu et al.^41^. Briefly, maize seedlings were dissected after 72 hours of imbibition. The embryonic tissues were embedded in Optimal Cutting Temperature compound (Sakura Finetek) and immediately solidified by immersion in liquid nitrogen to preserve RNA integrity and tissue structure. The embedded tissue was cryosectioned at 12-µm thickness horizontally to include the SAM in the centre. Four serial sections were carefully placed within a capture area on a Visium Spatial GE slide (10x Genomics). For histological imaging, the fresh-frozen tissue sections on the GE slide were double-stained with hematoxylin and eosin, and images were acquired using a TissueGnostics Slide Scanner (TissueGnostics). To confirm RNA quality prior to library preparation, several adjacent cryosections were collected for total RNA extraction, and quality was assessed on a Fragment Analyzer (Agilent), consistently yielding high RNA integrity number values of approximately 9.0.

### 10x Genomics Visium spatial transcriptomics library preparation and sequencing

The 10x Genomics Visium libraries were constructed using the Visium Spatial GE slides and Reagent Kit (Cat. No. PN-1000184, 10x Genomics), Visium Accessory Kit (Cat. No. PN-1000194) and Dual Index TT Set A (Cat. No. PN-1000215) following the manufacturer’s recommendations. On the basis of our tissue optimization tests, tissue sections on the Visium GE slides were permeabilized for 15.5 minutes at 37 °C using the Visium Permeabilization Mix (Cat. No. PN-1000191). Released polyadenylated mRNA was captured by oligo-dT primers containing spatial barcodes and UMIs directly on the GE slides. Captured mRNA then underwent reverse transcription with 5′-template-switching oligonucleotides, followed by second-strand synthesis and cDNA amplification. Full-length cDNA was purified using the SPRIselect Beads Reagent Kit (Beckman Coulter), quantified using the Qubit HS DNA Assay (Thermo Fisher) and profiled using the Fragment Analyzer DNA Assay (Agilent). A precisely measured aliquot of amplified cDNA (83.2 ng, corresponding to approximately one quarter of the total amplified cDNA) was then used for downstream next-generation sequencing library preparation, which included DNA fragmentation, end repair, A-tailing, ligation with dual-index adapters and 11 cycles of PCR amplification to yield the final Visium cDNA library. The final library fragment size was maintained at approximately 500 bp, consistent with 10x Genomics recommendations. Visium libraries were sequenced on an Illumina NextSeq 2000 system (Illumina) at the High Throughput Genomics Core, Biodiversity Research Center, Academia Sinica, Taiwan.

### MAS-IsoSeq library preparation and PacBio HiFi sequencing

To obtain spatially resolved full-length isoforms, the remaining full-length cDNA (150 ng) from the 10x Genomics Visium workflow, which retained the spatial barcodes and UMIs and had a size range of 600 to 3,000 bp, was used for MAS-Seq library preparation. Two independent MAS-IsoSeq libraries were prepared and sequenced (Run IDs: QD003, QD010 and QD011; Supplementary Table 1) to ensure comprehensive transcript coverage. The library preparation followed the standard protocol for MAS-Seq for the 10x Single Cell 3′ Kit (Cat. No. 102-659-600, PacBio).

For MAS-IsoSeq library construction, the 10x Genomics cDNA library was initially amplified via a three-cycle PCR program (initial denaturation at 98 °C for 3 minutes; three cycles of 98 °C for 20 seconds, 65 °C for 30 seconds and 72 °C for 4 minutes; followed by a final extension at 72 °C for 5 minutes). To mitigate potential template switch oligonucleotide artefacts generated during 10x Genomics Visium cDNA amplification, the product was subsequently subjected to capture with MAS beads. The resulting product was then evenly distributed into 16 parallel reactions and amplified using unique MAS primer pairs to generate cDNA fragments containing orientation-specific MAS segmentation adapter sequences. Through enzymatic nicking at the MAS adapters with the MAS SMRTbell adapter, these 16 cDNA fragments were assembled into linear arrays with an average length of 13,000 bp, flanked by SMRTbell hairpin adapters at both ends (Fig. 1c–e). The MAS library underwent DNA damage repair and nuclease treatment for final clean-up and removal of incomplete arrays prior to sequencing. The prepared MAS library was then loaded onto three 8M SMRT cells for HiFi sequencing on a PacBio Sequel IIe system (PacBio), with a 30-hour movie collection time per cell. All MAS-IsoSeq preparation and HiFi sequencing were performed at the NGS High Throughput Genomics Core, Biodiversity Research Center, Academia Sinica, Taiwan.

### Long-read transcriptomic data processing and isoform classification

**Extended Data Fig. 9:**
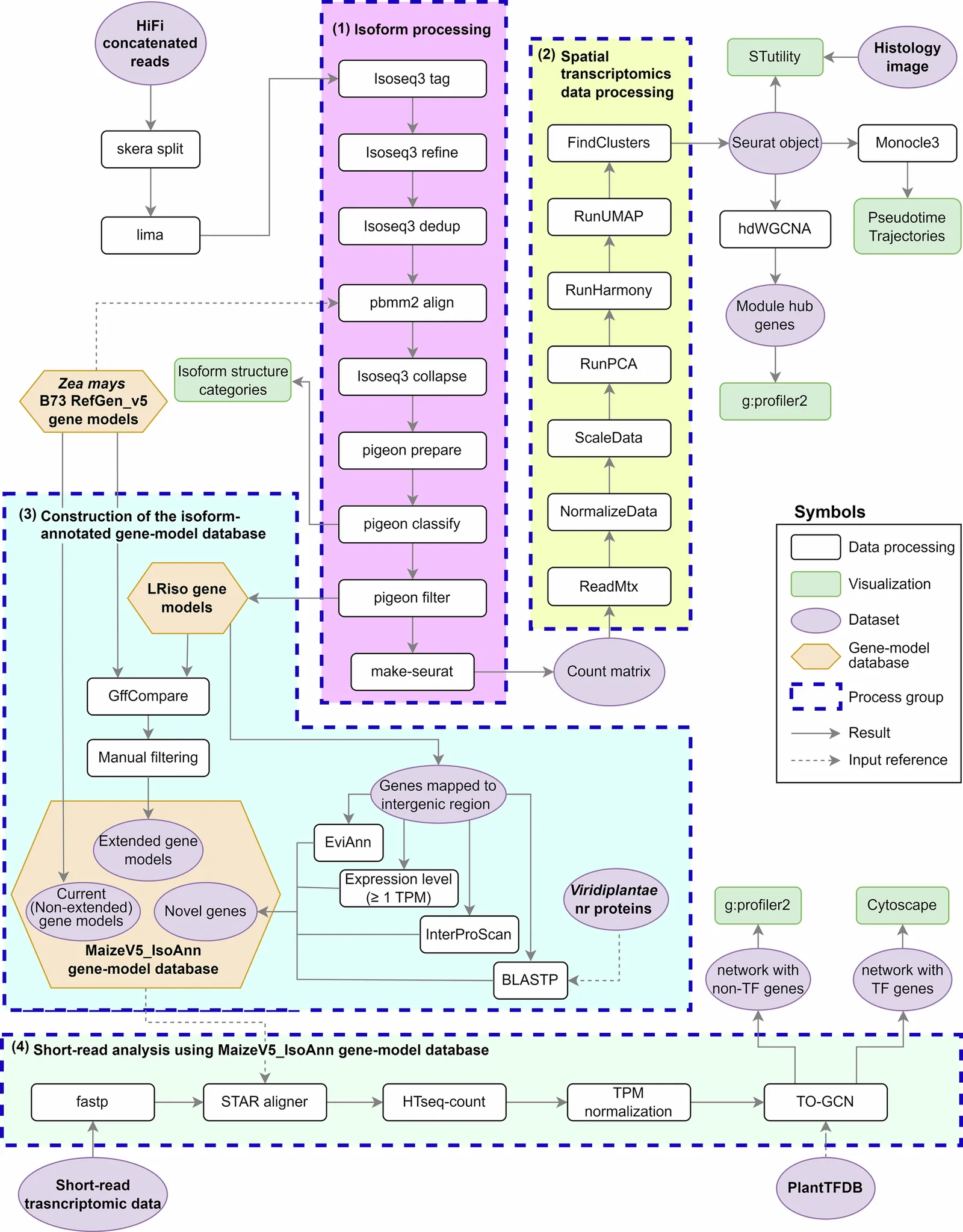
The schematic bioinformatics workflow of the spatial MAS-IsoSeq. The workflow is organized into four primary modules, each delineated by colored blocks with blue dashed borders. (1) Transcript Processing and Isoform Discovery: Raw data are processed using the PacBio IsoSeq and Pigeon pipelines to generate the LRiso gene-model database from identified full-length isoforms. (2) Spatial Transcriptomic Analysis: The resulting isoform-count matrix is utilized for spatial clustering and tissue domain characterization using the Seurat R package. (3) Database Integration: A comprehensive isoform-annotated database, MaizeV5_IsoAnn, is constructed by integrating novel genes and extended models from the LRiso database with non-extended reference models from the B73 RefGen_v5 genome. (4) Integrative Short-Read Analysis: The MaizeV5_IsoAnn database serves as a refined reference for re-analyzing short-read transcriptomic datasets and performing TO-GCN analysis to infer developmental regulatory dynamics.

The bioinformatics workflow for spatial MAS-IsoSeq data processing is summarized in Extended Data Fig. 9. Raw concatenated HiFi reads from the PacBio Sequel IIe system were initially processed using standard SMRT Link software tools (v.12.0)^71^. Quality control for the output of the three MAS-IsoSeq runs was performed using the built-in QC tool in SMRT Link. As SMRT Link lacks a pre-built reference dataset for maize, command-line tools from the PacBio pbbioconda suite (github.com/PacificBiosciences/pbbioconda) were used for subsequent data processing steps.

First, raw concatenated HiFi reads were processed with SKERA (v.0.1.0)^72^ to trim MAS-Seq adapters, reads were split into S-reads and primers were removed using lima (v.2.6.33)^73^. The S-reads were then further processed with IsoSeq3 (v.3.8.1)^74^ to filter out cDNA reads lacking a barcode and/or bearing undesirable primer combinations, followed by poly-A tail trimming and deduplication, yielding full-length non-concatemer (FLNC) reads. The FLNC reads were subsequently aligned to the latest *Z. mays* B73 version 5.0 reference genome (retrieved from ncbi.nlm.nih.gov/datasets/genome/GCF_902167145.1, accessed in October 2023)^75^. Aligned FLNC reads were clustered into unique isoforms using IsoSeq3’s collapse function and then classified using Pigeon (v.1.0.0)^76^. Isoform-level count matrix files, suitable for import into Seurat, were generated using Pigeon’s make-seurat function.

### Computational annotations of novel isoforms via protein-coding potential, functional domains, poly-A motifs and ACRs

Novel isoforms in this study were classified into three categories: NNC, NIC and ISM (Supplementary Table 2). To evaluate their coding potential, we first identified the ORFs for each isoform sequence using a stand-alone version of ORF-finder^77^ and translated them into amino acid sequences using the SeqKit toolkit^78^.

Protein-coding potential was assessed using three independent algorithms: CPAT (v.1.2.1) with the plant model (threshold, 0.46)^28,29^, LncFinder (v.1.1.6)^30^ and PlantLncBoost^29^, the latter two using the default parameters. Final coding potential was determined on the basis of a majority consensus of these three tools. Functional domains in the novel isoforms were identified by scanning predicted amino acid sequences against the PFAM database.

To examine isoforms with novel 3′ exons, we performed pattern matching on the 3′-exon sequences to identify the two canonical poly-A motifs (AATAAA and ATTAAA). Additionally, to provide regulatory support for isoforms with alternative TSSs—specifically, those located upstream of the canonical TSS—we integrated published ATAC-seq datasets from six maize tissues. These data were used to determine whether these alternative TSSs were associated with distinct, proximal ACRs^33^.

### Spatial transcriptome normalization, dimension reduction and unsupervised clustering

The isoform-level count matrix generated from the MAS-IsoSeq data was imported into a Seurat object using the ReadMtx function in the R package Seurat (v.5.0.1)^79^. The dataset was normalized using the NormalizeData function, and highly variable isoforms were identified using FindVariableFeatures with 2,000 features and the vst selection method. After we scaled the data with the ScaleData function, we performed principal component analysis, retaining 50 principal components using the RunPCA function. We observed a potential clustering bias among different tissue sections on the same slide, probably stemming from variations in tissue preparation. To mitigate this batch effect, we applied batch correction using the Harmony package (v.1.0.1)^80^, considering individual sections as covariates. The batch-corrected reduction was then used as input for the RunUMAP function to perform UMAP dimension reduction, using the first 30 principal components. Unsupervised clustering of the spatial spots was achieved using the shared nearest neighbour modularity optimization-based clustering algorithm implemented in the FindClusters function (resolution, 1.0). To identify cluster-specific marker isoforms, the FindAllMarkers function was used, and markers with an adjusted *P* value (using Bonferroni correction) less than 0.05 were retained. Spatial image information and barcoded-spot locations were integrated into the Seurat object using the STutility package (v.1.1.1)^81^ for visualization.

### Isoform co-expression network construction and module functional annotation

Isoform co-expression network analysis was performed using the R package hdWGCNA (v.0.3.01)^42^. To address data sparsity during network construction while preserving cellular heterogeneity within unsupervised clusters, spatial spots from each cluster were aggregated into ‘metacells’, defined as groups of spatial spots with similar gene expression profiles. Network construction used auto-detection for optimal soft-thresholding power. A lower detectCutHeight parameter (0.980) in the dendrogram was applied for sensitive module detection. For each module, the top 25 hub isoforms were identified and subjected to GO enrichment analysis using the R package g:profiler2 (v.0.2.3)^82^.

### Pseudotime trajectory analysis

Pseudotime trajectory analysis was performed using the R package Monocle 3 (v.1.3.4)^43^. The Seurat object from this study was directly converted into cell dataset that is compatible with the input for Monocle 3 using the as.cell_data_set function. The learn_graph function was deployed to construct the principal graph from the reduced dimension (UMAP coordinates in this case). Pseudotime values were then assigned to each spatial cell barcode by determining the trajectory ‘root’ using the order_cells function. The root of the trajectory was specifically set at the node corresponding to the SAM_P1 tissue domain cluster. The differentiation states of each tissue domain cluster and the root node state were independently verified using the CytoTRACE package (v.0.3.3)^83^. CytoTRACE is used to identify the root cluster for pseudotime analysis by assigning a differentiation score or CytoTRACE score to each tissue cluster. The score is calculated on the basis of the number of unique genes expressed and overall gene counts within the tissue clusters, normalized to a range from 0 to 1, with 1 being the least differentiated.

### Construction of the MaizeV5_IsoAnn gene-model database

The new maize isoform-annotated gene-model database, named MaizeV5_IsoAnn, was constructed through a comprehensive integration of specific isoform categories derived from our long-read sequencing data. These categories included isoforms with novel exons, isoforms with extended exons on the 5′- and/or 3′-flanking regions and isoforms representing entirely unannotated (novel) genes. The process commenced by comparing the LRiso gene models to the B73 RefGen_v5 reference gene models^75^ using GffCompare (v.0.12.6)^84^. Isoforms with transcript classification codes of ‘k’ (novel isoforms whose gene body contains a reference gene body) and ‘u’ (unknown or intergenic isoforms) were extracted. Crucially, any novel isoforms overlapping with existing annotated genes were manually filtered out to ensure annotation specificity. The resulting GFF file of these extended gene models, incorporating selected novel isoforms, was then sorted and reformatted using the agat_convert_sp_gxf2gxf function from Another GTF/GFF Analysis Toolkit (v.1.0)^85^.

To rigorously filter the 6,878 putative novel genes initially inferred in intergenic regions, a set of four stringent criteria were implemented. A novel gene was included in the MaizeV5_IsoAnn gene-model database if it met at least one of the following criteria: (1) protein homology (BLASTP hits (*E*-value ≤ 1 × 10^−5^) against the NCBI non-redundant protein database), (2) expression support (expression level ≥1 TPM in at least 6 of the 78 LCM-captured embryonic leaf cell RNA-seq datasets^19^), (3) protein domain prediction (InterProScan hits (*E*-value ≤ 1 × 10^−5^) on at least one isoform) and (4) independent annotation (support from EviAnn annotation^86^).

### Functional annotation and protein-coding potential of novel genes

To characterize the novel genes, nucleotide sequences were first translated into amino acid sequences using ORF-finder^77^. Local BLASTP searches were performed against the NCBI non-redundant protein database (v.5, accessed in March 2024), with the search space restricted to the Viridiplantae taxonomic clade. Simultaneously, protein domains and functional signatures were identified using InterProScan searches conducted locally against all available InterPro member databases. To further validate the novel gene models, we used EviAnn^86^ to identify high-confidence protein-coding regions, using FLNC transcript sequences from the IsoSeq3 output, the maize B73 reference genome and reference protein sequences as inputs. Novel genes annotated by EviAnn were categorized as strongly supported gene models. Finally, the protein-coding or noncoding potential of each novel gene was predicted using a consensus approach involving CPAT^28^ (plant model), LncFinder^30^ and PlantLncBoost^29^. To identify lncRNA among the novel transcripts, we performed a local BLASTn search using all isoforms from both novel and known genes against the *Z. mays* lncRNA reference. The lncRNA reference sequences (Ensembl Plants 51) were retrieved from the GreeNC database (v.2.0, accessed in April 2026)^50^ to construct the local BLAST database using the makeblastdb command. We applied stringent alignment criteria, requiring a query coverage ≥90%, sequence identity ≥90% and *E*-value ≤ 1 × 10^−5^.

### Short-read RNA-seq library mapping and TO-GCN analysis

Bulk RNA-seq datasets from previously published LCM samples were retrieved from the NCBI SRA (accession number: PRJNA831613). The quality of these short-read RNA-seq datasets was assessed using fastp (v.0.23.4)^85^. Reads were then mapped to the B73 v.5.0 reference genome (GCF_902167145.1) using the STAR aligner (v.2.7.10b)^87^. Mapped reads for each gene were quantified using HTseq-count (v.2.0.2)^88^ and normalized to TPM. TO-GCN (v.1.0)^25^ analysis was conducted following the established TO-GCN pipeline. A comprehensive list of *Z. mays* TFs was obtained from the Plant Transcription Factor Database v.5.0 (planttfdb.gao-lab.org, accessed February 2024)^51^. TO-GCN analysis outputs were visualized using Cytoscape (v.3.10.1)^89^. Functional enrichment analysis was performed using the g:profiler2 package (v.0.2.3)^82^ to identify enriched GO biological process terms for co-expressed non-TF genes within each level of the TO-GCN.

### Spatial localization of isoforms via padlock probe-based RCA

Target genes containing both canonical and novel isoforms were selected on the basis of their expression levels in the long-read dataset. Padlock probes were designed as 100-nucleotide oligonucleotides comprising three distinct functional domains: a symmetric 15-nucleotide target-complementary arm at both the 5′ and 3′ termini, a 20-nucleotide oligonucleotide detection binding site and a 31-nucleotide central backbone containing the primer-binding domain. The target-complementary arms were specifically designed to span the unique splice junction or the target exon exclusive to the individual isoform transcript (Supplementary Table 24). To ensure the probe specificity, a BLASTn search was performed for each oligonucleotide sequence. Probes were selected only if the top five hits with an *E*-value < 1 × 10^−5^ matched exclusively to the target gene.

RCA-based transcript detection was performed on 12-µm fixed tissue sections mounted on glass slides and processed using SecureSeal reaction chambers (Sigma-Aldrich). Slides were equilibrated to room temperature for 10 min and washed with DEPC-treated PBS. Sections were permeabilized with proteinase K (20 µg ml^−1^ at 37 °C; duration empirically optimized for 5–10 min) and quickly rinsed in PBS. For target hybridization, sections were incubated with 50 µl of hybridization mix containing 1× SplintR buffer, RNase inhibitor (1 U µl^−1^), BSA (0.2 µg µl^−1^), 10% formamide and 0.5 µM of each padlock probe. After a brief denaturation and annealing step (48 °C for 15 min, followed by 4 °C for 5 min), SplintR ligation was performed by adding SplintR ligase (final activity ~1 U µl^−1^) and incubating at 37 °C for 3 h.

Following two washes in DEPC-PBS, RCA was initiated by adding 50 µl of RCA mix containing Phi29 DNA polymerase (1 U µl^−1^), 1× Phi29 buffer, 0.25 mM dNTPs, 4 mM DTT, 0.2 µg µl^−1^ BSA, 1 U µl^−1^ RNase inhibitor and 0.1 µM RCA initiation primer (0.1 µM, 5′-AGTCCCAGTGTACCCACAAAC-3′). The chambers were sealed and incubated at 37 °C for 2 h, followed by overnight incubation at 30 °C. After being washed with DEPC-PBS, RCA products were visualized using hybridizing fluorescent detection oligonucleotides (0.1 µM each) in a 2× hybridization buffer (2× SSC, 20% formamide) for 30 min at room temperature. Finally, the sections were washed twice with DEPC-PBS containing 0.05% Tween-20, air-dried and mounted with antifade medium for fluorescence imaging.

### TF binding motif analysis

TF binding sites in the promoter regions (spanning from 1,000 bp upstream to 100 bp downstream of its TSS according to PlantPAN4.0) and their corresponding cognate TFs were identified using data from the PlantPAN4.0 database (accessed in November 2024)^55^. CNS analysis was also conducted using tools available within PlantPAN4.0.

### Reanalysis of public ATAC-seq data on the *Z. mays* v.5 reference genome

Publicly available ATAC-seq datasets from six maize tissues (silk, SAM, pericarp, endosperm, stem and embryo) were retrieved from the NCBI SRA (PRJNA1048297)^33^. Raw reads were trimmed for adapter sequences and low-quality bases using Trimmomatic (v.0.39)^90^. The resulting clean reads were aligned to the maize B73 version 5.0 reference genome (GCF_902167145.1) using Bowtie2 (v.2.3.4.1)^91^ with the default parameters. Post-alignment, PCR duplicates and mitochondrial reads were removed, and only uniquely mapped reads were retained for downstream analysis.

Chromatin accessibility peaks were called independently for each sample using MACS2 (v.2.2.7.1)^58^ with parameters optimized for ATAC-seq (nomodel; shift, −100; extsize, 200). For peak annotation, accessible genomic regions were assigned to gene-associated promoter regions, defined as the ±1-kb window flanking the TSS. These promoter regions were delineated on the basis of the extended 5′ UTRs of both currently annotated genes and the novel genes identified in this study. Peaks overlapping these defined regions were assigned to their respective genes for further integrative analysis.

### DAP-seq analysis and inference of the binding motif of *LDB26*

The *Zm*LBD26 coding sequence was amplified from three-day-old maize seedling RNA using the primers ZmLBD26-F (5′-ATGAGGGACGCGGTGGTG-3′) and ZmLBD26-R (5′-CTAGTAGGACGACCAGAAGTGG-3′). The coding sequence was cloned into pCR8/GW/TOPO and recombined into pIX-Halo. Halo-tagged *Zm*LBD26 and GST (negative control) proteins were synthesized using 2 µg of plasmid in the TnT T7 Coupled Wheat Germ Extract System. Simultaneously, genomic DNA from seven-day-old *S. italica* leaves was fragmented to 200 bp using a Covaris M220 Ultrasonicator and prepared as a shotgun library using the NEBNext Ultra II DNA Library Prep Kit with double-sided size selection.

For protein–DNA binding, synthesized proteins were immobilized on Magne HaloTag Beads and incubated with 300 ng of the millet library at 30 °C for 90 min. Bound DNA for both *Zm*LBD26 and the GST control was eluted at 95 °C, PCR-indexed for 13 cycles using NEBNext UDI Oligos and purified using a 1:1 magnetic bead ratio. Final libraries were sequenced on an Illumina NextSeq 2000 platform (paired-end 151 bp) at the High Throughput Genomics Core at Academia Sinica, Taiwan. Raw reads were processed using Trimmomatic (v.0.40)^90^ to remove adapters and reads ≤50 bp. High-quality reads were aligned to the *S. italica* genome (v.2.0)^92^ using BWA-MEM (v.0.7.19)^93^. Peak calling was performed using MACS3 (v.3.0.4)^58^ with the call-summits option. To minimize background noise, peaks were filtered against the GST-only control library. Overlapped peaks were clustered, and the top 500 were selected by summation score^94^ for motif identification using MEME-ChIP (v.5.5.9)^95^ within a ±100-bp window. Peak annotation was performed using ChIPseeker (v.1.38.0)^96^, and potential binding sites in the MaizeV5_IsoAnn database were scanned using FIMO (v.5.5.9)^59^.

### Reporting summary

Further information on research design is available in the Nature Portfolio Reporting Summary linked to this article.

## Supplementary information

Supplementary Information Supplementary Figs. 1–10 and Tables 1–3, 8–10, 12, 15, 19, 20, 23 and 24.

Reporting Summary

Peer Review file

Supplementary Table 4 Novel isoforms with computational support. The support includes poly-A motif analysis, PFAM hits, protein-coding probability analysis and ATAC-seq analysis.

Supplementary Table 5 Metrics of the Seurat metadata for each barcoded spatial spot on the 10x Genomics Visium slide.

Supplementary Table 6 Cluster-specific markers from isoform-level unsupervised clustering. The clusters are labelled with putative tissue annotations.

Supplementary Table 7 Cluster-specific markers from gene-level unsupervised clustering. The clusters are labelled with putative tissue annotations.

Supplementary Table 11 Assignment of co-expression modules for each expressed isoform from WGCNA analysis.

Supplementary Table 13 Extended gene models included in the construction of the MaizeV5_IsoAnn gene-model database. The distance comparison of start (Start_diff) and end (End_diff) sites is also shown.

Supplementary Table 14 Novel gene models integrated into the MaizeV5_IsoAnn database. Candidate genes were selected on the basis of high-stringency filtering criteria, including protein homology (BLASTP), conserved domain identification (PFAMscan) and minimum transcriptional expression thresholds (TPM).

Supplementary Table 16 PFAM hits and protein-coding potential of the novel genes.

Supplementary Table 17 Comparative mapping statistics between the B73 RefGen_v5 and the MaizeV5_IsoAnn gene-model databases for each LCM-captured RNA-seq dataset.

Supplementary Table 18 TO-GCN analyses of transcription factors. TF genes were assigned to different TO-GCN levels using MaizeV5_IsoAnn gene models.

Supplementary Table 21 Target genes with putative LBD26 protein-binding motifs in the promoter regions (±1,000 bp from the TSS).

Supplementary Table 22 Genes putatively regulated by LBD26.

## Data Availability

The long-read transcript sequence dataset from MAS-IsoSeq and DAP-seq can be downloaded from the NCBI SRA with the accession number PRJNA1214004. The short-read 10x Genomics spatial transcriptomics dataset can be retrieved from the SRA and Gene Expression Omnibus repositories using the accession numbers SRR17981909 (VR03) and SRR17968079 (VR01). Related images and processed spatial transcriptomics datasets have been deposited in Gene Expression Omnibus repositories using the accession numbers GSM5903593 (VR03) and GSM5903595 (VR01). The MaizeV5_IsoAnn gene-model database, EviAnn annotated gene models and high-resolution histology images are available via Zenodo at https://doi.org/10.5281/zenodo.16837070 (ref. ^97^), https://doi.org/10.5281/zenodo.16868057 (ref. ^98^) and https://doi.org/10.5281/zenodo.20741009 (ref. ^99^), respectively.

## Code availability

The source code for all the analyses used in this study is publicly available via GitHub at https://github.com/danielylup/Maize_SpMASIsoSeq.
