# Supplementary material for: Profiling maize embryonic leaf development and discovering new genes using high-resolution spatial long-read isoform sequencing
Source: Nat Plants. 2026 Aug 17;12(9):1860–78. doi: 10.1038/s41477-026-02364-y (PMC13585551; doi:10.1038/s41477-026-02364-y)
Supplement: Supplementary file 1 [file 41477_2026_2364_MOESM1_ESM.pdf]

nature plants

Supplementary information

https://doi.org/10.1038/s41477-026-02364-y

Profiling maize embryonic leaf development and discovering new genes using high-resolution spatial long-read isoform sequencing

In the format provided by the authors and unedited

Supplementary Figures

Supplementary Fig. 1 | UMAP projections and spatial distribution of maize embryonic shoot tissue.

Supplementary Fig. 2 | Heatmap of top 10 marker isoforms across annotated tissue clusters.

Supplementary Fig. 3 | Spatial localization and expression profiles of specific isoforms.

Supplementary Fig. 4 | Comparative analysis and isoform dynamics of vein tissue domains.

Supplementary Fig. 5 | Modular distribution of gene isoforms and co-expression dynamics.

Supplementary Fig. 6 | Estimation of tissue differentiation states using CytoTRACE.

Supplementary Fig. 7 | Accessible Chromatin Regions (ACRs) at the promoter region of novel genes.

Supplementary Fig. 8 | Principal component analysis (PCA) plots of transcriptional profiles in different LCM-isolated cell types.

Supplementary Fig. 9 | Size distribution of MAS-IsoSeq cDNA libraries.

Supplementary Fig. 10 | Quality check metrics of the VR03 datasets from three separate MAS-IsoSeq runs.

Supplementary Tables

Supplementary Table 1. Sequencing metrics and quality check of MAS-IsoSeq using PacBio HiFi Sequel IIe.

Supplementary Table 2. Nomenclature of the terms used in the study.

Supplementary Table 3. Comparison of 5'- and 3'-end locations between B73 RefGen_v5 and LRiso gene models.

Supplementary Table 4. Novel isoforms with computational supports.

Supplementary Table 5. Metrics of the Seurat metadata for each barcoded spatial spot on the 10x Genomics Visium slide.

Supplementary Table 6. Cluster-specific markers from isoform-level unsupervised clustering.

Supplementary Table 7. Cluster-specific markers from gene-level unsupervised clustering.

Supplementary Table 8. Intersection of up-regulated isoforms across vein tissue clusters.

Supplementary Table 9. Marker isoforms in the embry_v1 cluster.

Supplementary Table 10. Marker isoforms in the embry_v2 cluster.

Supplementary Table 11. Assignment of co-expression modules for each expressed isoform from WGCNA analysis.

Supplementary Table 12. Enriched biological process GO terms for the hub isoforms of each module.

Supplementary Table 13. Extended gene models included in the construction of the MaizeV5_IsoAnn gene-model database.

Supplementary Table 14. Novel gene models integrated into the MaizeV5_IsoAnn database.

Supplementary Table 15. Evaluation and categorization of novel gene models.

Supplementary Table 16. PFAM hits and protein-coding potential of the novel genes.

Supplementary Table 17. Comparative mapping statistics between the B73 RefGen_v5 and the MaizeV5_IsoAnn gene-model database for each LCM-captured RNA-seq dataset.

Supplementary Table 18. TO-GCN analyses of transcription factors.

Supplementary Table 19. Distribution of transcription factor (TF) genes across TO-GCN levels in the B73 RefGen_v5 and MaizeV5_IsoAnn databases.

Supplementary Table 20. GO enrichment of biological processes for non-TF genes co-expressed with transcription factors.

Supplementary Table 21. Target genes with putative LBD26 protein binding motif in the promoter region.

Supplementary Table 22. Genes putatively regulated by LBD26 protein.

Supplementary Table 23. Marker genes from a single-cell resolution spatial platform were compared with marker isoforms revealed in this study.

Supplementary Table 24. Probes used for padlock-probe rolling circle amplification (RCA) *in situ* hybridization.

Supplementary Figures

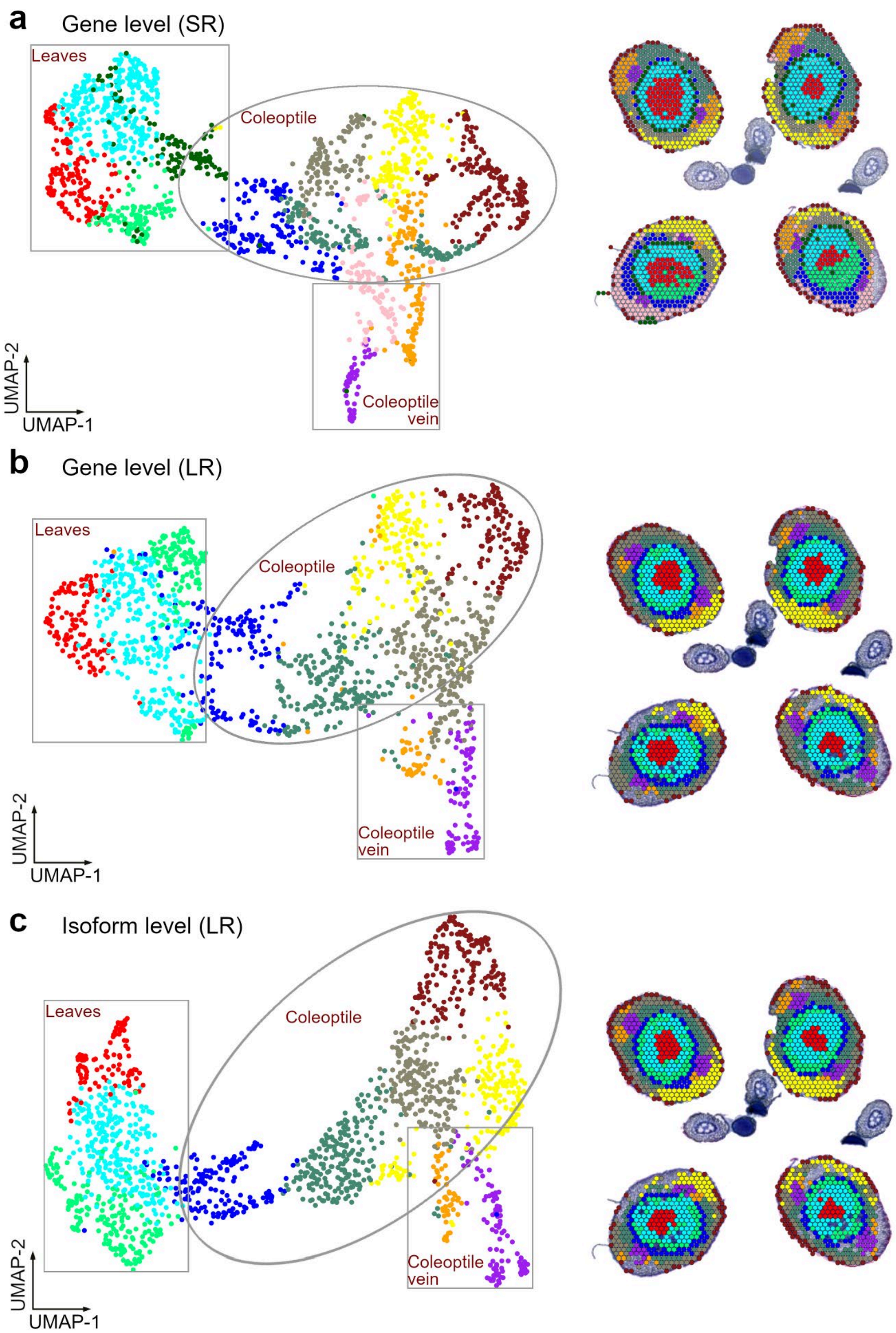

Supplementary Fig. 1 | UMAP projections and spatial distribution of maize embryonic shoot tissues. a-c, UMAP plots (left) and corresponding spatial maps (right) generated from **(a)** gene-level short-read from the 10x Genomics dataset, **(b)** gene-level MAS-IsoSeq long-read data, and **(c)** isoform-level MAS-IsoSeq long-read data. Colors correspond to unsupervised clusters representing distinct tissue domains (excluding root tissue). In the UMAP

projections, clusters are delineated into “leaves”, “coleoptile”, and “coleoptile vein” domains (indicated by black boxes and ovals) based on histological morphology (right). Mapping was performed using the B73 RefGen_v5 reference for gene-level short reads and the LRiso gene model for both gene-level and isoform-level long-read datasets. The three UMAP plots show that the topological patterns are largely maintained between datasets.

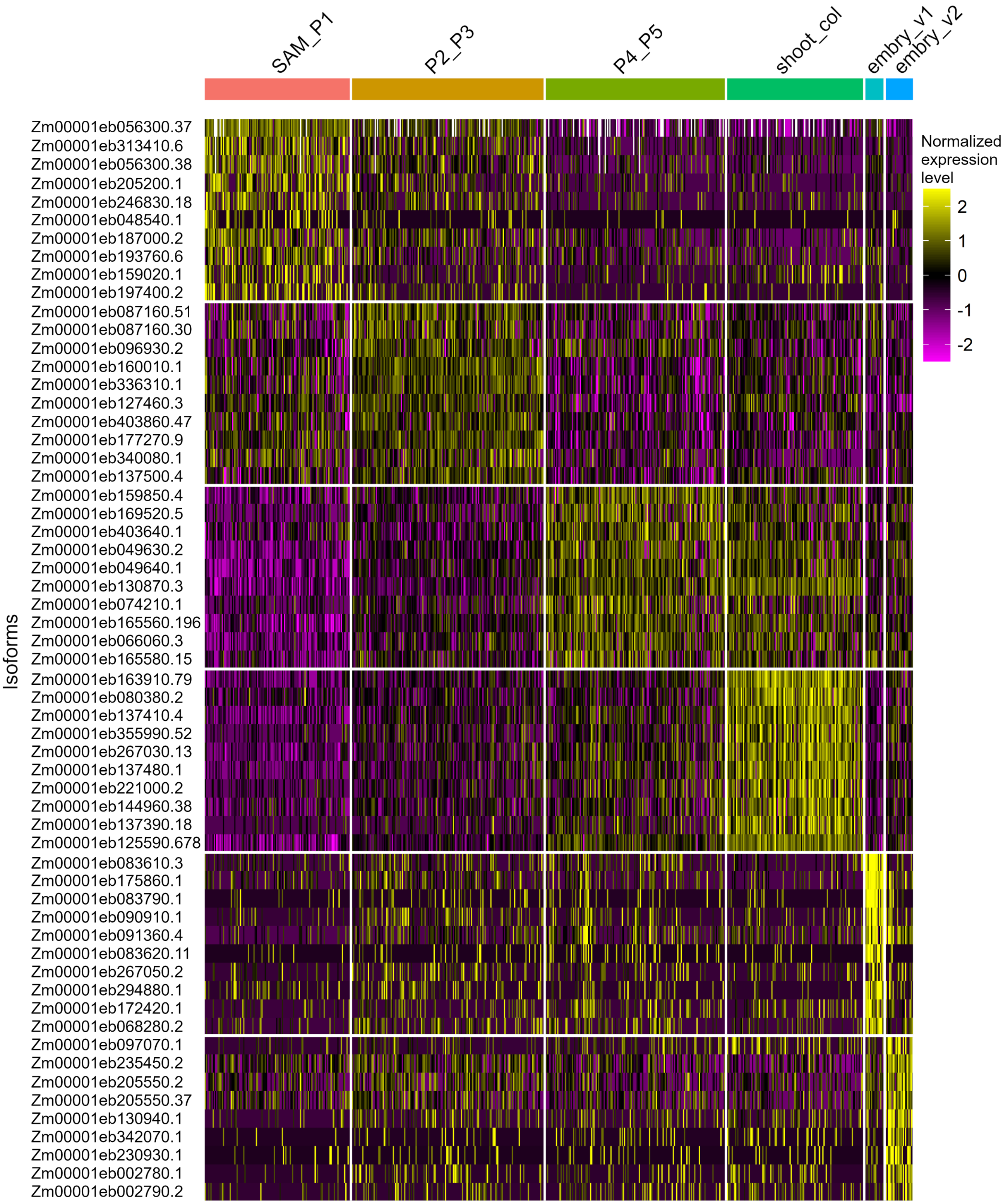

Supplementary Fig. 2 | Heatmap of top 10 marker isoforms across annotated tissue clusters. Each column represents individual spatial spots grouped by their respective tissue clusters. The color scale indicates normalized isoform expression levels (Z-scores). These marker isoforms

demonstrate high cluster specificity, effectively serving as markers for tissue clusters within the maize embryonic shoot.

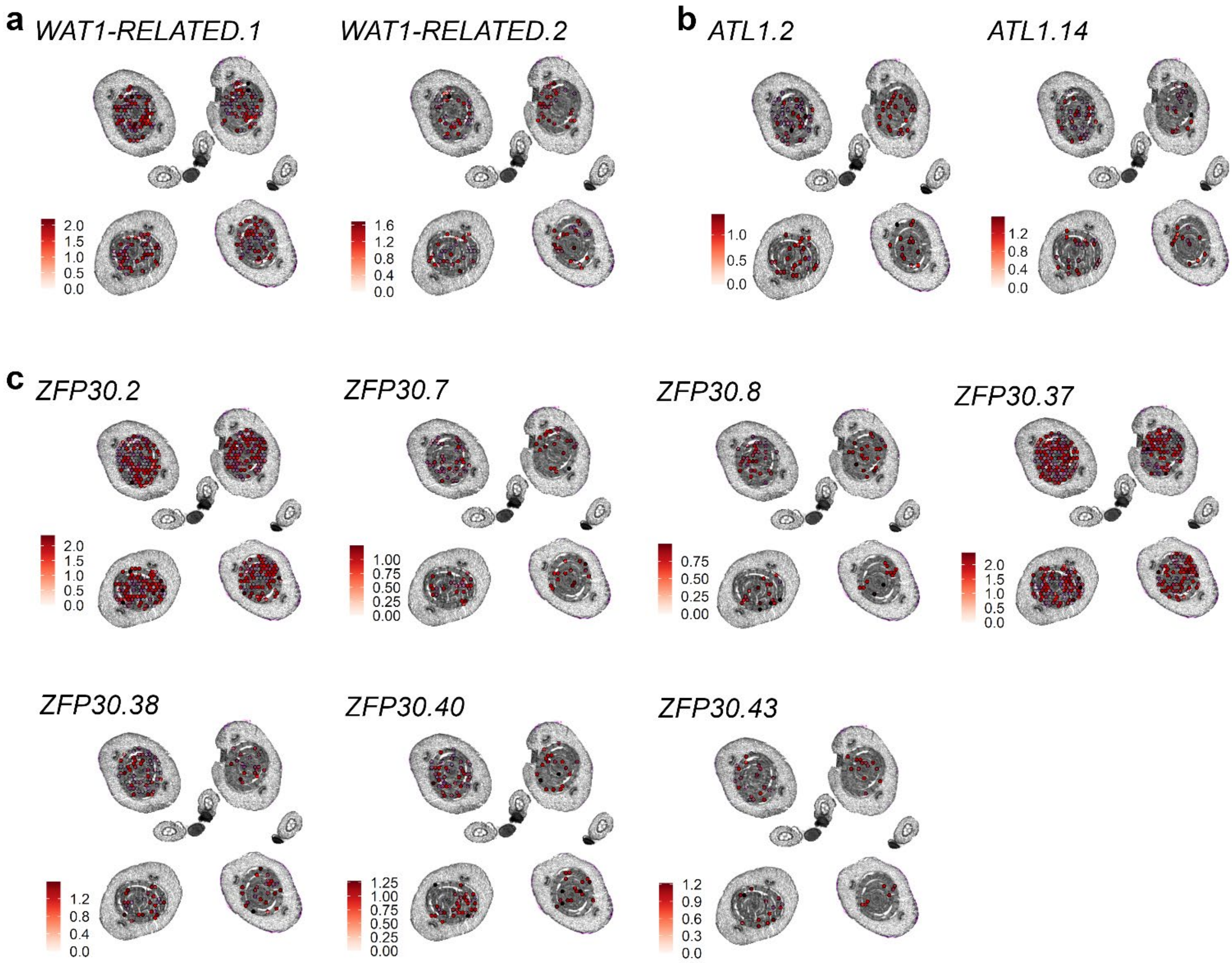

Supplementary Fig. 3 | Spatial localization and expression profiles of specific isoforms. Spatial distribution and normalized expression levels are shown for isoforms of **(a)** *WAT1-RELATED*, **(b)** *ATL1*, and **(c)** *ZFP30*. Each panel illustrates the localization of individual isoforms within the embryonic leaf tissues, with the color scale indicating normalized expression intensity.

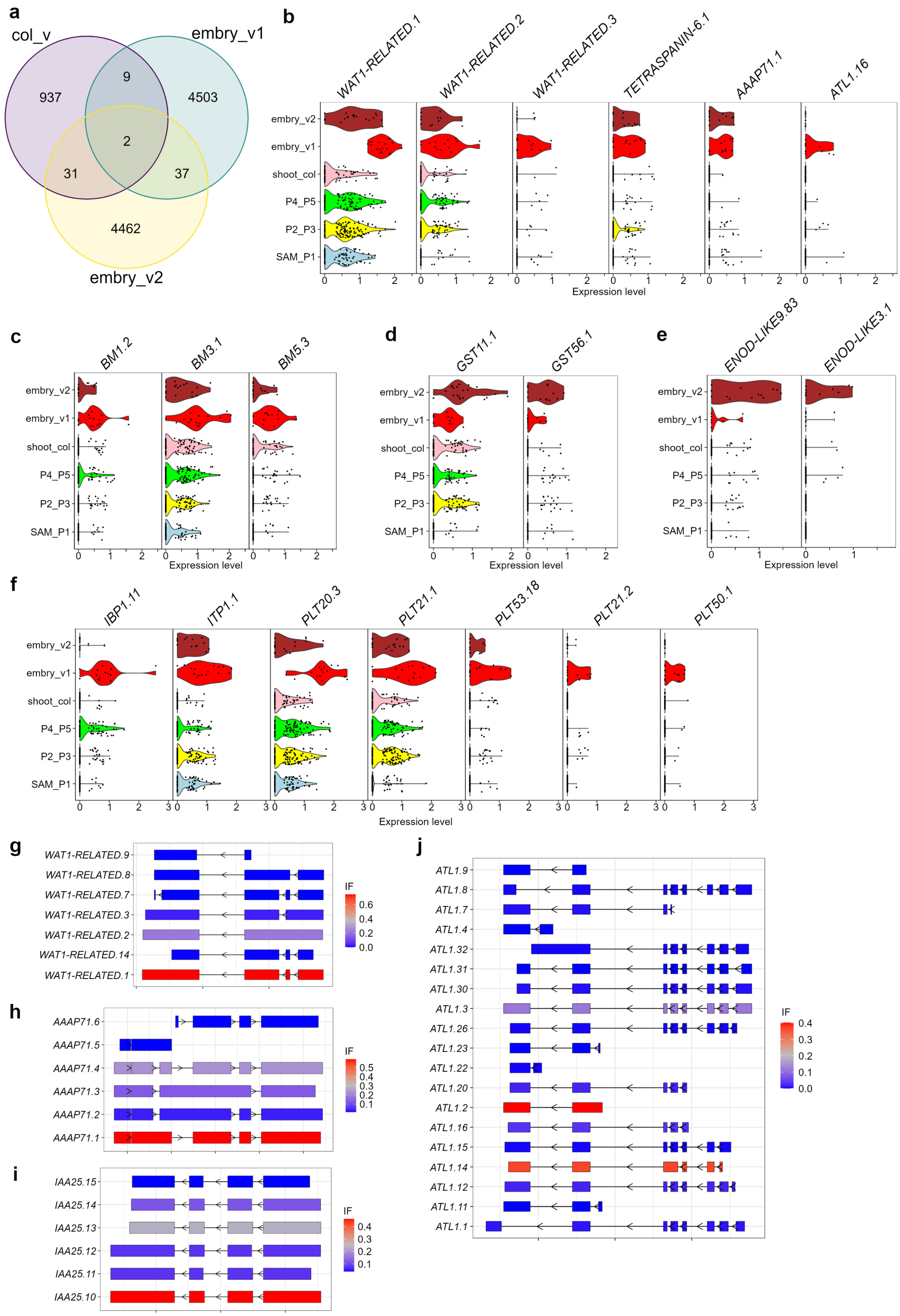
a
col_v
embry_v1
embry_v2
937
9
4503
2
31
37
4462
b
WAT1-RELATED.1
WAT1-RELATED.2
WAT1-RELATED.3
TETRASPANIN-6.1
AAAP71.1
ATL1.16
embry_v2
embry_v1
shoot_col
P4_P5
P2_P3
SAM_P1
Expression level
c
BM1.2
BM3.1
BM5.3
d
GST11.1
GST56.1
e
ENOD-LIKE9.83
ENOD-LIKE3.1
f
IBP1.11
ITP1.1
PLT20.3
PLT21.1
PLT53.18
PLT21.2
PLT50.1
g
WAT1-RELATED.9
WAT1-RELATED.8
WAT1-RELATED.7
WAT1-RELATED.3
WAT1-RELATED.2
WAT1-RELATED.14
WAT1-RELATED.1
IF
h
AAAP71.6
AAAP71.5
AAAP71.4
AAAP71.3
AAAP71.2
AAAP71.1
i
IAA25.15
IAA25.14
IAA25.13
IAA25.12
IAA25.11
IAA25.10
j
ATL1.9
ATL1.8
ATL1.7
ATL1.4
ATL1.32
ATL1.31
ATL1.30
ATL1.3
ATL1.26
ATL1.23
ATL1.22
ATL1.20
ATL1.2
ATL1.16
ATL1.15
ATL1.14
ATL1.12
ATL1.11
ATL1.1

Supplementary Fig. 4 | Comparative analysis and isoform dynamics of vein tissue domains. This analysis investigates the developmental progression of vascular tissues across the coleoptile vein (“col_v”) and two distinct stages of embryonic veins (“embry_v1” and “embry_v2”). **a,** Venn diagram illustrating the intersection of significantly up-regulated isoforms among the three vein tissue clusters. **b-f,** Expression profiles of characteristic marker isoforms for the embry_v1 and embry_v2 clusters across all tissue domains. These marker sets include isoforms associated with **(b)** auxin regulation and transport, **(c)** *BROWN MIDRID*, **(d)** *GLUTATHION S-TRANSFERASE,* **(e)** early nodulin-like *(ENODL)* proteins, and **(f)** lipid transfer. **g-j,** Isoform fraction (IF) distributions for the transcript variants of **(g)** *WAT1-RELATED,* **(h)** *AAAP71,* **(i)** *IAA25*, and **(j)** *ATL1*. IF levels are vidualized by a color gradient from dark blue (lowest) to bright red (highest). Arrows on individual isoform models indicate the transcriptional orientation (5’ to 3’).

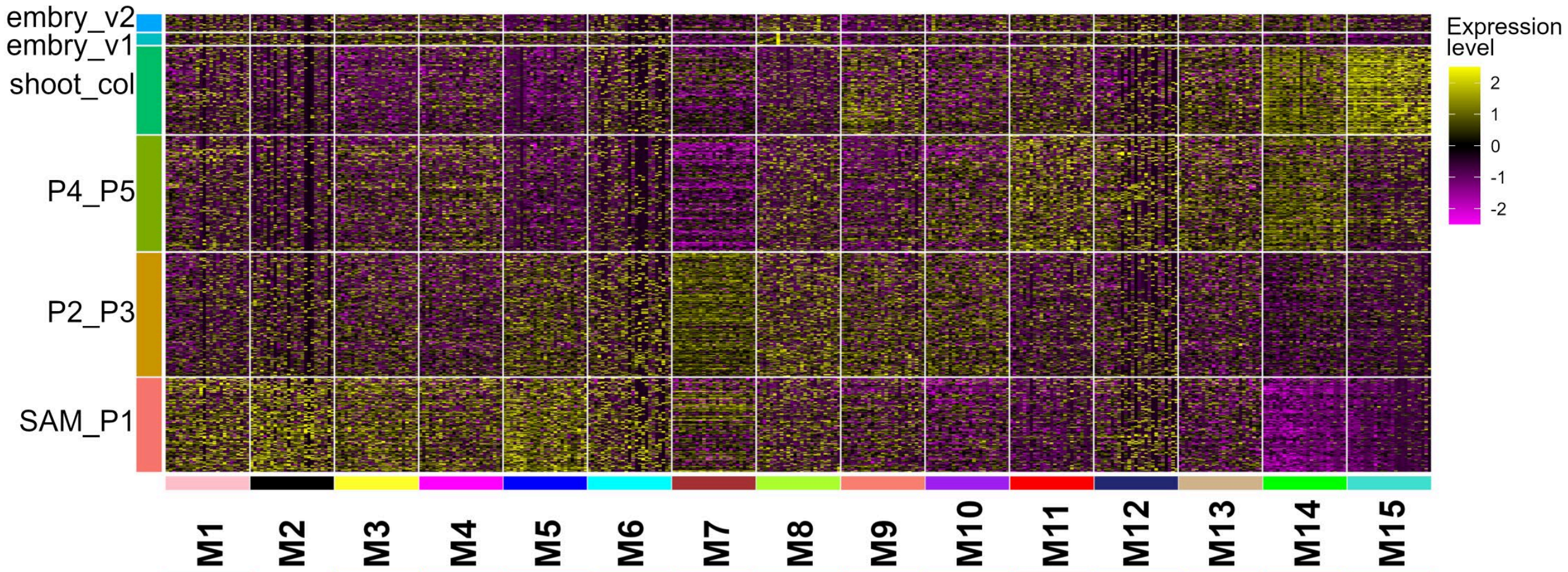

	M1	M2	M3	M4	M5	M6	M7	M8	M9	M10	M11	M12	M13	M14	M15
M1		23	38	29	19	12	18	7	2	5	15	5	4	4	12
M2			33	18	30	8	17	8	2	5	10	2	4	4	9
M3				27	38	3	23	7	1	4	12	5	7	6	12
M4					25	5	11	8	3	2	24	6	10	7	9
M5						2	21	9	0	3	17	3	2	1	9
M6							5	3	1	1	4	1	1	1	5
M7								13	4	9	9	4	5	3	20
M8									0	3	13	2	0	4	7
M9										4	3	0	1	5	9
M10											14	3	7	8	14
M11												9	15	44	23
M12													5	0	2
M13														20	6
M14															44
M15															

Supplementary Fig. 5 | Modular distribution of gene isoforms and co-expression dynamics. Top: Heatmap showing the expression profiles of the top 25 hub isoforms for each of the 15 co-expression modules. Columns represent hub isoforms and rows represent spatial spots grouped by tissue clusters. The color intensity reflects normalized expression, highlighting module-specific activity across domains. Bottom: Matrix table quantifying genes

with isoforms partitioned across co-expression modules. Values indicate the count of genes whose isoforms are assigned to specific module pairs (*x*- and *y*-axes). This highlights genes where alternative splicing produces multiple isoforms that participate in distinct regulatory networks (two or more modules).

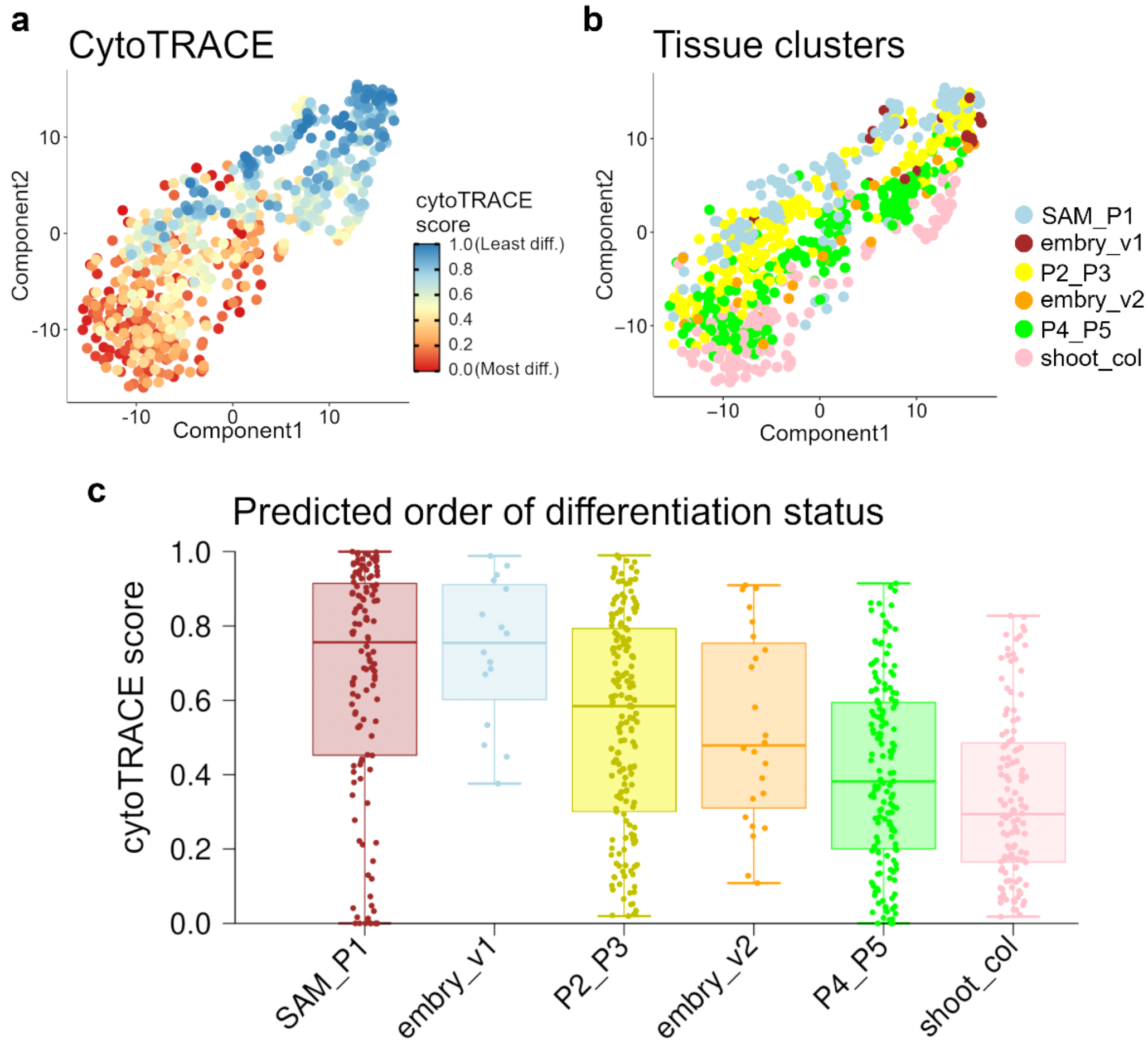

Supplementary Fig. 6 | Estimation of tissue differentiation states using CytoTRACE. CytoTRACE analysis was performed to identify the developmental "root" for pseudotime trajectories, assigning a differentiation score to each tissue cluster based on transcriptional diversity (unique gene counts). Higher CytoTRACE scores correspond to less differentiated states, representing the putative originating state of embryogenesis. **a,** UMAP projection color-coded by predicted differentiation states, ranging from 0 (most differentiated cells) to 1 (least differentiated cells). **b,** UMAP plot showing the distribution of the annotated tissue clusters. **c,** Boxplot of CytoTRACE scores by tissue cluster. Clusters are ordered along the *x*-axis from least differentiated (left) to most differentiated (right), providing a predicted developmental hierarchy.

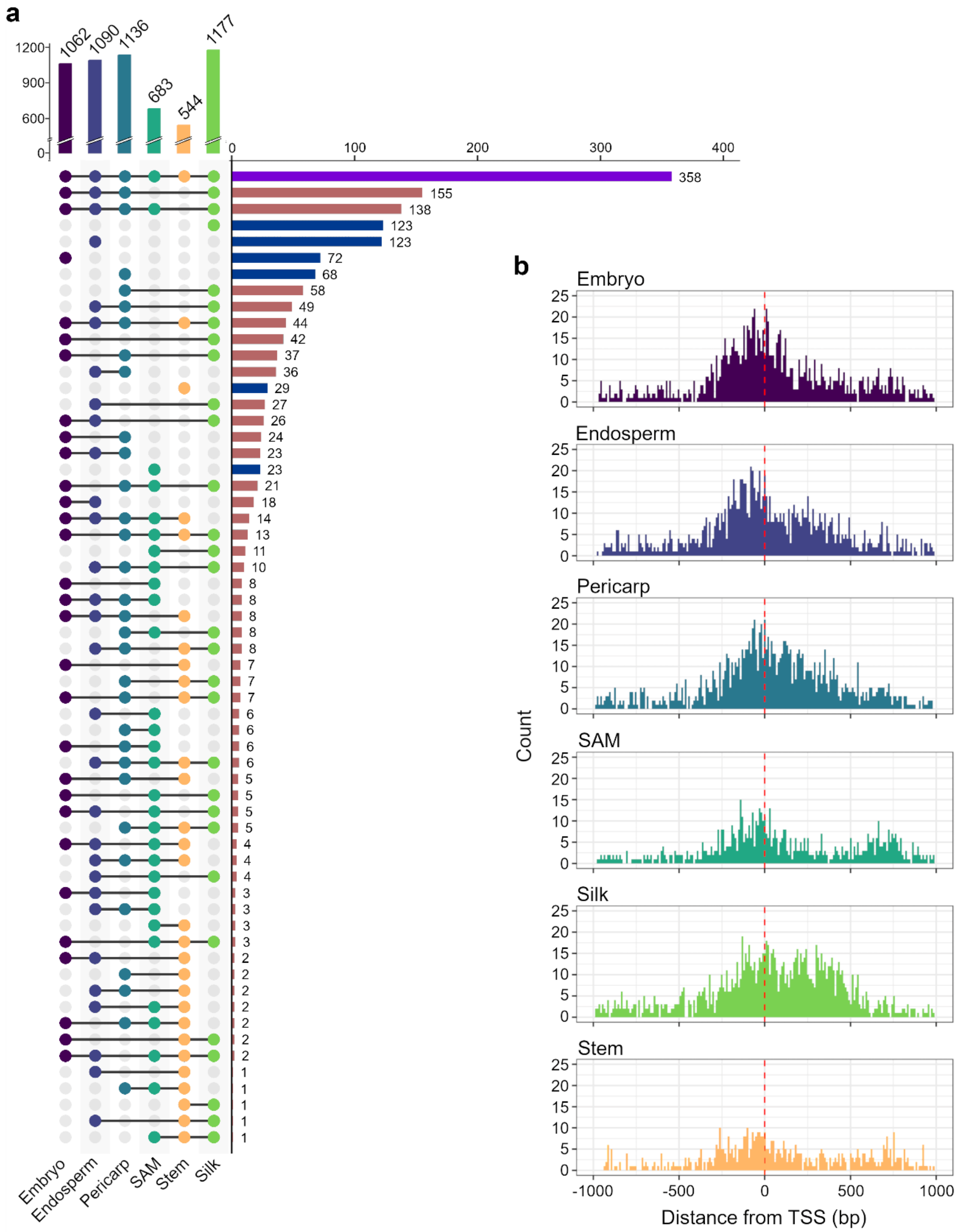

Supplementary Fig. 7 | Accessible Chromatin Regions (ACRs) at the promoter regions of novel genes. a, Top: Bar chart plot showing the total number of promoter-associated ACRs identified across six distinct tissue types (Liu et al., 2025). Bottom: UpSet plot illustrating the intersections of ACRs among the tissues. The horizontal bars indicate the distribution of accessibility: the dark purple bar highlights 358 novel genes exhibiting constitutive chromatin accessibility across all six tissue types; light purple bars represent accessibility across multiple (but not all) tissues; and blue bars indicate novel genes with

tissue-specific accessibility. **b,** Genomic distribution of ACR peaks relative to the TSS (red dashed line) for each of the six tissues, demonstrating significant enrichment immediately upstream of the TSS.

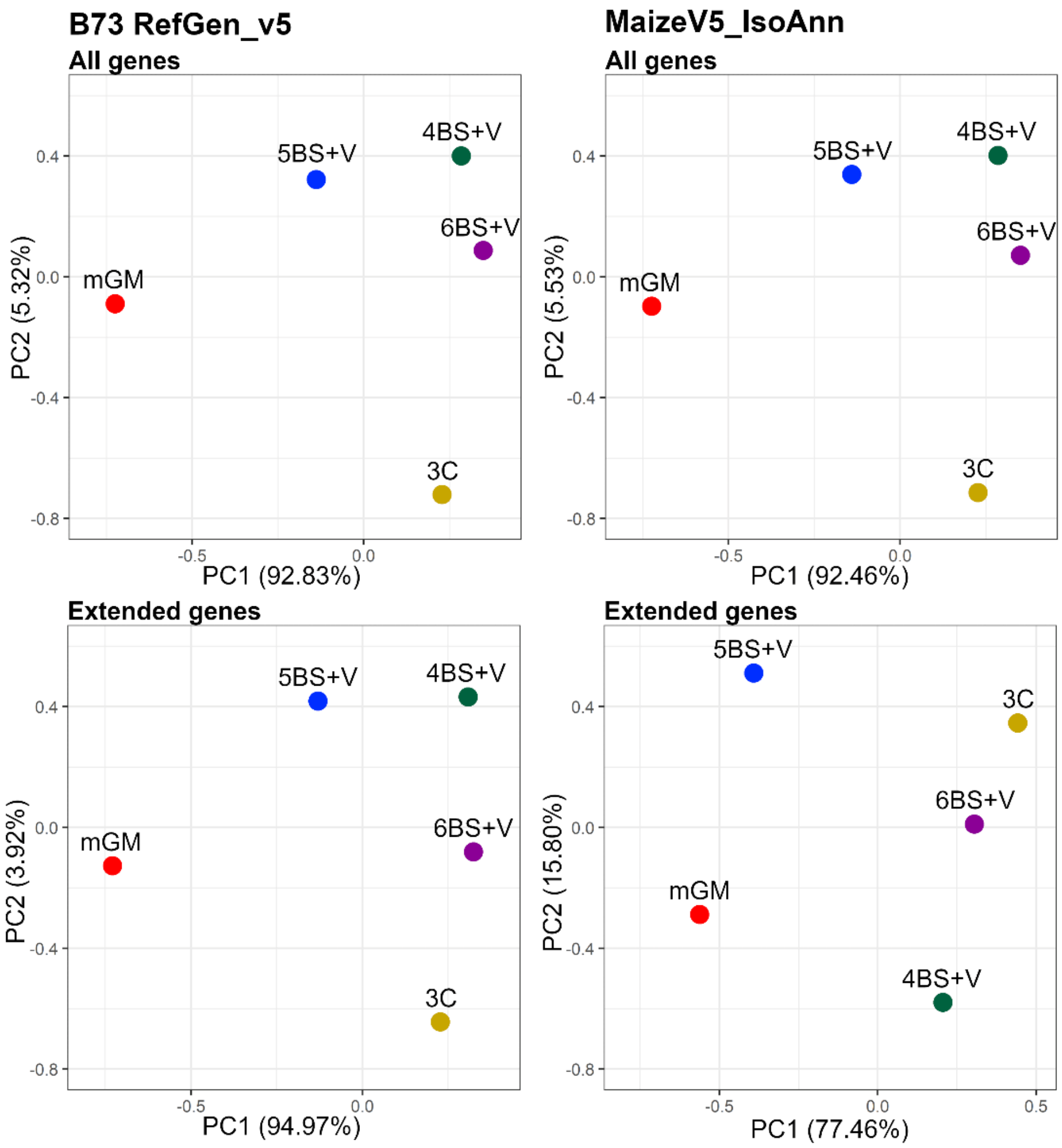

Supplementary Fig. 8 | Principal component analysis (PCA) plots of transcriptional profiles in different LCM-isolated cell types. The figures compare the PCA clustering based on the B73 RefGen_v5 gene-model database (44,304 genes; left) with the MaizeV5_IsoAnn gene-model database (49,630 genes; right). The top panel shows clustering of “all gene models”, and the bottom panel shows only the 1,678 “extended gene models”.

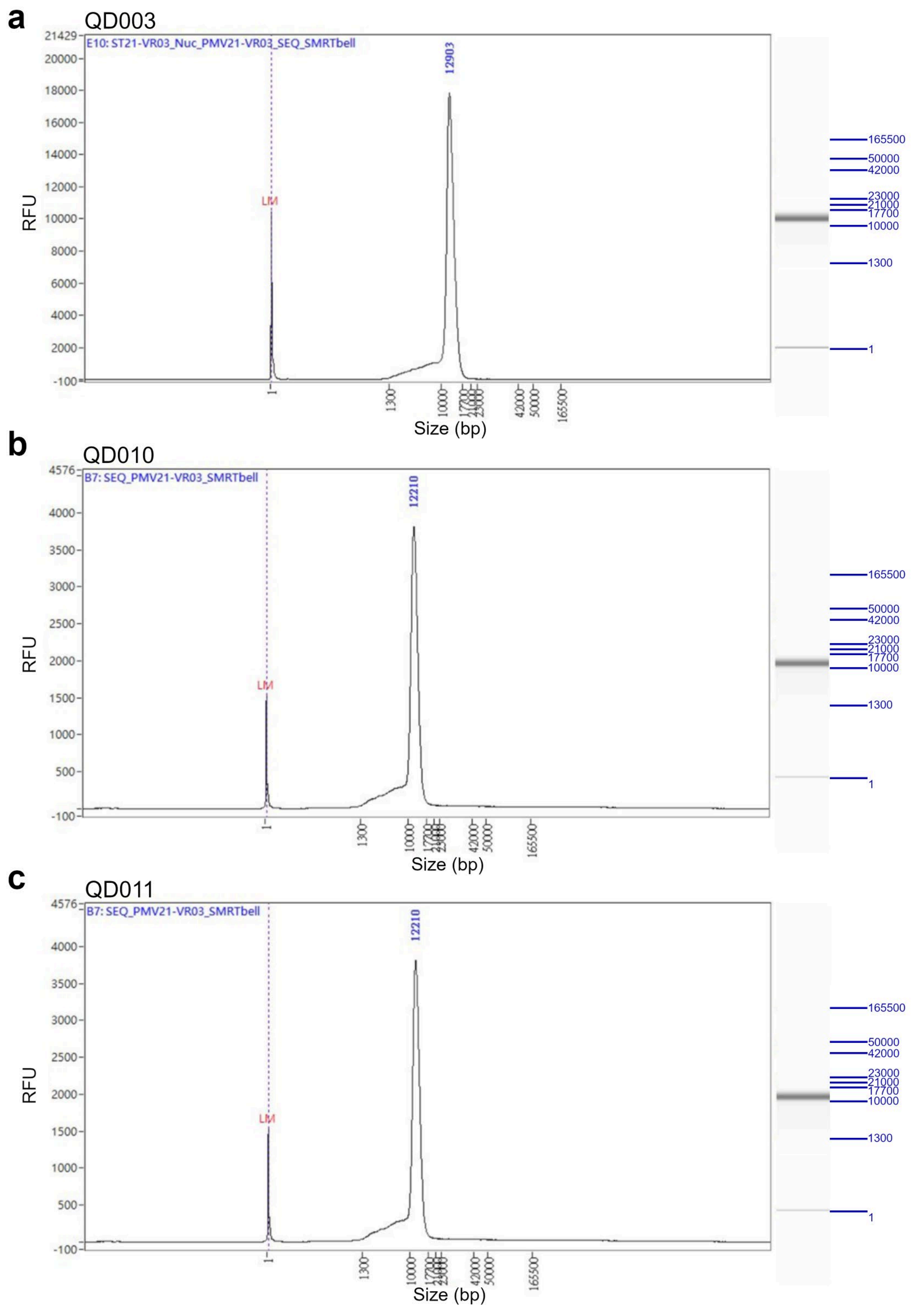

Supplementary Fig. 9 | Size distribution of MAS-IsoSeq cDNA libraries. Fragment Analyzer traces illustrate the cDNA profiles for three independent libraries used to generate datasets (a) QD003, (b) QD010, and (c) QD011.

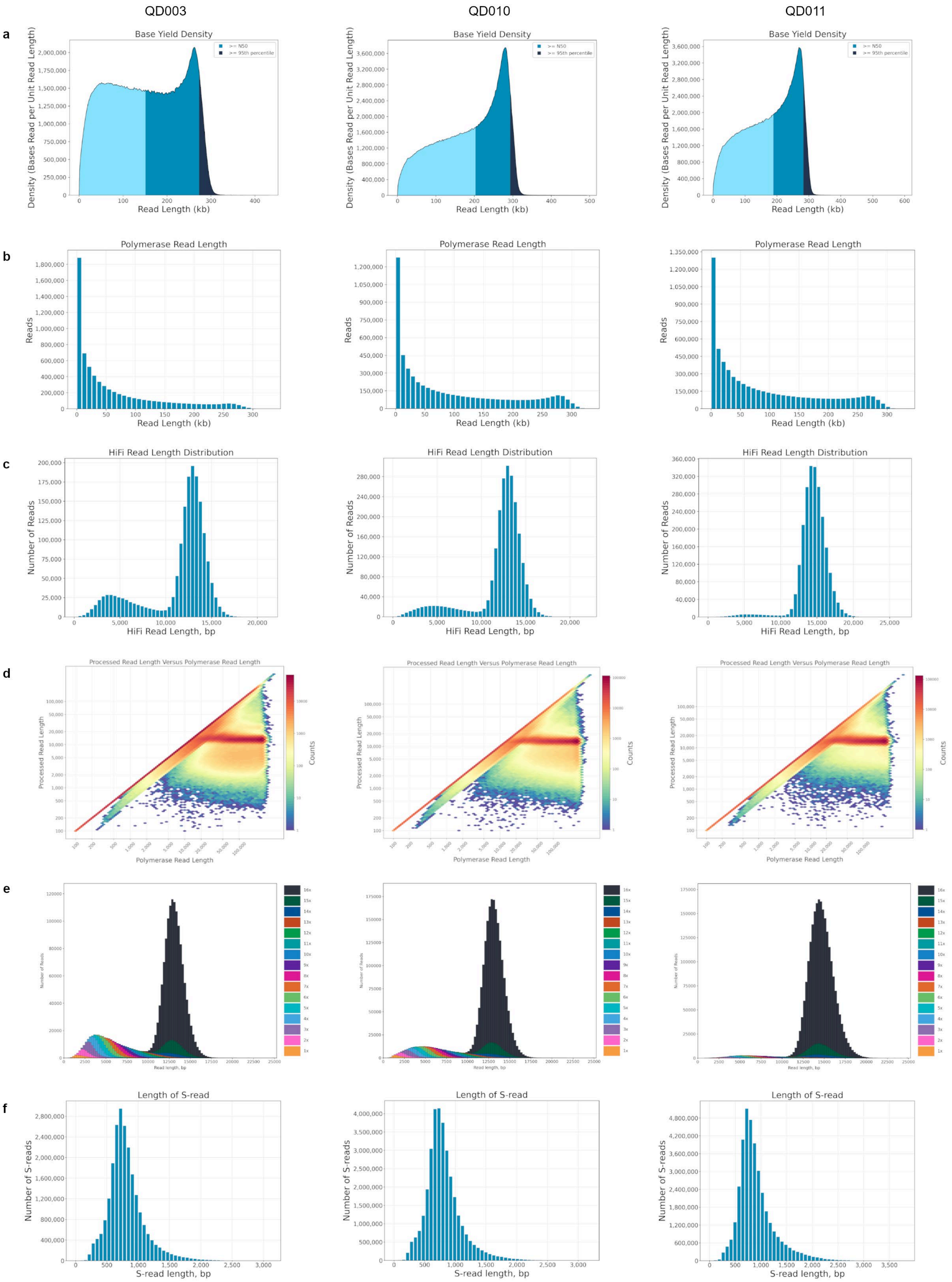
QD003
QD010
QD011
a
b
c
d
e
f
Base Yield Density
>= N50
>= 95th percentile
Density (Bases Read per Unit Read Length)
Read Length (kb)
Polymerase Read Length
Reads
HiFi Read Length Distribution
Number of Reads
HiFi Read Length, bp
Processed Read Length Versus Polymerase Read Length
Processed Read Length
Polymerase Read Length
Counts
Read length, bp
Length of S-read
Number of S-reads
S-read length, bp

Supplementary Fig. 10 | Quality check metrics of the VR03 datasets from three independent MAS-IsoSeq runs. The SMRTlink metrics are **(a)** base yield density, **(b)** read count by polymerase read length, **(c)** HiFi read length distribution, **(d)** processed read length versus polymerase read length, **(e)** HiFi read count distribution by read length, grouped by array size, and **(f)** segmented-read length distribution.

Supplementary Tables

Supplementary Table 1. Sequencing metrics and quality check of MAS-seq using PacBio HiFi Sequel IIe.

The sequencing metrics of three independent MAS-IsoSeq runs.

ID	Library length (bp)	Productivity P1 (%)	Polymerase				Unique molecular yield
			Read bases	Reads	Read length (mean)	Read length N50	
QD003	12,900	84.85	428,343,803,543	6,799,734	151,750	151,750	117,875,900,416
QD010	12,210	70.62	516,491,691,157	5,658,924	203,250	203,250	80,270,983,168
QD011	14,821	78.84	556,125,951,600	6,317,280	189,250	189,250	96,864,698,368

Quantity and quality of HiFi reads generated from PacBio subreads.

ID	Subreads		HiFi reads				
	Mean read length (bp)	N50	Reads	Read bases	Read length (mean)	Read quality (median)	Passes (mean)
QD003	19,040	31,250	1,689,541	19,120,152,122	11,384	Q31	13
QD010	16,068	16,750	2,289,154	27,599,575,639	12,056	Q32	13
QD011	16,916	17,250	2,362,046	34,112,056,419	14,441	Q31	11

Numbers of segmented reads and unique isoforms after array segmentation, deduplication, collapsing, and filtering using IsoSeq3 and Pigeon analysis tools.

	QD003	QD010	QD011
**Reads segmentation** (Skera split)	22,772,310	32,985,296	36,772,491
**Group & deduplication** (IsoSeq3 dedup)	38,888,149		
**Collapses into unique isoforms** (IsoSeq3 collapse)	651,288 isoforms		
**Isoforms after filtering** (Pigeon filter)	285,638 isoforms		

Supplementary Table 2. Nomenclature of the terms used in the study.

Terms	Acronym	First appearance	Definition
Isoform		Introduction	Different forms of transcript from the same gene that result from alternative splicing.
Transcript		Introduction	A physical molecule of RNA that can be sequenced.
Gene model		Introduction	The structure and location of a gene on the genome. It contains the information of chromosome, start, end, and strand of the gene and exon of each isoform.
Full-length isoform sequencing		Introduction	A method that captures and sequences the complete transcripts from the 3' end (characterized by the presence of poly-A tail) without fragmentation into several shorter transcripts. This ensures the entire exon structure of a single transcript is preserved.
B73 RefGen_v5		Introduction	The official and latest (5-th) version of gene-model database for the *Zea mays* B73 inbred line. The database is provided in GTF.
HiFi sequencing		Introduction	A technology by Pacific Biosciences (PacBio) that produces highly accurate (>99.9%), long-read sequencing data. It circles and sequences a molecule multiple times to obtain the consensus reads (CCS) to drastically reduces sequencing error.
Multiplexed Arrays sequencing	MAS-seq	Introduction	Concatenating multiple cDNA molecules into one PacBio library fragment for HiFi sequencing. In this study, MAS-Seq is combined with spatial transcriptomics by concatenating amplified cDNA with 10x Genomics Visium barcode and the concatenation factor is 16-mer.
Full-Splice Match	FSM	Results: Characterization of maize embryonic leaf	An isoform that matches all the splice sites of a known gene model in B73 RefGen_v5 database.
Incomplete Splice Match	ISM	Results: Characterization of maize embryonic leaf	A novel isoform that matches a known gene model in B73 RefGen_v5 database but missing one or more exons at the 5' and/or 3' regions.
Novel In Catalog	NIC	Results: Characterization of maize embryonic leaf	A novel isoform that uses known splice sites and exons but combines them in a way that are not recorded in B73 RefGen_v5 database.
Novel Not in Catalog	NNC	Results: Characterization of maize embryonic leaf	A novel isoform that contains at least one novel splice site that are not recorded in B73 RefGen_v5 database.
Novel isoform		Results: Characterization of maize embryonic leaf	An isoform with any splice sites, 5' or 3' exons, or exon combinations that are not recorded in B73 RefGen_v5 reference gene-model database. It is categorized under one of the structure categories: “ISM”, “NIC”, and “NNC”.
LRiso		Results: Characterization of maize embryonic leaf	A precursor gene-model database that constructed from PacBio IsoSeq analysis pipeline. The included gene models are

			generated solely based on transcripts sequenced via full-length isoform sequencing. The database is provided in GTF.
Accessible Chromatin Region	ACR	Results: Functional assessment of novel isoforms	A segment of the genome where the DNA is "open", or nucleosome-depleted and physically reachable by cellular machinery, such as transcription factors and RNA polymerase.
Assay for Transposase-Accessible Chromatin sequencing	ATAC-seq	Results: Functional assessment of novel isoforms	A genome-wide assay that utilizes a hyperactive Tn5 transposase to simultaneously cut and ligate sequencing adapters into nucleosome-depleted regions of the genome. A standard method for mapping ACRs to identify active regulatory elements across the genome.
Uniform Manifold Approximation and Projection	UMAP	Results: Unsupervised clustering of isoforms	A non-linear dimensionality reduction algorithm that preserves the local and global topological structure of high-dimensional data for low-dimensional visualization and clustering.
Gibbs free folding energy	ΔG	Results: Unsupervised clustering of isoforms	A thermodynamic quantity representing the difference in free energy between the folded and unfolded states of a macromolecule, where a negative value indicates that the folded conformation is energetically favorable and spontaneous under specific cellular conditions.
Module eigen-isoform	MEiso	Results: Isoform-level weighted gene co-expression network analysis	An eigen-isoform is the principal component (first eigenvector) derived from a singular value decomposition (SVD) of an isoform expression matrix within a co-expression module. It serves as a single, representative expression profile that captures the maximum variance and primary regulatory trend of all isoforms in that specific group.
Novel gene		Results: Construction of MaizeV5_IsoAnn	A gene model located within the intergenic region of the B73 RefGen_v5 reference gene-model database.
MaizeV5_IsoAnn		Results: Construction of MaizeV5_IsoAnn	Isoform-annotated (IsoAnn) gene-model databases that constructed using B73 RefGen_v5 as the foundation. The database consists of novel genes and genes with 5' and/or 3' flanking region extensions on top of the gene models in B73 RefGen_v5.
Laser-Captured Microdissection	LCM	Results: Re-analysis of LCM-captured embryonic vein RNA-Seq	A high-precision technique where a laser is used to cut out and isolate a specific, tiny group of cells (or even a single cell) from a tissue sample under a microscope.
Time-Ordered Gene Co-expression Network	TO-GCN	Results: Re-analysis of LCM-captured embryonic vein RNA-Seq	A bioinformatics framework used to model the dynamic regulatory relationship between transcription factors and genes across different time points. It provides a directional and chronological layer to the co-expression network.

Transcription Factor Binding Site	TFBS	Results: *LBD26* emerges as a putative regulator	A short sequence of DNA where a transcription factor physically attaches to that site of genome. It acts as a “molecular switch” where it helps recruits or block the RNA polymerase for transcription, thereby turning a gene “on” or “off”.
Position Weight Matrix	PWM	Results: *LBD26* emerges as a putative regulator	A probabilistic representation of the nucleic acid sequence specificity of a DNA-binding protein. It calculates the log-likelihood ratio (log-odds score) of observing a specific nucleotide at each position within a motif versus a background genomic distribution.
DNA Affinity Purification sequencing	DAP-seq	Results: *LBD26* emerges as a putative regulator	An *in vitro* high-throughput assay that couples affinity-purified transcription factors (purified from *in vitro* expression of a cDNA clone) with shotgun genomic DNA libraries to identify physical binding sites.

Supplementary Table 3. Comparison of 5'- and 3'-end locations between B73 RefGen_v5 and LRiso gene models. Gene models featuring an extended 3' terminus are denoted by an asterisk (*).

	Number of gene models	Percentage
Extended 5'- & 3'- end*	5,001	18.91%
Extended 5'- & shrunk 3'- end	7,660	28.97%
Shrunk 5'- & extended 3'- end*	7,847	29.68%
Shrunk 5'- & 3' end	4,860	18.38%
Extended 5'- end	224	0.85%
Shrunk 5'- end	232	0.88%
Extended 3'- end*	296	1.12%
Shrunk 3' end	299	1.13%
No change	21	0.08%
Total	26,440	100.00%

Supplementary Table 8. Intersection of up-regulated isoforms across vein tissue clusters. Summary of overlapping up-regulated isoforms identified by comparing specific vein tissue clusters (col_v, embry_v1, and embry_v2) against all other clusters (including the SAM, primordial leaves, and non-target vein tissues).

Transcript ID	Gene ID	Gene description
**col_v** and **embry_v1** and **embry_v2**		
PB.21971.1:	Zm00001eb163000	Callose synthase2
PB.34583.1:	Zm00001eb254100	GDSL esterase/lipase
**col_v** and **embry_v1**		
PB.46752.2:	Zm00001eb342500	Uncharacterized protein
PB.15015.2:	Zm00001eb111390	Phylloplanin
PB.31872.2:	Zm00001eb234730	Brown midrib (*BM1*)
PB.34516.2:	Zm00001eb253610	Non-coding gene
PB.35455.10:	Zm00001eb261140	Multidrug and toxic compound extrusion 1 (*MATE1*)
PB.36064.1:	Zm00001eb265260	Tetraspanin-6
PB.39480.1:	Zm00001eb287600	Oxidoreductase 2OG-Fe(II) oxygenase family protein
PB.49010.1:	Zm00001eb359290	Uncharacterized protein
PB.54500.18:	Zm00001eb101230	Phospholipid transfer protein 53 (*PLT53*)
**col_v** and **embry_v2**		
PB.12374.4:	Zm00001eb091360	Dirigent protein
PB.13698.2:	Zm00001eb100630	HVA22-like protein
PB.17156.1:	Zm00001eb129030	Substilin 9 (*SBT9*)
PB.17436.1:	Zm00001eb130940	Guaiacol peroxidase1 (*POX1*)
PB.18488.14:	Zm00001eb136530	Osnotin-like protein
PB.18964.3:	Zm00001eb139660	Protein kinase domain-containing protein
PB.21068.4:	Zm00001eb155680	AWPM-19-like family protein
PB.21068.8:	Zm00001eb155680	AWPM-19-like family protein
PB.21445.1:	Zm00001eb158730	Protein MIZU-KUSSEI 1
PB.22814.10:	Zm00001eb169960	Putative membrane lipoprotein
PB.28718.1:	Zm00001eb211120	Uncharacterized protein
PB.28853.3:	Zm00001eb212510	NDR1/HIN1-like protein
PB.29186.1:	Zm00001eb215410	Protein Accelerated cell death 6
PB.31002.1:	Zm00001eb228780	Sucrose synthase 7 (*SUS7*)
PB.31267.1:	Zm00001eb230930	Pentatricopeptide repeat-containing protein-like
PB.31541.1:	Zm00001eb232740	Early nodulin-like protein 3 (*NODL3*)
PB.36704.1:	Zm00001eb269200	Late embryogenesis abundant protein LEA-2 subgroup domain-containing protein
PB.3706.2:	Zm00001eb026680	SHSP domain-containing protein
PB.388.1:	Zm00001eb285830	protein phosphatase homolog 22 (*PRH22*)
PB.40003.3:	Zm00001eb294860	Reticulon-like protein
PB.43906.1:	Zm00001eb320820	NAC-transcription factor 18 (*NACTF18*)
PB.43963.1:	Zm00001eb321210	Glutathione S-transferase 56 (*GST56*)
PB.44541.1:	Zm00001eb325780	Non-coding gene
PB.46700.1:	Zm00001eb342070	Peroxidase 24

PB.50211.83:	Zm00001eb369700	Early nodulin-like protein 9 (*NODL9*)
PB.5427.3:	Zm00001eb037890	HVA22-like protein
PB.55171.1:	Zm00001eb407460	Uncharacterized protein
PB.55933.1:	Zm00001eb412420	Photosynthetic NDH subunit of subcomplex B1
PB.58097.3:	Zm00001eb428010	P-loop containing nucleoside triphosphate hydrolase superfamily protein
PB.6174.1:	Zm00001eb043120	L-glutonolactone oxidase
PB.6697.4:	Zm00001eb047490	Small heat shock-like protein

**embry_v1** and **embry_v2**		
PB.13144.7:	Zm00001eb096690	Mut7-C RNAse domain-containing protein
PB.13859.11:	Zm00001eb101710	Uncharacterized protein
PB.14235.23:	Zm00001eb104680	Transmembrane protein
PB.14642.15:	Zm00001eb108220	Nucleotide sugar transporter-KT 1
PB.14760.7:	Zm00001eb109290	beta-ketoacyl-[acyl-carrier-protein] synthase I
PB.20110.1:	Zm00001eb148450	Uncharacterized protein
PB.20649.16:	Zm00001eb152360	DUF2039 family protein
PB.21954.7:	Zm00001eb162890	1,3-beta-glucan synthase
PB.21959.70:	Zm00001eb162910	Pre-mRNA-processing protein 40A
PB.22243.3:	Zm00001eb165350	Uncharacterized protein
PB.24072.10:	Zm00001eb178690	Hypersensitive induced reaction 1 (*HIR1*)
PB.24228.2:	Zm00001eb179690	G2-like-transcription factor 49 (*GLK49*)
PB.24411.19:	Zm00001eb180540	Actin associated protein
PB.28139.17:	Zm00001eb205920	Regulator of chromosome condensation 19 (*RCC19*)
PB.30343.56:	Zm00001eb224300	Uncharacterized protein
PB.30725.14:	Zm00001eb226730	Serrate ortholog 4 (*SERT4*)
PB.30979.29:	Zm00001eb228620	High mobility group protein 1 (*HMG1*)
PB.33021.55:	Zm00001eb241510	UPF0548 protein
PB.33530.35:	Zm00001eb245590	Protein fluorescent in blue light chloroplastic
PB.33563.26:	Zm00001eb245950	Next to brca1a-like (*NBR1a*)
PB.3585.9:	Zm00001eb026090	Multidrug and toxic compound extrusion 8 (*MATE8*)
PB.37121.26:	Zm00001eb271970	MYB-related-transcription factor 98 (*MYBR98*)
PB.37207.9:	Zm00001eb272640	WAT1-related protein
PB.37839.16:	Zm00001eb278100	RNA-binding KH domain-containing protein
PB.38857.30:	Zm00001eb285610	40S ribosomal protein S5-2;
PB.39565.8:	Zm00001eb290800	Uncharacterized protein
PB.40241.22:	Zm00001eb296950	O-fucosyltransferase family protein
PB.42117.4:	Zm00001eb309310	Zinc finger family protein
PB.44037.13:	Zm00001eb321740	Vacuolar protein sorting-associated protein 32 homolog 1
PB.47316.22:	Zm00001eb346550	PfkB-like carbohydrate kinase family protein
PB.50245.24:	Zm00001eb369940	Aspartyl protease 2 (*AED2*)
PB.50589.13:	Zm00001eb372540	Pentatricopeptide repeat protein 459 (*PPR459*)
PB.55144.27:	Zm00001eb407100	DNA primase large subunit
PB.58228.1:	Zm00001eb429210	Amino acid/auxin permease 71 (*AAAP71*)
PB.58356.30:	Zm00001eb430580	26S proteasome non-ATPase regulatory subunit 1 homolog
PB.5961.13:	Zm00001eb041760	Regulator of chromosome condensation 22 (*RCC22*)
PB.7202.15:	Zm00001eb051500	NADP-dependent oxidoreductase domain-containing protein

Supplementary Table 9. Marker isoforms in the embryo_v1 cluster. Isoforms highlighted in bold are explicitly discussed in the main text. Selection of marker isoforms was determined using a significance threshold of adjusted *p-value* < 0.05 (Bonferroni correction).

Isoform ID	*p-value*	Avg. log2FC	*Adj. p-val*	Isoform fraction	Isoform structure	Gene ID	Gene description
**PB.11100.3**	**1.06^{-10}**	**1.859**	**2.75^{-5}**	**0.981**	**Novel in catalog**	**Zm00001eb083610**	**phospholipid transfer protein20 (*PLT20*)**
**PB.23596.1**	**7.46^{-13}**	**1.716**	**1.94^{-7}**	**0.745** [a]	**Full-splice match**	**Zm00001eb175860**	**WAT1-related protein**
PB.11118.1	1.06^{-18}	1.672	2.75^{-13}	0.912	Novel not in catalog	Zm00001eb083790	miniature seed1 (*MN1*)
**PB.12312.1**	**5.90^{-10}**	**1.471**	**1.54^{-4}**	**0.933**	**Full-splice match**	**Zm00001eb090910**	**phospholipid transfer protein21 (*PLT21*)**
**PB.11100.11**	**1.40^{-16}**	**1.287**	**3.65^{-11}**	**0.975**	**Novel not in catalog**	**Zm00001eb083620**	**lipid binding protein1 (*LBP1*)**
**PB.39999.1**	**8.42^{-13}**	**1.266**	**2.19^{-7}**	**1.000**	**Full-splice match**	**Zm00001eb294880**	**lipid transfer protein1 (*LTP1*)**
**PB.23107.1**	**2.01^{-8}**	**1.184**	**5.23^{-3}**	**1.000**	**Full-splice match**	**Zm00001eb172420**	**brown midrib 3 (*BM3*)**
PB.9186.2	3.16^{-9}	1.119	8.22^{-4}	0.995	Full-splice match	Zm00001eb068280	Isoaspartyl peptidase/L-asparaginase 2
PB.56674.1	2.67^{-11}	1.029	6.93^{-6}	0.381 [a]	Novel in catalog	Zm00001eb417160	Uncharacterized protein
PB.56674.2	4.58^{-15}	1.011	1.19^{-9}	0.521 [a]	Novel not in catalog	Zm00001eb417160	Uncharacterized protein
PB.46752.2	1.11^{-10}	0.755	2.89^{-5}	0.920	Full-splice match	Zm00001eb342500	Uncharacterized protein
**PB.23596.2**	**5.56^{-8}**	**0.712**	**1.45^{-2}**	**0.216** [a]	**Incomplete-splice match**	**Zm00001eb175860**	**WAT1-related protein**
**PB.54500.18**	**5.43^{-8}**	**0.628**	**1.41^{-2}**	**0.985**	**Full-splice match**	**Zm00001eb401230**	**phospholipid transfer protein53 (*PLT53*)**
**PB.19976.2**	**3.01^{-8}**	**0.625**	**7.84^{-3}**	**0.403** [a]	**Incomplete-splice match**	**Zm00001eb147480**	**auxin transporter-like 1 (*ATL1*)**
**PB.31872.2**	**5.67^{-11}**	**0.605**	**1.47^{-5}**	**1.000**	**Novel in catalog**	**Zm00001eb234730**	**brown midrib1 (*BM1*)**
PB.15212.50	6.14^{-16}	0.604	1.60^{-10}	0.880	Full-splice match	Zm00001eb112750	substilisin7 (*SBT7*)
**PB.31716.3**	**4.10^{-10}**	**0.569**	**1.07^{-4}**	**0.954**	**Full-splice match**	**Zm00001eb233720**	**brown midrib 5 (*BM5*)**
PB.52327.1	8.06^{-8}	0.557	2.10^{-2}	0.608	Full-splice match	Zm00001eb384970	skewed root growth similar18 (*SKUS18*)
PB.34583.1	3.15^{-14}	0.515	8.19^{-9}	0.833	Full-splice match	Zm00001eb254100	GSDL-motif protein lipase
PB.49424.1	6.22^{-10}	0.489	1.62^{-4}	0.951	Novel not in catalog	Zm00001eb362730	Nucleoid DNA-binding-like protein
PB.5306.1	2.13^{-12}	0.452	5.55^{-7}	0.885	Novel not in catalog	Zm00001eb036880	sugar will eventually be exported transporter11 (*SWEET11a*)

**PB.36064.1**	**3.69^{-8}**	**0.442**	**9.60^{-3}**	**0.793** [a]	**Full-splice match**	**Zm00001eb265260**	**Tetraspanin-6**
**PB.58228.1**	**3.21^{-17}**	**0.440**	**8.34^{-12}**	**0.593** [a]	**Novel not in catalog**	**Zm00001eb429210**	**Amino acid/auxin permease71 (*AAAP71*)**
PB.32073.1	3.43^{-11}	0.418	8.91^{-6}	0.984	Full-splice match	Zm00001eb235490	MAPK kinase substrate protein
**PB.29494.1**	**8.82^{-8}**	**0.410**	**2.29^{-2}**	**1.000**	**Full-splice match**	**Zm00001eb218120**	**LBD-transcription factor 26 (*LBD26*)**
PB.13213.3	2.63^{-8}	0.378	6.85^{-3}	0.439 [a]	Novel in catalog	Zm00001eb097230	GlcNAc-PI synthesis protein
PB.2813.1	1.39^{-13}	0.346	3.61^{-8}	1.000	Full-splice match	Zm00001eb021090	Fe2OG dioxygenase domain-containing protein
PB.2240.3	1.52^{-7}	0.335	3.94^{-2}	0.100 [a]	Novel in catalog	Zm00001eb016560	AAA-type ATPase family protein
**PB.23596.3**	**7.36^{-11}**	**0.323**	**1.91^{-5}**	**0.034**	**Incomplete-splice match**	**Zm00001eb175860**	**WAT1-related protein**
**PB.12312.2**	**1.19^{-12}**	**0.320**	**3.10^{-7}**	**0.047** [a]	**Full-splice match**	**Zm00001eb090910**	**phospholipid transfer protein21 (*PLT21*)**
PB.56389.3	2.52^{-10}	0.317	6.56^{-5}	0.982	Full-splice match	Zm00001eb414990	Uncharacterized protein
PB.33989.3	1.58^{-9}	0.312	4.11^{-4}	0.016 [a]	Full-splice match	Zm00001eb249350	Nucleolar essential protein-related
**PB.40897.1**	**2.73^{-23}**	**0.311**	**7.10^{-18}**	**0.929**	**Full-splice match**	**Zm00001eb302760**	**phospholipid transfer protein50 (*PLT50*)**
PB.50867.29	4.80^{-8}	0.305	1.25^{-2}	1.000	Novel in catalog	Zm00001eb374670	Ubiquitin carboxyl-terminal hydrolase
PB.45156.2	3.20^{-8}	0.297	8.32^{-3}	0.108 [a]	Novel in catalog	Zm00001eb331130	importin6 (*IMP6*)
PB.30300.3	2.65^{-8}	0.290	6.88^{-3}	0.500 [a]	Novel not in catalog	Zm00001eb224010	Histidine kinase3 (*HK3*)
PB.54432.8	4.52^{-8}	0.285	1.18^{-2}	0.514 [a]	Novel not in catalog	Zm00001eb400700	Adenosylmethionine decarboxylase
PB.9026.1	2.59^{-9}	0.283	6.73^{-4}	0.714 [a]	Full-splice match	Zm00001eb066890	AP2-EREBP-transcription factor 220 (*EREB220*)
PB.39141.7	3.13^{-12}	0.281	8.13^{-7}	0.221 [a]	Incomplete-splice match	Zm00001eb287600	Oxidoreductase 2OG-Fe(II) oxygenase family protein
PB.12585.1	1.61^{-21}	0.280	4.19^{-16}	1.000	Full-splice match	Zm00001eb092670	Uncharacterized protein
**PB.37069.10**	**7.25^{-18}**	**0.278**	**1.89^{-12}**	**0.462** [a]	**Full-splice match**	**Zm00001eb271530**	**Aux/IAA-transcription factor 25 (*IAA25*)**
PB.33578.1	2.94^{-8}	0.261	7.64^{-3}	0.968	Full-splice match	Zm00001eb246000	NPH3 domain-containing protein
**PB.19976.16**	**1.03^{-12}**	**0.257**	**2.68^{-7}**	**0.033** [a]	**Incomplete-splice match**	**Zm00001eb147480**	**auxin transporter-like 1 (*ATL1*)**

Avg. Log2FC: average log_2 fold change

[a] non-single dominant isoform (IF < 0.8)

Supplementary Table 10. Marker isoforms for the embry_v2 cluster. Isoforms highlighted in bold are explicitly discussed in the main text. Selection of marker isoforms was determined using a significance threshold of *p-value* < 0.05 (Bonferroni correction).

Isoform ID	*p-value*	Avg. log2FC	*Adj. p-val*	Isoform fraction	Isoform structure	Gene ID	Gene description
PB.13194.1	6.06^{-8}	1.538	1.58^{-2}	0.981	Novel in catalog	Zm00001eb097070	Dirigent protein
PB.32064.2	2.35^{-10}	1.172	6.11^{-5}	0.914	Full-splice match	Zm00001eb235450	Ran BP2/NZF zinc finger-like superfamily protein
**PB.28104.2**	$\mathbf{3.38^{-8}}$	**1.124**	$\mathbf{8.79^{-3}}$	**0.373** [a]	**Full-splice match**	**Zm00001eb205550**	**putative zinc finger protein30 (*ZFP30*)**
**PB.28104.37**	$\mathbf{3.53^{-8}}$	**1.101**	$\mathbf{9.19^{-3}}$	**0.372** [a]	**Novel in catalog**	**Zm00001eb205550**	**putative zinc finger protein30 (*ZFP30*)**
PB.17436.1	1.97^{-11}	1.087	5.13^{-6}	0.969	Full-splice match	Zm00001eb130940	guaiacol peroxidase1 (*POX1*)
PB.46700.1	9.88^{-11}	0.939	2.57^{-5}	0.945	Full-splice match	Zm00001eb342070	peroxidase24
PB.31267.1	9.35^{-16}	0.928	2.43^{-10}	0.914	Full-splice match	Zm00001eb230930	Pentatricopeptide (RRP) repeat-containing protein-like
**PB.388.1**	$\mathbf{2.73^{-12}}$	**0.907**	$\mathbf{7.09^{-7}}$	**0.987**	**Full-splice match**	**Zm00001eb002780**	**glutathione S-transferase11 (*GST11*)**
PB.17156.1	1.04^{-7}	0.837	2.70^{-2}	0.996	Full-splice match	Zm00001eb129030	substilisin 29 (*SBT29*)
**PB.50211.83**	$\mathbf{2.24^{-10}}$	**0.718**	$\mathbf{5.83^{-5}}$	**1.000**	**Full-splice match**	**Zm00001eb369700**	**early nodulin-like protein 9**
PB.21971.1	3.01^{-9}	0.715	7.82^{-4}	1.000	Incomplete-splice match	Zm00001eb163000	callose synthase (*CALS2*)
**PB.28104.40**	$\mathbf{6.39^{-10}}$	**0.627**	$\mathbf{1.66^{-4}}$	**0.053** [a]	**Novel in catalog**	**Zm00001eb205550**	**putative zinc finger protein30 (*ZFP30*)**
PB.31002.1	1.42^{-9}	0.560	3.69^{-4}	0.616 [a]	Incomplete-splice match	Zm00001eb228780	sucrose synthease 7 (*SUS7*)
PB.40003.3	3.35^{-11}	0.520	8.71^{-6}	0.614 [a]	Incomplete-splice match	Zm00001eb294860	Uncharacterized protein
**PB.28104.43**	$\mathbf{3.03^{-11}}$	**0.419**	$\mathbf{7.88^{-6}}$	**0.023** [a]	**Incomplete-splice match**	**Zm00001eb205550**	**putative zinc finger protein 30 (*ZFP30*)**
PB.21068.4	3.38^{-8}	0.372	8.78^{-3}	0.420 [a]	Full-splice match	Zm00001eb155680	AWPM-19-like family protein
**PB.43963.1**	$\mathbf{7.17^{-8}}$	**0.348**	$\mathbf{1.87^{-2}}$	**0.962**	**Novel in catalog**	**Zm00001eb321210**	**glutathione S-transferase 56 (*GST56*)**
PB.21068.8	7.81^{-8}	0.328	2.03^{-2}	0.312 [a]	Full-splice match	Zm00001eb155680	Membrane protein PM19L
PB.58097.3	5.60^{-11}	0.313	1.46^{-5}	0.887	Novel in catalog	Zm00001eb428010	nucleoside triphosphate hydrolase superfamily protein
PB.55171.1	1.61^{-7}	0.311	4.19^{-2}	0.885	Full-splice match	Zm00001eb407460	Uncharacterized protein
PB.5427.3	1.98^{-08}	0.304	5.15^{-3}	0.645 [a]	Full-splice match	Zm00001eb037890	HVA22-like protein
PB.18341.1	7.45^{-9}	0.284	1.94^{-3}	1.000	Full-splice match	Zm00001eb135640	chitinase 30 (*CHN30*)
**PB.31541.1**	$\mathbf{7.21^{-12}}$	**0.270**	$\mathbf{1.88^{-6}}$	**1.000**	**Full-splice match**	**Zm00001eb232740**	**early nodulin-like protein 3**
PB.3706.2	5.50^{-11}	0.250	1.43^{-5}	0.172 [a]	Full-splice match	Zm00001eb026680	SHSP domain-containing protein

Avg. Log2FC: average log_2 fold change

[a] non-single dominant isoform (IF < 0.8)

Supplementary Table 12. Enriched biological process GO terms associated with the hub isoforms of each module.

Term ID	Term name	Term size	Intersection size	Enrichment ratio	*p-value*	Module	Module color
GO:0009734	Auxin-activated signaling pathway	176	6	0.034	2.20E-02	M2	black
GO:0010103	Stomatal complex morphogenesis	22	3	0.136	4.00E-02	M2	black
GO:0090558	Plant epidermis development	62	4	0.065	4.40E-02	M2	black
GO:0045292	mRNA cis splicing, via spliceosome	43	5	0.116	3.60E-04	M3	yellow
GO:0016043	Cellular component organization	3025	29	0.010	6.40E-04	M3	yellow
GO:0007018	Microtubule-based movement	109	6	0.055	2.20E-03	M3	yellow
GO:0006302	Double-stand break repair	170	7	0.041	2.30E-03	M3	yellow
GO:0022402	Cell cycle process	466	10	0.021	6.40E-03	M3	yellow
GO:0051276	Chromosome organization	510	10	0.020	1.40E-02	M3	yellow
GO:0006268	DNA unwinding involved in DNA replication	20	3	0.150	3.40E-02	M3	yellow
GO:0006260	DNA replication	267	7	0.026	4.20E-02	M3	yellow
GO:0022613	Ribonucleoprotein complex biogenesis	643	20	0.031	1.10E-09	M4	magenta
GO:1901566	Organonitrogen compound biosynthetic process	2294	30	0.013	4.10E-06	M4	magenta
GO:0006913	Nucleocytoplasmic transport	154	6	0.039	3.00E-02	M4	magenta
GO:0019676	Ammonia assimilation cycle	3	2	0.667	3.30E-02	M4	magenta
GO:0044238	Primary metabolic process	13846	75	0.005	4.40E-02	M4	magenta
GO:0051052	Regulation of DNA metabolic process	72	4	0.056	3.10E-02	M5	blue
GO:0006298	Mismatch repair	30	3	0.100	5.00E-02	M5	blue
GO:0006301	Post-replication repair	30	3	0.100	5.00E-02	M5	blue
GO:0006325	Chromatin organization	425	37	0.087	4.10E-42	M7	brown
GO:0045910	Negative regulation of DNA recombination	20	3	0.150	2.20E-02	M7	brown
GO:0070972	Protein localization to endoplasmic reticulum	71	6	0.085	3.40E-05	M10	purple
GO:0006888	Endoplasmic reticulum to Golgi vesicle-mediated transport	144	7	0.049	1.20E-04	M10	purple
GO:0090316	Positive regulation of intercellular protein transport	3	2	0.667	1.50E-02	M10	purple
GO:1901566	Organonitrogen compound biosynthetic process	2294	27	0.012	1.90E-06	M11	red

GO:0009773	Photosynthetic electron transport in photosystem I	17	3	0.176	1.60E-02	M11	red
GO:0019682	Glyceraldehyde-3-phosphate metabolic process	22	3	0.136	3.70E-02	M11	red
GO:0060567	Negative regulation of termination of DNA-templated transcription	4	2	0.500	3.80E-02	M12	midnightblue
GO:1901566	Organonitrogen compound biosynthetic process	2294	39	0.017	7.30E-17	M13	tan
GO:0022613	Ribonucleoprotein complex biogenesis	643	16	0.025	2.00E-07	M13	tan
GO:0019752	Carboxylic acid metabolic process	1046	15	0.014	1.00E-03	M13	tan
GO:0009060	Aerobic respiration	179	6	0.034	1.90E-02	M13	tan
GO:0015979	Photosynthesis	320	25	0.078	8.10E-26	M14	green
GO:0046500	S-adenosylmethionine metabolic process	9	4	0.444	6.80E-06	M14	green
GO:0009628	Response to abiotic stimulus	682	13	0.019	1.10E-04	M14	green
GO:0006412	Translation	1228	17	0.014	1.10E-04	M14	green
GO:0034059	Response to anoxia	10	3	0.300	2.10E-03	M14	green
GO:0006730	One-carbon metabolic process	37	4	0.108	3.30E-03	M14	green
GO:0009611	Response to wounding	80	5	0.063	1.00E-03	M15	turquoise
GO:0072330	Monocarboxylic acid biosynthetic process	219	6	0.027	1.10E-02	M15	turquoise
GO:0000272	Polysaccharide catabolic process	174	5	0.029	4.40E-02	M15	turquoise

Supplementary Table 15. Evaluation and categorization of novel gene models. Summary of novel genes identified by the LRiso pipeline, categorized across four distinct evaluation criteria.

Evaluation criterion	Counts	Percentage
Total novel gene models	6,878	100.00%
Novel gene models with BLASTP hit ($e < 1 \times 10^{-5}$)	4,631	67.33%
Novel gene models with PFAM support (≥ 1 isoform)	679	9.87%
Novel gene models with EviAnn annotation support	563	8.19%
Novel gene models expressed on ≥ 6 libraries (≥ 1 TPM)	1,846	26.84%
Total number of novel genes with support [a]	5,228	76.03%

[a] The final number of novel gene models indicate those genes with at least one supporting criterion.

Supplementary Table 19. Distribution of transcription factor (TF) genes across TO-GCN levels in the B73 RefGen_v5 and MaizeV5_IsoAnn databases.

B73 RefGen_v5	MaizeV5_IsoAnn	TF count	Transcription factors
L1	L2	8	Zm00001eb158520; Zm00001eb159750; Zm00001eb160120; Zm00001eb187880; Zm00001eb252180; Zm00001eb314630; Zm00001eb316800; Zm00001eb378270
L2	L1	3	Zm00001eb100410; Zm00001eb195490; Zm00001eb390920
L2	L3	11	Zm00001eb087220; Zm00001eb147030; Zm00001eb190870; Zm00001eb217480; Zm00001eb253650; Zm00001eb259370; Zm00001eb295060; Zm00001eb398190; Zm00001eb401210; Zm00001eb418630; Zm00001eb427850
L3	L2	5	Zm00001eb186280; Zm00001eb198510; Zm00001eb364360; Zm00001eb405590; Zm00001eb428830
L3	L4	10	Zm00001eb022070; Zm00001eb051340; Zm00001eb076420; Zm00001eb103000; Zm00001eb199110; Zm00001eb244720; Zm00001eb255860; Zm00001eb266370; Zm00001eb399810; Zm00001eb433000
L4	L3	12	Zm00001eb001990; Zm00001eb139970; Zm00001eb164360; Zm00001eb227830; Zm00001eb233320; Zm00001eb266360; Zm00001eb279090; Zm00001eb288360; Zm00001eb300360; Zm00001eb366790; Zm00001eb369060; Zm00001eb408000
L4	L5	8	Zm00001eb006890; Zm00001eb112900; Zm00001eb195420; Zm00001eb223920; Zm00001eb238140; Zm00001eb288080; Zm00001eb429090; Zm00001eb431350
L5	L1	1	Zm00001eb315950
L5	L3	1	Zm00001eb401610
L5	L4	14	Zm00001eb018880; Zm00001eb084720; Zm00001eb135910; Zm00001eb148570; Zm00001eb181760; Zm00001eb183190; Zm00001eb242090; Zm00001eb245030; Zm00001eb349120; Zm00001eb369190; Zm00001eb369550; Zm00001eb379950; Zm00001eb413920; Zm00001eb417170
L5	L6	11	Zm00001eb046790; Zm00001eb061030; Zm00001eb066260; Zm00001eb073910; Zm00001eb144290; Zm00001eb234180; Zm00001eb266750; Zm00001eb270930; Zm00001eb349450; Zm00001eb406660; Zm00001eb409800
L6	L1	1	Zm00001eb360510
L6	L2	1	Zm00001eb291620
L6	L5	14	Zm00001eb027800; Zm00001eb047750; Zm00001eb062670; Zm00001eb075510; Zm00001eb106630;

			Zm00001eb120740; Zm00001eb199780; Zm00001eb207450; Zm00001eb217350; Zm00001eb324300; Zm00001eb330710; Zm00001eb349010; Zm00001eb403140; Zm00001eb421530
L6	L7	14	Zm00001eb006160; Zm00001eb037960; Zm00001eb055350; Zm00001eb128760; Zm00001eb166400; Zm00001eb176840; Zm00001eb198620; Zm00001eb237820; Zm00001eb247570; Zm00001eb298680; Zm00001eb331080; Zm00001eb336500; Zm00001eb370770; Zm00001eb433040
L7	L5	1	Zm00001eb314890
L7	L6	18	Zm00001eb052070; Zm00001eb088080; Zm00001eb122550; Zm00001eb125090; Zm00001eb144270; Zm00001eb159390; Zm00001eb169130; Zm00001eb171720; Zm00001eb210530; Zm00001eb211700; Zm00001eb256690; Zm00001eb257170; Zm00001eb273020; Zm00001eb325760; Zm00001eb359470; Zm00001eb373660; Zm00001eb384320; Zm00001eb395640
L7	L8	3	Zm00001eb118010; Zm00001eb212880; Zm00001eb325740
L7	L10	1	Zm00001eb121010
L8	L7	18	Zm00001eb031310; Zm00001eb051120; Zm00001eb075300; Zm00001eb104750; Zm00001eb107920; Zm00001eb120150; Zm00001eb121290; Zm00001eb135750; Zm00001eb172880; Zm00001eb243450; Zm00001eb243910; Zm00001eb298990; Zm00001eb319580; Zm00001eb323440; Zm00001eb351690; Zm00001eb372960; Zm00001eb418410; Zm00001eb432460
L8	L9	3	Zm00001eb002490; Zm00001eb125330; Zm00001eb344600
L9	L7	1	Zm00001eb384820
L9	L8	9	Zm00001eb100360; Zm00001eb104260; Zm00001eb186650; Zm00001eb227390; Zm00001eb248900; Zm00001eb254070; Zm00001eb279120; Zm00001eb296470; Zm00001eb385620
L9	L10	1	Zm00001eb024300
L10	L9	7	Zm00001eb032370; Zm00001eb064730; Zm00001eb142060; Zm00001eb157100; Zm00001eb286410; Zm00001eb315850; Zm00001eb422470
NA	L2	1	Zm00001eb020610
NA	L3	3	Zm00001eb012700; Zm00001eb020630; Zm00001eb354750
NA	L6	1	Zm00001eb100380
NA	L10	1	Zm00001eb379110

Supplementary Table 20. GO enrichment of biological processes for non-TF genes co-expressed with transcription factors. Significance thresholds are provided as adjusted p-values calculated using the g:SCS algorithm—a specialized correction method within the g:Profiler2 suite designed to account for the hierarchical structure of GO terms.

Term ID	GO term	*Adj. p-value*	Enrichment ratio	TO-GCN level
GO:0006464	Protein modification process	2.72E-17	0.238	L1
GO:0034728	Nucleosome organization	8.71E-11	0.582	L1
GO:0015979	Photosynthesis	9.86E-11	0.347	L1
GO:0045934	Negative regulation of nucleobase-containing compound metabolic process	1.68E-05	0.344	L1
GO:0006511	Ubiquitin-dependent protein catabolic process	1.72E-03	0.249	L1
GO:0009299	mRNA transcription	4.83E-03	0.667	L1
GO:0036211	Protein modification process	2.45E-20	0.355	L2
GO:0015979	Photosynthesis	1.07E-05	0.422	L2
GO:0009765	Photosynthesis, light harvesting	2.34E-05	0.700	L2
GO:0034728	Nucleosome organization	2.98E-05	0.597	L2
GO:0010410	Hemicellulose metabolic process	2.40E-03	0.421	L2
GO:0009416	Response to light stimulus	3.13E-03	0.402	L2
GO:0009299	mRNA transcription	0.01	0.778	L2
GO:0045934	Negative regulation of nucleobase-containing compound metabolic process	0.01	0.418	L2
GO:0030001	Metal ion transport	0.02	0.374	L2
GO:1900150	Regulation of defense response to fungus	0.02	0.680	L2
GO:0042546	Cell wall biogenesis	0.03	0.407	L2
GO:0009606	Tropism	0.04	0.537	L2
GO:0051179	Localization	0.04	0.304	L2
GO:0006810	Transport	0.05	0.305	L2
GO:0036211	Protein modification process	3.62E-10	0.367	L3
GO:0051179	Localization	3.46E-07	0.353	L3
GO:0009832	Plant-type cell wall biogenesis	1.81E-04	0.539	L3
GO:0006865	Amino acid transport	2.91E-03	0.519	L3
GO:0010876	Lipid localization	0.01	0.469	L3
GO:0042221	Response to chemical	0.02	0.354	L3
GO:0030001	Metal ion transport	0.04	0.403	L3
GO:0051179	Localization	1.08E-12	0.296	L4
GO:0009832	Plant-type cell wall biogenesis	3.20E-06	0.496	L4
GO:0006468	Protein phosphorylation	7.85E-05	0.299	L4
GO:0030243	Cellulose metabolic process	1.11E-03	0.457	L4
GO:0005976	Polysaccharide metabolic process	5.37E-03	0.324	L4
GO:0009699	Phenylpropanoid biosynthetic process	0.01	0.473	L4
GO:0044282	Small molecule catabolic process	0.01	0.358	L4
GO:0044248	Cellular catabolic process	0.02	0.300	L4
GO:0098754	Detoxification	0.04	0.338	L4
GO:0051211	Anisotropic cell growth	0.04	1.000	L4
GO:0048878	Chemical homeostasis	0.04	0.346	L4
GO:0051179	Localization	4.08E-13	0.309	L5
GO:0009056	Catabolic process	1.44E-09	0.320	L5
GO:0005975	Carbohydrate metabolic process	3.14.E-04	0.311	L5
GO:0030243	Cellulose metabolic process	6.07E-04	0.477	L5
GO:0009699	Phenylpropanoid metabolic process	8.37E-04	0.430	L5
GO:0036211	Protein modification process	2.17E-03	0.290	L5
GO:0006793	Phosphorus metabolic process	3.30E-03	0.286	L5
GO:0009605	Response to external stimulus	4.42E-03	0.329	L5
GO:0006901	Vesicle coating	0.01	0.846	L5

GO:0141124	Intracellular signaling cassette	0.01	0.409	L5
GO:0051716	Cellular response to stimulus	0.02	0.287	L5
GO:0010243	Response to organonitrogen compound	0.05	0.600	L5
GO:0051641	Cellular localization	6.79E-16	0.402	L6
GO:1901575	Organic substance catabolic process	1.85E-15	0.389	L6
GO:0006996	Organelle organization	3.09E-08	0.367	L6
GO:0033554	Cellular response to stress	6.68E-07	0.381	L6
GO:1901135	Carbohydrate derivative metabolic process	2.48E-04	0.372	L6
GO:0009607	Response to biotic stimulus	3.99E-04	0.394	L6
GO:0043412	Macromolecule modification	0.01	0.326	L6
GO:0007264	Small GTPase-mediated signal transduction	0.01	0.557	L6
GO:0140029	Exocytic process	0.03	0.750	L6
GO:0044255	Cellular lipid metabolic process	0.03	0.357	L6
GO:0046496	Nicotinamide nucleotide metabolic process	0.03	0.430	L6
GO:0006396	RNA processing	2.20E-22	0.462	L7
GO:0009124	Nucleoside monophosphate biosynthetic process	9.53E-08	0.671	L7
GO:0033554	Cellular response to stress	1.20E-06	0.402	L7
GO:0015980	Energy derivation by oxidation of organic compounds	1.13E-03	0.472	L7
GO:0006573	Valine metabolic process	9.47E-03	0.800	L7
GO:0048229	Gametophyte development	0.02	0.534	L7
GO:0035825	Homologous recombination	0.03	0.563	L7
GO:1902652	Secondary alcohol metabolic process	0.04	0.857	L7
GO:0000209	Protein polyubiquitination	0.04	0.510	L7
GO:1901566	Organonitrogen compound biosynthetic process	2.68E-49	0.393	L8
GO:0071806	Protein transmembrane transport	8.81E-10	0.578	L8
GO:0042026	Protein refolding	8.95E-07	0.584	L8
GO:0009790	Embryo development	3.57E-06	0.552	L8
GO:0042793	Plastid transcription	6.59E-05	1.000	L8
GO:1901259	Chloroplast rRNA processing	1.44E-04	0.810	L8
GO:0090150	Establishment of protein localization to membrane	1.03E-03	0.365	L8
GO:0022414	Reproductive process	0.02	0.333	L8
GO:1901566	Organonitrogen compound biosynthetic process	1.28E-70	0.264	L9
GO:0009451	RNA modification	1.75E-32	0.348	L9
GO:0009657	Plastid organization	2.37E-15	0.401	L9
GO:0072655	Establishment of protein localization to mitochondrion	1.76E-10	0.474	L9
GO:0042026	Protein refolding	5.25E-07	0.416	L9
GO:1901259	Chloroplast rRNA processing	1.29E-06	0.714	L9
GO:0009790	Embryo development	9.37E-04	0.333	L9
GO:0006515	Protein quality control for misfolded or incompletely synthesized proteins	2.13E-03	0.571	L9
GO:0006839	Mitochondrial transport	8.90E-03	0.281	L9
GO:0009553	Embryo sac development	0.01	0.452	L9
GO:0042793	Plastid transcription	0.02	0.667	L9
GO:0006412	Translation	1.86E-48	0.139	L10
GO:0006364	rRNA processing	1.10E-13	0.148	L10
GO:0009451	RNA modification	3.60E-09	0.110	L10
GO:0009657	Plastid organization	4.21E-04	0.132	L10
GO:0006626	Protein targeting to mitochondrion	0.02	0.160	L10

Supplementary Table 23. Marker genes from a single-cell resolution spatial platform were compared with marker isoforms revealed in this study (Zhao et al., 2024).

Sub-cell type	Marker gene	Marker gene ID	Clusters	*p-val*	avg_log$_2$FC	*adj.p-val*	Marker Isoform
Procambium	*ZmPIN1a*	Zm00001eb372180	embryo_v1	0.004	0.455	1.000	*PIN1a.8*
	ZmLAX3	Zm00001eb010460	SAM_P1	4.66E-14	0.540	1.21E-08	*LAX3.1*
	ZmLAX2	Zm00001eb026490	embryo_v1	0.001	0.671	1.000	*LAX2.5*
	ZmMPa	Zm00001eb433020	embryo_v1	0.007	0.295	1.000	*ARF29.4*
	ZmWAT1-LIKE	Zm00001eb039040	NA	NA	NA	NA	NA
	ZmPHB	Zm00001eb050660	NA	NA	NA	NA	NA
	ZmXCP1	Zm00001eb267050	embryo_v1	0.002	1.274	1.000	*CCP5*.2
Phloem sieve 1	*ZmAPL*	Zm00001eb233310	NA	NA	NA	NA	NA
	ZmCVL2	Zm00001eb301160	NA	NA	NA	NA	NA
	ZmSMXL3	Zm00001eb422090	NA	NA	NA	NA	NA
	ZmSMXL5	Zm00001eb243020	NA	NA	NA	NA	NA
Metaphloem	*ZmAPL*	Zm00001eb233310	NA	NA	NA	NA	NA
	ZmC57	Zm00001eb403880	NA	NA	NA	NA	NA
	ZmNEN1	Zm00001eb223190	NA	NA	NA	NA	NA
Phloem parenchyma 1	*ZmAPL*	Zm00001eb233310	NA	NA	NA	NA	NA
	ZmATPP2-A1	Zm00001eb336120	NA	NA	NA	NA	NA
	ZmSPMS1	Zm00001eb383120	shoot_col	2.79E-08	0.379	0.007	*SPMS1*.1
	ZmSUT1	Zm00001eb005460	NA	NA	NA	NA	NA
	ZmTPS	Zm00001eb353280	NA	NA	NA	NA	NA
	ZmAAP6b	Zm00001eb349050	NA	NA	NA	NA	NA
	ZmAAP6c	Zm00001eb408350	NA	NA	NA	NA	NA
	ZmAAP6d	Zm00001eb145670	NA	NA	NA	NA	NA
Phloem parenchyma 2	*ZmAPL*	Zm00001eb233310	NA	NA	NA	NA	NA
	ZmATPP2-A1	Zm00001eb336120	NA	NA	NA	NA	NA
	ZmSPMS1	Zm00001eb383120	NA	NA	NA	NA	NA
	ZmTPS	Zm00001eb353280	NA	NA	NA	NA	NA
	ZmC77	Zm00001eb172410	NA	NA	NA	NA	NA
	ZmSWEET12	Zm00001eb408900	NA	NA	NA	NA	NA
Xylem	*ZmXCP1*	Zm00001eb267050	embryo_v1	0.002	1.274	1.000	*CCP5.2*
	ZmBM3	Zm00001eb172420	embryo_v1	3.16E-09	1.119	0.001	*BM3.2*
	ZmBM5	Zm00001eb233720	embryo_v1	4.10E-10	0.569	1.07E-04	*BM5.3*
Xylem parenchyma	*ZmAAP1*	Zm00001eb299500	NA	NA	NA	NA	NA
	ZmAAP6b	Zm00001eb349050	NA	NA	NA	NA	NA
	ZmAAP6c	Zm00001eb408350	NA	NA	NA	NA	NA
	ZmAAP6d	Zm00001eb145670	NA	NA	NA	NA	NA
vascular sclerenchyma 1	*ZmBM5*	Zm00001eb233720	embryo_v1	4.10E-10	0.569	1.07E-04	*BM5*.3
	ZmBK2	Zm00001eb393070	NA	NA	NA	NA	NA
	ZmBK4	Zm00001eb317090	NA	NA	NA	NA	NA
	ZmBM3	Zm00001eb172420	embryo_v1	3.16E-09	1.119	0.001	BM3.2
	ZmPAL	Zm00001eb247610	NA	NA	NA	NA	NA
vascular sclerenchyma 2	*ZmHB102*	Zm00001eb005440	NA	NA	NA	NA	NA
	ZmHB77	Zm00001eb377480	NA	NA	NA	NA	NA
	ZmGST13	Zm00001eb361250	NA	NA	NA	NA	NA
	ZmAP2A1	Zm00001eb000860	NA	NA	NA	NA	NA

Supplementary Table 24. Probes used for padlock-probe rolling circle amplification (RCA) in situ hybridization. Tm indicate the melting temperature (°C) of 5' region (5' Tm), 3' region (3' Tm), and target region (Target Tm) of the padlock probe.

Gene	Gene_id	Isoform		Target sequence	Padlock probe sequence (blue: 5'-3' binding; red: detection oligo binding site; green: backbone)	5' Tm	3' Tm	Target Tm
LBD26	Zm00001eb218120	Zm00001eb218120_T001	Canonical	AGATGGTCAGGCTCAGGATGACCAACGACT	TGAGCCTGACCATCTAGCGATCTGCGAGACCGTATGACAGTCCCAGTGTACCCACAAACCGTGATAGCGATCTGCGAGACCGTATAGTCGTTGGTCATCC	46.1	46.1	73.44
WAT1-RELATED	Zm00001eb175860	*WAT1-RELATED.1*	Canonical	CCTACTTTCTAGAGAAGAAAGACAGGCCGC	TCTCTAGAAAGTAGGCTAGTGCTGGATGATCGTCCGACAGTCCCAGTGTACCCACAAACCGTGATCTAGTGCTGGATGATCGTCCGCGGCCTGTCTTTCT	40.64	48.84	72.07
		WAT1-RELATED.2	Novel	GCTAGCTGATGATGTATATGAACTGCCTGC	ACATCATCAGCTAGCAGCGATCTGCGAGACCGTATGACAGTCCCAGTGTACCCACAAACCGTGATAGCGATCTGCGAGACCGTATGCAGGCAGTTCATAT	43.37	43.37	70.7
COX6a1	Zm00001eb058950	Zm00001eb058950_T001	Canonical	TTCTCTATTTGCGTATAGGATTATCGTCTT	TACGCAAATAGAGAACTAGTGCTGGATGATCGTCCGACAGTCCCAGTGTACCCACAAACCGTGATCTAGTGCTGGATGATCGTCCAAGACGATAATCCTA	37.9	37.9	65.24
		COX6a1.1	Novel	TTTCCTGTATCAACTATCATTCTATCAAGG	AGTTGATACAGGAAAAGCGATCTGCGAGACCGTATGACAGTCCCAGTGTACCCACAAACCGTGATAGCGATCTGCGAGACCGTATCCTTGATAGAATGAT	46.1	46.1	73.44
RPL35a	Zm00001eb374200	Zm00001eb374200_T001	Canonical	TCTGTTGCTTGTGATTGAATGACACCACAA	ATCACAAGCAACAGACTAGTGCTGGATGATCGTCCGACAGTCCCAGTGTACCCACAAACCGTGATCTAGTGCTGGATGATCGTCCTTGTGGTGTCATTCA	40.64	40.64	67.97
		RPL35a.1	Novel	TGTCCGATGCTATCTCTTCAACAGATCCCC	AGATAGCATCGGACAAGCGATCTGCGAGACCGTATGACAGTCCCAGTGTACCCACAAACCGTGATAGCGATCTGCGAGACCGTATGGGGATCTGTTGAAG	43.37	46.1	72.07

Reference

Liu, Y. *et al.* Constraint of accessible chromatins maps regulatory loci involved in maize speciation and domestication. *Nature Communications* **16**, 2477 (2025). https://doi.org/10.1038/s41467-025-57932-1

Zhao, C. *et al.* Comparative spatiotemporal single cell transcriptomes reveal rewiring of pre-existing regulations during emergence of Kranz anatomy in C4 grasses. *bioRxiv*, 2024.2010.2028.620769 (2024). https://doi.org/https://doi.org/10.1101/2024.10.28.620769
