# Supplementary material for: Profiling maize embryonic leaf development and discovering new genes using high-resolution spatial long-read isoform sequencing
Source: Nat Plants. 2026 Aug 17;12(9):1860–78. doi: 10.1038/s41477-026-02364-y (PMC13585551; doi:10.1038/s41477-026-02364-y)
Supplement: Supplementary file 2 [file 41477_2026_2364_MOESM2_ESM.pdf]

nature portfolio

Corresponding author(s): Dr. Wen-Hsiung Li and Dr. Mei-Yeh Jade Lu

Last updated by author(s): Jun 19, 2026

Reporting Summary

Nature Portfolio wishes to improve the reproducibility of the work that we publish. This form provides structure for consistency and transparency in reporting. For further information on Nature Portfolio policies, see our Editorial Policies and the Editorial Policy Checklist.

Statistics

For all statistical analyses, confirm that the following items are present in the figure legend, table legend, main text, or Methods section.

n/a	Confirmed	
☐	☒	The exact sample size (*n*) for each experimental group/condition, given as a discrete number and unit of measurement
☐	☒	A statement on whether measurements were taken from distinct samples or whether the same sample was measured repeatedly
☒	☐	The statistical test(s) used AND whether they are one- or two-sided<br>*Only common tests should be described solely by name; describe more complex techniques in the Methods section.*
☒	☐	A description of all covariates tested
☐	☒	A description of any assumptions or corrections, such as tests of normality and adjustment for multiple comparisons
☐	☒	A full description of the statistical parameters including central tendency (e.g. means) or other basic estimates (e.g. regression coefficient) AND variation (e.g. standard deviation) or associated estimates of uncertainty (e.g. confidence intervals)
☐	☒	For null hypothesis testing, the test statistic (e.g. *F*, *t*, *r*) with confidence intervals, effect sizes, degrees of freedom and *P* value noted<br>*Give P values as exact values whenever suitable.*
☒	☐	For Bayesian analysis, information on the choice of priors and Markov chain Monte Carlo settings
☒	☐	For hierarchical and complex designs, identification of the appropriate level for tests and full reporting of outcomes
☐	☒	Estimates of effect sizes (e.g. Cohen's *d*, Pearson's *r*), indicating how they were calculated

Our web collection on statistics for biologists contains articles on many of the points above.

Software and code

Policy information about availability of computer code

Data collection	Bulk RNA-seq datasets from publicly available data were retrieved from NCBI SRA (accession number: PRJNA831613, PRJNA1048297, PRJNA450300, and PRJNA253977) using SRA toolkit (v3.0.0).
Data analysis	All bioinformatics analyses used publicly available software, including:<br>SMRT Link software tools (v12.0), lima (v2.6.33), IsoSeq3 (v3.8.1), Pigeon (v1.0.0), Seurat (v5.0.1), Harmony (v1.0.1), STutility (v1.1.1), hdWGCNA (v0.3.01), g:profiler (v0.2.3), Monocle 3 (v.1.3.4), CytoTRACE (v0.3.3), GffCompare (v0.12.6), AGAT (v1.0), CPAT (v3.0.4), LncFinder (v1.1.6), PlantLncBoost (v1.0), EviAnn (v2.0.0), fastp (v0.23.4), STAR aligner (v2.7.10b), HTseq-count (v2.0.2), TO-GCN (v1.0), Cytoscape (v3.10.1), FIMO (v5.5.6), Trimmomatic (v0.39), Bowtie2 (v2.3.4.1), MACS (v2.2.7.1 & v3.0.4), MEME-ChIP (v5.5.9), ChIPseeker (v1.38.0).

For manuscripts utilizing custom algorithms or software that are central to the research but not yet described in published literature, software must be made available to editors and reviewers. We strongly encourage code deposition in a community repository (e.g. GitHub). See the Nature Portfolio guidelines for submitting code & software for further information.

Data

Policy information about availability of data

All manuscripts must include a data availability statement. This statement should provide the following information, where applicable:

- Accession codes, unique identifiers, or web links for publicly available datasets
- A description of any restrictions on data availability
- For clinical datasets or third party data, please ensure that the statement adheres to our policy

The source codes of all the analyses used in this study are publicly available and can be accessed at GitHub at https://github.com/danielylup/Maize_SpMASIsoSeq. The long-read transcript sequence dataset of MAS-IsoSeq can be downloaded from NCBI SRA with the accession number PRJNA1214004 (Both VR03 and VR01). The short-read 10x Genomics spatial transcriptomics dataset can be retrieved from the SRA and Gene Expression Omnibus (GEO) repositories using the accession numbers SRR17981909 (VR03) and SRR17968079 (VR01). Related images and processed spatial transcriptomics datasets were deposited in Gene Expression Omnibus (GEO) repositories using the accession numbers GSM5903593 (VR03) and GSM5903595 (VR01). Both the MaizeV5_IsoAnn gene-model database (https://doi.org/10.5281/zenodo.16837070) and EviAnn annotated gene models (https://doi.org/10.5281/zenodo.16868057) were uploaded to the Zenodo repository and can be retrieve using the provided DOI.

Research involving human participants, their data, or biological material

Policy information about studies with human participants or human data. See also policy information about sex, gender (identity/presentation), and sexual orientation and race, ethnicity and racism.

Reporting on sex and gender	Not applicable
Reporting on race, ethnicity, or other socially relevant groupings	Not applicable
Population characteristics	Not applicable
Recruitment	Not applicable
Ethics oversight	Not applicable

Note that full information on the approval of the study protocol must also be provided in the manuscript.

Field-specific reporting

Please select the one below that is the best fit for your research. If you are not sure, read the appropriate sections before making your selection.

☒ Life sciences ☐ Behavioural & social sciences ☐ Ecological, evolutionary & environmental sciences

For a reference copy of the document with all sections, see nature.com/documents/nr-reporting-summary-flat.pdf

Life sciences study design

All studies must disclose on these points even when the disclosure is negative.

Sample size	One biological sample (VR03) were used for the initial analysis and an additional biological sample (VR01) with similar biological condition were included for verification.
Data exclusions	To optimize resolution and minimize compositional noise for the analysis of the C4 photosynthetic developmental gradient, spatial spots corresponding to the non-photosynthetic protective sheath (coleoptile) were computationally removed prior to dimensionality reduction and unsupervised clustering. This strategic exclusion enabled high-fidelity annotation and differential expression analysis specifically within the nascent embryonic leaf primordia and associated vascular networks.
Replication	We compare the overall gene expression between the two biological replicates (VR03 and VR01), and we found the gene expression profile between the two replicates were highly correlated (Pearson's $R = 0.958$, $P < 0.01$).<br>Padlock-probe RCA in situ hybridization were performed twice independently for each probe and obtained a similar results.
Randomization	Not relevant in this study.
Blinding	Not relevant in this study.

Reporting for specific materials, systems and methods

We require information from authors about some types of materials, experimental systems and methods used in many studies. Here, indicate whether each material, system or method listed is relevant to your study. If you are not sure if a list item applies to your research, read the appropriate section before selecting a response.

Materials & experimental systems

n/a	Involved in the study	
☒	☐	Antibodies
☒	☐	Eukaryotic cell lines
☒	☐	Palaeontology and archaeology
☒	☐	Animals and other organisms
☒	☐	Clinical data
☒	☐	Dual use research of concern
☐	☒	Plants

Methods

n/a	Involved in the study	
☒	☐	ChIP-seq
☒	☐	Flow cytometry
☒	☐	MRI-based neuroimaging

Dual use research of concern

Policy information about dual use research of concern

Hazards

Could the accidental, deliberate or reckless misuse of agents or technologies generated in the work, or the application of information presented in the manuscript, pose a threat to:

No	Yes	
☒	☐	Public health
☒	☐	National security
☒	☐	Crops and/or livestock
☒	☐	Ecosystems
☒	☐	Any other significant area

Experiments of concern

Does the work involve any of these experiments of concern:

No	Yes	
☒	☐	Demonstrate how to render a vaccine ineffective
☒	☐	Confer resistance to therapeutically useful antibiotics or antiviral agents
☒	☐	Enhance the virulence of a pathogen or render a nonpathogen virulent
☒	☐	Increase transmissibility of a pathogen
☒	☐	Alter the host range of a pathogen
☒	☐	Enable evasion of diagnostic/detection modalities
☒	☐	Enable the weaponization of a biological agent or toxin
☒	☐	Any other potentially harmful combination of experiments and agents

Plants

Seed stocks	Zea mays White Crystal seedling from Taiwan was used in this study.
Novel plant genotypes	No novel plant genotype produced in this study.
Authentication	Not applicable.
