# Supplementary material for: Profiling maize embryonic leaf development and discovering new genes using high-resolution spatial long-read isoform sequencing
Source: Nat Plants. 2026 Aug 17;12(9):1860–78. doi: 10.1038/s41477-026-02364-y (PMC13585551; doi:10.1038/s41477-026-02364-y)
Supplement: Supplementary file 3 [file 41477_2026_2364_MOESM3_ESM.pdf]

nature portfolio

Peer Review File

Profiling maize embryonic leaf development and discovering new genes by high-resolution spatial long-read isoform sequencing

Corresponding Author: Dr Mei-yeh Lu

Version 0:

Reviewer comments:

Reviewer #1

(Comments for the Author)
To our knowledge, this study is the first report integrating spatial transcriptomics with full-length isoform sequencing in plants. The authors generate high-quality, well-sequenced resources for embryonic maize leaf tissue. I appreciate the inclusion and careful comparison of bulk RNA-seq, 10x Genomics 3' short-read sequencing, and long-read sequencing, and the bioinformatic analyses are insightful and well presented.
However, to strengthen the biological insights, integration with chromatin and epigenome datasets, and/or with proteomics data would be more valuable. Long-read isoform transcriptome sequencing has already been reported in human, mouse, and Arabidopsis, which limits the novelty of the methodology. For example, Patowary et al. (2024) explored isoform diversity in human brain, incorporating ATAC-Seq data with novel isoforms, and validated predicted novel exons by RT-PCR and ORFs were confirmed by MS/MS. In the Arabidopsis work, DNA methylation analysis was linked with transcriptomic regulation.
In general, I think this present work is beneficial to bioinformatics and omics community, but additional functional validation would increase its impact for the field of plant biology. It would greatly improve the manuscript if the authors could include complementary biological data or integrative analyses that link isoform diversity to chromatin states and/ or translation.

I have a few detailed questions as below.

1. Though this work used oligo (dT) based capture to enrich polyadenylated RNA during RNASeq library preparation, the libraries can still contain partially spliced pre-mRNA and noncoding RNA. The manuscript should describe precisely the criteria used in defining 'novel isoform'. For example, are these isoforms full-length, detected in multiple replicates libraries, and mapped uniquely to the locus? Could some of them be a result of incomplete splicing/ retained introns- comparisons to proteomic databases could address this.

2. In this work, the discovery of novel 3' exons that enhances short-read alignment is highlighted. However, the 3' exons could be different kinds, including the 3' UTR only, or terminal exons that include CDS with an extended 3'UTR. To report it clearly, the poly (A) sites and whether the terminal exons are included should be reported.

3. Line 104 – "identified ~7,000 previously unrecorded genes"- is there evidence these are translated, and/ or encode proteins with known domains?

4. Lines 210 to 214, without any biological information, it's too strong to conclude "the isoform-level transcriptome provides superior resolution to distinguish fine developmental stages within the embryonic vein lineage."

5. Lines 271 to 273. "This analysis identified 15 distinct co-expression modules (Fig. 4a and Supplementary Table 10) from 17,722 isoforms, with the remaining isoforms unassigned due to low expression or unique patterns." It's unclear on the threshold used in 'low expression', and what are the 'unique patterns'?

6. Abstract "Moreover, we identified LBD26 as a key transcription factor in maize embryonic vein development", as well as lines 448 to 449, "This result strongly supports a role for LBD26 in the development of maize embryonic veins." is inappropriate. To ascertain the role of LBD26, functional analysis is required, for example mutant analysis. Given the abundance of putative regulatory elements that transcriptional factors could bind to, promoter sequence similarity alone is insufficient to support ARF4 binding.

7. Line 457, how was 72.87% calculated?

8. A discussion of the limitation of this work should be included.

9. In Fig. 3A, the resolution is too low, and the SAM is obscured by the “SAM” label. In Fig. 3bc, the supervised clustering of leaf tissue spots at the gene (b) and isoform (c) levels are not clear. The colors used in the tissue map are confused and not distinguishable.

10. In Fig. 6, did the authors check for distal CNSs, which are very prevalent in maize, or for variation in LBD26 CNSs in different maize varieties?

Reviewer #2

(Comments for the Author)
This study presents an integration of long-read isoform sequencing (MAS-IsoSeq) with spatial transcriptomics to profile maize embryonic leaf development. The authors generate an isoform-annotated gene model database (MaizeV5_IsoAnn), uncover numerous novel isoforms and genes, and highlight isoform-level regulatory insights into embryonic leaf development. The resulting resource could be valuable to the maize community, and the methodological framework could be broadly applicable across plant systems. While the technical advance is clear, several biological interpretations are overstated, and additional validation is needed.

Major:

1. The claim of discovering >70% unannotated isoforms and 5,228 novel genes is significant but requires stronger validation. To confirm these findings are not artifacts, RT-PCR/qPCR confirmation of selected isoforms, proteomic evidence of translation, or in situ hybridization for spatially restricted isoforms would strengthen confidence. Additionally, corroborating the 10x spatial results with MAS-IsoSeq data from bulk samples of the same tissue would help curate the findings.

2. A large fraction of novel isoforms and genes are predicted to be non-coding. The manuscript currently provides limited exploration of their possible roles (e.g., lncRNA-mediated regulation). Without such analysis, the biological implications remain underdeveloped.

3. The functional consequences of the isoform-level differences highlighted in the manuscript, such as for WAT1-RELATED, are speculative and require experimental support. The biological impact would be more convincing with additional analyses, such as assessments of domain retention/loss across a broader set of isoforms, protein stability predictions, or co-expression with downstream targets.

4. The inference from network analysis, identifying LBD26 as a key transcription factor, relies largely on co-expression. Experimental validation - such as mutant analysis, reporter lines, or ChIP assays - is necessary. Without functional assays, the conclusion should be moderated to describe LBD26 as a "candidate regulator."

5. The pseudo-time trajectory results are presented in great technical detail but lack biological insights. The authors should streamline these sections, emphasizing developmental implications (e.g., Kranz anatomy formation, vascular patterning) rather than computational procedures.

6. The resolution of the 10x spatial platform is relatively low and captures multiple cells in a spot, potentially mixing signals from neighboring tissues. This limitation should be acknowledged more explicitly, especially when drawing conclusions about isoform-level regulation in narrow structures such as veins.

Minor:

1. Terms such as “isoform,” “transcript,” and “gene model” are sometimes used interchangeably, which may confuse readers. Consistent definitions should be maintained.

2. Several enrichment and marker analyses are described as “significant” with adjusted p-values < 0.05 (lines 220 and 657), but the correction method (e.g., Benjamini–Hochberg, Bonferroni) is not always stated. Clarifying this would enhance transparency.

Decision Letter:

20th October 2025

Dear Dr Lu,

Your Resource, "Profiling maize embryonic leaf development and discovering new genes by high-resolution spatial long-read isoform sequencing" has now been seen by two referees. You will see from their comments below that while they find your work of interest, some important points are raised. We are interested in the possibility of publishing your study in Nature Plants, but would like to consider your response to these concerns in the form of a revised manuscript before we make a final decision on publication.

As you can read below, both reviewers think that the Resource will be valuable for the community, however, they raise some concerns that require revision. In our opinion the main points are:

1. Reviewer #1 thinks that it would greatly improve the manuscript if the authors could include complementary biological data or integrative analyses that link isoform diversity to chromatin states and/or translation.
2. Both reviewers #1 and #2 think that novel isoforms should be analyzed and described in more detail. Specifically, reviewer #1 states that novel isoforms and 3' exons should be described better, and reviewer #2 thinks that the potential functions of novel non-coding isoforms should be analyzed and discussed.
3. Reviewer #2 states that the claim of discovering >70% unannotated isoforms and 5,228 novel genes requires stronger validation. Furthermore, functional consequences of isoform-level differences requires further experimental support.

All referees also raise some other points that should be addressed. As usual, please do not hesitate to get in touch if you would like to discuss these issues further.

We therefore invite you to revise your manuscript taking into account all reviewer and editor comments. Please highlight all changes in the manuscript text file, preferably in Microsoft Word format.

We are committed to providing a fair and constructive peer-review process. Do not hesitate to contact us if there are specific requests from the reviewers that you believe are technically impossible or unlikely to yield a meaningful outcome.

When revising your manuscript please:

* Include a "Response to referees" document detailing, point-by-point, how you addressed each referee comment. If no action was taken to address a point, you must provide a compelling argument. This response will be sent back to the referees along with the revised manuscript.

* If you have not done so already please begin to revise your manuscript so that it conforms to our Resource format instructions at http://www.nature.com/nplants/info/final-submission. Refer also to any guidelines provided in this letter.

* Include a revised version of any required reporting checklist. It will be available to referees (and, potentially, statisticians) to aid in their evaluation if the manuscript goes back for peer review. A revised checklist is essential for re-review of the paper.

* Pay close attention to our href="https://www.nature.com/nature-portfolio/editorial-policies/image-integrity">Digital Image Integrity Guidelines. and to the following points. Please ensure:
-- that unprocessed scans are clearly labelled and match the gels and western blots presented in figures.
-- that control panels for gels and western blots are appropriately described as loading on sample processing controls
-- that all images in the paper are checked for duplication of panels and for splicing of gel lanes.
-- that you retain unprocessed data and metadata files after publication, ideally archiving data in perpetuity. These may be requested during the peer review and production process or after publication if any issues arise.

EXTENDED DATA FIGURES

When re-submitting your manuscript, please ensure that any supplementary figures and tables that are crucial to the manuscript's conclusions are converted into Extended Data figures and tables to increase visibility of these data. Extended Data figures and tables are online-only (present in the online PDF and full-text HTML versions of the paper), peer-reviewed display items that provide essential background to the article but are not included in the main article due to space constraints. A maximum of ten Extended Data display items (figures and tables) is permitted.

Nature Plants strongly supports public availability of data and are therefore keen that the data used in your paper is placed in an appropriate public data repository. Alternatively, if this is not possible, you may present the data as Extended Data or Supplementary Information. If data can only be shared on request, please explain why in your Data Availability Statement, and also in the correspondence with your editor. Please note that for some data types, deposition in a public repository is mandatory.

Please use the link below to submit your revised manuscript and related files:

Link Redacted

Note: This URL links to your confidential home page and associated information about manuscripts you may have submitted, or that you are reviewing for us. If you wish to forward this email to co-authors, please delete the link to your homepage.

We hope to receive your revised manuscript within three to four months, however, this is not a strict deadline. If you cannot send it within this time, please let us know. We will be happy to consider your revision so long as nothing similar has been accepted for publication at Nature Plants or published elsewhere.

We look forward to seeing the revised manuscript and thank you for the opportunity to review your work.

Nature Plants is committed to improving transparency in authorship. As part of our efforts in this direction, we are now requesting that all authors identified as 'corresponding author' on published papers create and link their Open Researcher and Contributor Identifier (ORCID) with their account on the Manuscript Tracking System (MTS), prior to acceptance. This applies to primary research papers only. ORCID helps the scientific community achieve unambiguous attribution of all scholarly contributions. You can create and link your ORCID from the home page of the MTS by clicking on 'Modify my Springer Nature account'. For more information please visit please visit www.springernature.com/orcid.

Please do not hesitate to contact me if you have any questions or would like to discuss these revisions further.

****

Reviewers' Comments:

Reviewer #1 (Comments for the Author):

To our knowledge, this study is the first report integrating spatial transcriptomics with full-length isoform sequencing in plants. The authors generate high-quality, well-sequenced resources for embryonic maize leaf tissue. I appreciate the inclusion and careful comparison of bulk RNA-seq, 10x Genomics 3' short-read sequencing, and long-read sequencing, and the bioinformatic analyses are insightful and well presented.
However, to strengthen the biological insights, integration with chromatin and epigenome datasets, and/or with proteomics data would be more valuable. Long-read isoform transcriptome sequencing has already been reported in human, mouse, and Arabidopsis, which limits the novelty of the methodology. For example, Patowary et al. (2024) explored isoform diversity in human brain, incorporating ATAC-Seq data with novel isoforms, and validated predicted novel exons by RT-PCR and ORFs were confirmed by MS/MS. In the Arabidopsis work, DNA methylation analysis was linked with transcriptomic regulation.
In general, I think this present work is beneficial to bioinformatics and omics community, but additional functional validation would increase its impact for the field of plant biology. It would greatly improve the manuscript if the authors could include complementary biological data or integrative analyses that link isoform diversity to chromatin states and/ or translation.

I have a few detailed questions as below.

1. Though this work used oligo (dT) based capture to enrich polyadenylated RNA during RNASeq library preparation, the libraries can still contain partially spliced pre-mRNA and noncoding RNA. The manuscript should describe precisely the criteria used in defining 'novel isoform'. For example, are these isoforms full-length, detected in multiple replicates libraries, and mapped uniquely to the locus? Could some of them be a result of incomplete splicing/ retained introns- comparisons to proteomic databases could address this.

2. In this work, the discovery of novel 3' exons that enhances short-read alignment is highlighted. However, the 3' exons could be different kinds, including the 3' UTR only, or terminal exons that include CDS with an extended 3'UTR. To report it clearly, the poly (A) sites and whether the terminal exons are included should be reported.

3. Line 104 – "identified ~7,000 previously unrecorded genes"- is there evidence these are translated, and/ or encode proteins with known domains?

4. Lines 210 to 214, without any biological information, it's too strong to conclude "the isoform-level transcriptome provides superior resolution to distinguish fine developmental stages within the embryonic vein lineage."

5. Lines 271 to 273. "This analysis identified 15 distinct co-expression modules (Fig. 4a and Supplementary Table 10) from 17,722 isoforms, with the remaining isoforms unassigned due to low expression or unique patterns." It's unclear on the threshold used in 'low expression', and what are the 'unique patterns'?

6. Abstract "Moreover, we identified LBD26 as a key transcription factor in maize embryonic vein development", as well as lines 448 to 449, "This result strongly supports a role for LBD26 in the development of maize embryonic veins." is inappropriate. To ascertain the role of LBD26, functional analysis is required, for example mutant analysis. Given the abundance of putative regulatory elements that transcriptional factors could bind to, promoter sequence similarity alone is insufficient to support ARF4 binding.

7. Line 457, how was 72.87% calculated?

8. A discussion of the limitation of this work should be included.

9. In Fig. 3A, the resolution is too low, and the SAM is obscured by the "SAM" label. In Fig. 3bc, the supervised clustering of leaf tissue spots at the gene (b) and isoform (c) levels are not clear. The colors used in the tissue map are confused and not distinguishable.

10. In Fig. 6, did the authors check for distal CNSs, which are very prevalent in maize, or for variation in LBD26 CNSs in different maize varieties?

Reviewer #2 (Comments for the Author):

This study presents an integration of long-read isoform sequencing (MAS-IsoSeq) with spatial transcriptomics to profile maize embryonic leaf development. The authors generate an isoform-annotated gene model database (MaizeV5_IsoAnn), uncover numerous novel isoforms and genes, and highlight isoform-level regulatory insights into embryonic leaf development. The resulting resource could be valuable to the maize community, and the methodological framework could be broadly applicable across plant systems. While the technical advance is clear, several biological interpretations are overstated, and additional validation is needed.

Major:

1. The claim of discovering >70% unannotated isoforms and 5,228 novel genes is significant but requires stronger validation. To confirm these findings are not artifacts, RT-PCR/qPCR confirmation of selected isoforms, proteomic evidence of translation, or in situ hybridization for spatially restricted isoforms would strengthen confidence. Additionally, corroborating the 10x spatial results with MAS-IsoSeq data from bulk samples of the same tissue would help curate the findings.

2. A large fraction of novel isoforms and genes are predicted to be non-coding. The manuscript currently provides limited exploration of their possible roles (e.g., lncRNA-mediated regulation). Without such analysis, the biological implications remain underdeveloped.

3. The functional consequences of the isoform-level differences highlighted in the manuscript, such as for WAT1-RELATED, are speculative and require experimental support. The biological impact would be more convincing with additional analyses, such as assessments of domain retention/loss across a broader set of isoforms, protein stability predictions, or co-expression with downstream targets.

4. The inference from network analysis, identifying LBD26 as a key transcription factor, relies largely on co-expression. Experimental validation - such as mutant analysis, reporter lines, or ChIP assays - is necessary. Without functional assays, the conclusion should be moderated to describe LBD26 as a "candidate regulator."

5. The pseudo-time trajectory results are presented in great technical detail but lack biological insights. The authors should streamline these sections, emphasizing developmental implications (e.g., Kranz anatomy formation, vascular patterning) rather than computational procedures.

6. The resolution of the 10x spatial platform is relatively low and captures multiple cells in a spot, potentially mixing signals from neighboring tissues. This limitation should be acknowledged more explicitly, especially when drawing conclusions about isoform-level regulation in narrow structures such as veins.

Minor:

1. Terms such as “isoform,” “transcript,” and “gene model” are sometimes used interchangeably, which may confuse readers. Consistent definitions should be maintained.

2. Several enrichment and marker analyses are described as “significant” with adjusted p-values < 0.05 (lines 220 and 657), but the correction method (e.g., Benjamini–Hochberg, Bonferroni) is not always stated. Clarifying this would enhance transparency.

********************END********************

Version 1:

Reviewer comments:

Reviewer #1

(Comments for the Author)
The authors have made a serious effort to address the previous concerns, and the new analyses improve the manuscript. I appreciate the added ATAC-seq integration, independent MAS-IsoSeq replicate, padlock-RCA in situ validation, and DAP-seq experiment. I have several remaining comments.

1. ATAC-seq integration
The ATAC-seq analysis is informative for linking alternative TSS-associated isoforms with chromatin accessibility. However, it should avoid describing as direct validation of novel isoforms or TSS usage. It is more appropriate as regulatory context.

2. Novel transcriptome validation

The poly-A motif screening, coding-potential prediction, PFAM scanning, and AlphaFold/stability analyses are useful, but they are computational annotations. In particular, there is no annotation of protein coding potential specifically of the new potential domains detected in novel isoforms. They do not directly demonstrate translation or protein function, so it should not be described as validation experiments.

3. Padlock-RCA in situ hybridization
The padlock-RCA experiment is a valuable addition for selected candidates. Since this assay generally detects specific junctions or short isoform-specific regions, it should not be presented as confirming full-length novel isoforms. The probe design, target regions, controls, and signal quantification should be clearly described.

4. DAP-seq for LBD26
DAP-seq supports the DNA-binding capacity of LBD26 and identifies candidate binding sites. However, as an in vitro assay, it does not establish in vivo binding or functional regulation. Genes such as APC10 should therefore be described as candidate or putative targets unless additional supporting evidence is provided. The lack of mutant analysis remains a major limitation of this largely computational study.

5. In the Fig. 4a–b, I think the current leaf histology image is still difficult to interpret. The tissue domains and boundary is hard to distinguish. In addition, several cross-sectional histology images in Extended Data Figs. 2, 3, and 7, and supplementary figures, are also blurry, making tissue boundaries difficult to assess. Higher-resolution images, clearer color contrast, or enlarged insets for key regions may improve readability.

Overall, the revision is stronger than the original submission, but these points should be addressed to improve precision and figure readability.

Reviewer #2

(Comments for the Author)
The authors provided an extensive revision of the manuscript, and had answered all my earlier questions.

Decision Letter:

Our ref: NPLANTS-250920790A

1st June 2026

Dear Dr. Lu,

Thank you for submitting your revised manuscript "Profiling maize embryonic leaf development and discovering new genes by high-resolution spatial long-read isoform sequencing" (NPLANTS-250920790A). It has now been seen by the original referees and their comments are below. The reviewers find that the paper has improved in revision, and therefore we'll be happy in principle to publish it in Nature Plants, pending minor revisions to satisfy the referees' final requests and to comply with our editorial and formatting guidelines.

We are now performing detailed checks on your paper and will send you a checklist detailing our editorial and formatting requirements in about a week. Please do not upload the final materials and make any revisions until you receive this additional information from us.

Thank you again for your interest in Nature Plants Please do not hesitate to contact me if you have any questions.

Sincerely,

Reviewer #1 (Comments for the Author):

The authors have made a serious effort to address the previous concerns, and the new analyses improve the manuscript. I appreciate the added ATAC-seq integration, independent MAS-IsoSeq replicate, padlock-RCA in situ validation, and DAP-seq experiment. I have several remaining comments.

1. ATAC-seq integration
The ATAC-seq analysis is informative for linking alternative TSS-associated isoforms with chromatin accessibility. However, it should avoid describing as direct validation of novel isoforms or TSS usage. It is more appropriate as regulatory context.

2. Novel transcriptome validation
The poly-A motif screening, coding-potential prediction, PFAM scanning, and AlphaFold/stability analyses are useful, but they are computational annotations. In particular, there is no annotation of protein coding potential specifically of the new potential domains detected in novel isoforms. They do not directly demonstrate translation or protein function, so it should not be

described as validation experiments.

3. Padlock-RCA in situ hybridization
The padlock-RCA experiment is a valuable addition for selected candidates. Since this assay generally detects specific junctions or short isoform-specific regions, it should not be presented as confirming full-length novel isoforms. The probe design, target regions, controls, and signal quantification should be clearly described.

4. DAP-seq for LBD26
DAP-seq supports the DNA-binding capacity of LBD26 and identifies candidate binding sites. However, as an in vitro assay, it does not establish in vivo binding or functional regulation. Genes such as APC10 should therefore be described as candidate or putative targets unless additional supporting evidence is provided. The lack of mutant analysis remains a major limitation of this largely computational study.

5. In the Fig. 4a–b, I think the current leaf histology image is still difficult to interpret. The tissue domains and boundary is hard to distinguish. In addition, several cross-sectional histology images in Extended Data Figs. 2, 3, and 7, and supplementary figures, are also blurry, making tissue boundaries difficult to assess. Higher-resolution images, clearer color contrast, or enlarged insets for key regions may improve readability.

Overall, the revision is stronger than the original submission, but these points should be addressed to improve precision and figure readability.

Reviewer #2 (Comments for the Author):

The authors provided an extensive revision of the manuscript, and had answered all my earlier questions.

Version 2:

Decision Letter:

10th July 2026

Dear Dr Lu,

We are pleased to inform you that your Resource entitled "Profiling maize embryonic leaf development and discovering new genes by high-resolution spatial long-read isoform sequencing", has now been accepted for publication in Nature Plants.

Over the next few weeks, your paper will be copyedited to ensure that it conforms to Nature Plants style. We look particularly carefully at the titles of all papers to ensure that they are relatively brief and understandable.

Once your paper is typeset, you will receive an email with a link to choose the appropriate publishing options for your paper and our Author Services team will be in touch regarding any additional information that may be required.

After the grant of rights is completed, you will receive a link to your electronic proof via email with a request to make any corrections within 48 hours. If, when you receive your proof, you cannot meet this deadline, please inform us at rjsproduction@springernature.com immediately.

You will not receive your proofs until the publishing agreement has been received through our system.

You may wish to make your media relations office aware of your accepted publication, in case they consider it appropriate to organize some internal or external publicity. Once your paper has been scheduled you will receive an email confirming the publication details. This is normally 3-4 working days in advance of publication. If you need additional notice of the date and time of publication, please let the production team know when you receive the proof of your article to ensure there is sufficient time to coordinate. Further information on our embargo policies can be found here:
https://www.nature.com/authors/policies/embargo.html

Due to the importance of these deadlines, we ask you please us know now whether you will be difficult to contact over the next month. If this is the case, we ask you provide us with the contact information (email, phone and fax) of someone who will be able to check the proofs on your behalf, and who will be available to address any last-minute problems.

Acceptance of your manuscript is conditional on all authors' agreement with our publication policies (see http://www.nature.com/authors/policies/index.html). In particular your manuscript must not be published elsewhere.

If you have any questions about our publishing options, costs, Open Access requirements, or our legal forms, please contact

ASJournals@springernature.com

Authors may need to take specific actions to achieve compliance with funder and institutional open access mandates. If your research is supported by a funder that requires immediate open access (e.g. according to Plan S principles or the NIH public access policy) then you should select the gold OA route, and we will direct you to the compliant route where possible. Because authors warrant under our subscription licensing terms that they haven't committed to licensing any version of their article under a licence inconsistent with the terms of our agreement – including the applicable embargo period – publication under the subscription model isn't suitable for authors whose funders require no embargo.

An online order form for reprints of your paper is available at https://www.nature.com/reprints/author-reprints.html. All co-authors, authors' institutions and authors' funding agencies can order reprints using the form appropriate to their geographical region.

We welcome the submission of potential cover material (including a short caption of around 40 words) related to your manuscript; suggestions should be sent to Nature Plants as electronic files (the image should be 300 dpi at 210 x 297 mm in either TIFF or JPEG format). Please note that such pictures should be selected more for their aesthetic appeal than for their scientific content, and that colour images work better than black and white or grayscale images. Please include a written description of your image and how it was created along with your image files. Please do not try to design a cover with the Nature Plants logo etc., and please do not submit composites of images related to your work. I am sure you will understand that we cannot make any promise as to whether any of your suggestions might be selected for the cover of the journal.

You can now use a single sign-on for all your accounts, view the status of all your manuscript submissions and reviews, access usage statistics for your published articles and download a record of your refereeing activity for the Nature journals.

To assist our authors in disseminating their research to the broader community, our SharedIt initiative provides you with a unique shareable link that will allow anyone (with or without a subscription) to read the published article. Recipients of the link with a subscription will also be able to download and print the PDF.

As soon as your article is published, you will receive an automated email with your shareable link.

With kind regards,

P.S. Click on the following link if you would like to recommend Nature Plants to your librarian
http://www.nature.com/subscriptions/recommend.html#forms

** Visit the Springer Nature Editorial and Publishing website at www.springernature.com/editorial-and-publishing-jobs for more information about our career opportunities. If you have any questions please click here.**

Open Access This Peer Review File is licensed under a Creative Commons Attribution 4.0 International License, which permits use, sharing, adaptation, distribution and reproduction in any medium or format, as long as you give appropriate credit to the original author(s) and the source, provide a link to the Creative Commons license, and indicate if changes were made. In cases where reviewers are anonymous, credit should be given to 'Anonymous Referee' and the source.
The images or other third party material in this Peer Review File are included in the article's Creative Commons license, unless indicated otherwise in a credit line to the material. If material is not included in the article's Creative Commons license and your intended use is not permitted by statutory regulation or exceeds the permitted use, you will need to obtain permission directly from the copyright holder.
To view a copy of this license, visit https://creativecommons.org/licenses/by/4.0/

Responses to reviewers' comments:

We are grateful to the two reviewers for their valuable comments and suggestions. We have tried our best to respond to their comments and revised our manuscript accordingly (see the track-change version).

To strengthen the biological depth and technical robustness of our work, we have conducted the following additional experiments and integrative analyses:

- **Integrative analysis of maize ATAC-seq datasets:** We associated identified alternative transcription start sites with specific chromatin accessibility states to provide regulatory context.
- **Extensive validation of novel isoforms:** We conducted a systematic screening for canonical poly-A motifs and performed protein-coding potential assessments and PFAM domain scanning for all novel isoforms.
- **Functional and structural stability analysis:** We utilized AlphaFold3 to predict protein structures and compared the thermostability (ΔG) between novel and canonical isoforms.
- **Independent biological replicate for MAS-IsoSeq:** We provided validation for the >70% unannotated isoforms and 5,228 novel genes, demonstrating the high reproducibility of our pipeline.
- **Padlock-based Rolling Circle Amplification (RCA) *in situ* hybridization:** We confirmed the physical presence and spatial patterning of selected genes and their novel isoforms within embryonic leaf tissues.
- **DNA affinity purification sequencing (DAP-seq):** We provided direct experimental evidence for the binding motifs of the LBD26 protein and its target genes, such as *APC10*.

Below are our point-by-point responses to their comments.

Reviewer #1 (Comments for the Author):

To our knowledge, this study is the first report integrating spatial transcriptomics with full-length isoform sequencing in plants. The authors generate high-quality, well-sequenced resources for embryonic maize leaf tissue. I appreciate the inclusion and careful comparison of bulk RNA-seq, 10x Genomics 3' short-read sequencing, and long-read sequencing, and the bioinformatic analyses are insightful and well presented.
However, to strengthen the biological insights, integration with chromatin and epigenome

datasets, and/or with proteomics data would be more valuable. Long-read isoform transcriptome sequencing has already been reported in human, mouse, and Arabidopsis, which limits the novelty of the methodology. For example, Patowary et al. (2024) explored isoform diversity in human brain, incorporating ATAC-Seq data with novel isoforms, and validated predicted novel exons by RT-PCR and ORFs were confirmed by MS/MS. In the Arabidopsis work, DNA methylation analysis was linked with transcriptomic regulation.
In general, I think this present work is beneficial to bioinformatics and omics community, but additional functional validation would increase its impact for the field of plant biology. It would greatly improve the manuscript if the authors could include complementary biological data or integrative analyses that link isoform diversity to chromatin states and/ or translation.

REPLY:

Your positive comments are greatly appreciated. In response to the suggestions to “integrate with chromatin and epigenome datasets, and/or with proteomics data” and “include complementary biological data or integrative analysis that link isoform diversity to chromatin states and/or translation”, we have conducted the following tasks: (1) We integrated publicly available maize ATAC-seq dataset (Liu et al. 2025)[1] into our study to validate novel isoform with alternative transcription start site. (2) We strengthened our findings by adding an independent MAS-IsoSeq biological replicate from similar developmental stages and tissue types. (3) To provide functional validation, we have evaluated all putative novel isoforms for protein-coding potential and scanned for PFAM domains. (4) To further support the role of *LBD26* as a candidate regulator in embryonic vein development, we performed DAP-seq to identify its specific binding motifs (PWMs) and binding sites on the promoter regions. Thanks to your suggestions, these additional analyses should substantially enhance the impact of this manuscript on the field of plant biology.

I have a few detailed questions as below.

1. Though this work used oligo (dT) based capture to enrich polyadenylated RNA during RNASeq library preparation, the libraries can still contain partially spliced pre-mRNA and noncoding RNA. The manuscript should describe precisely the criteria used in defining ‘novel isoform’. For example, are these isoforms full-length, detected in multiple replicates libraries, and mapped uniquely to the locus? Could some of them be a result of incomplete splicing/ retained introns- comparisons to proteomic databases could address this.

REPLY:

Thanks for raising the question. We defined the "novel isoform" as any isoform of known genes but not currently recorded in *Zea mays* B73 RefGen_v5 database, these specifically include

isoforms categorized as "Novel In Catalog" (NIC), "Novel Not In Catalog" (NNIC), or "Incomplete-splice Match" (ISM) in isoform classification (definitions in Supplementary Table 2). To ensure high technical confidence, all isoforms were derived from highly accurate HiFi Iso-Seq data (>Q30), with each transcript uniquely mapped to a specific genomic locus. Furthermore, the novel isoforms were retained with support by at least two reads carrying independent UMIs. The related sections are provided below:

[Page 5, lines 121-124]: We have now provided the statement as: **"**These isoforms, which fall into the structural categories of "incomplete-splice match" (ISM), "novel in catalog" (NIC), and "novel not in catalog" (NNIC), are hereafter collectively defined as "novel isoforms" throughout this study (Supplementary Table 2).**"**

[Supplementary Table 1]: While the presence of partially spliced pre-mRNA and non-coding mRNA cannot be ruled out from the in vivo capture, all the isoforms identified in this study are supported by highly accurate (>Q30) long-reads that do not require computational *de novo* assembly.

[Page 5, Lines 113-114]: Furthermore, all these 38,543,204 long-reads were mapped uniquely to their corresponding genomic locations.

[Supplementary Table 4]: Moreover, each identified isoform is supported by at least 2 independent transcripts with different UMIs.

[Page 7, Lines 186-193]: To examine the possibility of the novel isoforms being incomplete splicing or retained intron, we compared the amino acid sequences of all inferred novel isoforms with the PFAM database, and identified 104,482 novel isoforms (49.80%) containing known PFAM domains. Furthermore, when compared to the functional domains of their canonical counterpart, 74.43% (intron retention) and 90.11% (novel splice sites) of these novel isoforms retained the same domains.

2. In this work, the discovery of novel 3' exons that enhances short-read alignment is highlighted. However, the 3' exons could be different kinds, including the 3' UTR only, or terminal exons that include CDS with an extended 3'UTR. To report it clearly, the poly (A) sites and whether the terminal exons are included should be reported.

REPLY:

Thanks for the good suggestion. We screened for the canonical poly-A sites in the 3'-exons of the isoforms.

[Page 8, lines 194-202]: Among the novel isoforms identified in this study, we identified 5,445 contain at least one novel 3' exon. Our analysis further revealed that 190 of these novel 3' exon include both coding sequences (CDS) and 3' untranslated regions (UTR). Furthermore, we screened for poly-A motifs along these novel 3' exons and found that 23.65 % of them

contains these poly-A motifs.

3. Line 104 – "identified ~7,000 previously unrecorded genes"- is there evidence these are translated, and/ or encode proteins with known domains?

REPLY:

[Pages 16-17, lines 478-487 & Extended Data Fig. 4a]: By comparing with the PFAM database, there are 679 out of 5,228 novel genes (12.99%) that contained known protein domains. Additionally, our protein-coding potential predictions indicate that 24.92% of the novel genes have high protein coding potentials.

4. Lines 210 to 214, without any biological information, it's too strong to conclude "the isoform-level transcriptome provides superior resolution to distinguish fine developmental stages within the embryonic vein lineage."

REPLY:

[Page 11, lines 294-297]: Thanks for the comment. We have now rephrased this sentence to tone down our statement. The revised sentence now reads: "Thus, these findings indicate that an isoform-level transcriptome may offer the sensitivity required to resolve intricate developmental transitions within the embryonic vein lineage that might be overlooked at the gene level."

5. Lines 271 to 273. "This analysis identified 15 distinct co-expression modules (Fig. 4a and Supplementary Table 10) from 17,722 isoforms, with the remaining isoforms unassigned due to low expression or unique patterns." It's unclear on the threshold used in 'low expression', and what are the 'unique patterns'?

REPLY:

Thanks for the comment. We made clarifications in revised manuscript accordingly.

[Page 14, lines 391-394]: "Low expression" refers to a large majority of the spatial spots showing zero expression, with the detection threshold determined by the WGCNA algorithm. The term "unique pattern" describes isoform that exhibit low defining co-expression module assignment of isoforms.

6. Abstract "Moreover, we identified LBD26 as a key transcription factor in maize embryonic vein development", as well as lines 448 to 449, "This result strongly supports a role for LBD26 in the development of maize embryonic veins." is inappropriate. To ascertain the role of LBD26, functional analysis is required, for example mutant analysis. Given the abundance of putative regulatory elements that transcriptional factors could bind to, promoter sequence similarity

alone is insufficient to support ARF4 binding.

REPLY:

Thank you for advising us to obtain additional data to support LBD26 as a putative TF.

[Pages 20-21, lines 595-628 & Supplementary Table 22]: We agree that further evidence for ARF4 protein binding and *LBD26* function is useful. While traditional mutant analysis is currently beyond our lab's capabilities and setting because the experiment requires tremendous amounts of time. Alternatively, we performed DNA Affinity Purification Sequencing (DAP-seq) with the maize LBD26 protein to biologically validate its binding to the C4 plant promoter region of target genes, including *APC10* that is involved in xylem and phloem histogenesis.

[Page 2, line 32 & Page 21, lines 626-628]: Based on the DAP-seq data and the findings, we have rephrased and toned down the statement "strongly support a role for *LBD26* in the development of maize embryonic vein" to avoid overclaiming. The revised sentence now reads: "Collectively, these results provide compelling evidences to suggest that LBD26 plays a role in embryonic vein development."

7. Line 457, how was 72.87% calculated?

REPLY:

[Page 21, lines 637-638]: The 72.87% is the sum of the percentages of NNC (30.58%), NIC (18.92%), and ISM (23.37%) isoforms.

8. A discussion of the limitation of this work should be included.

REPLY:

[Page 25, lines 742-759]: Thank you for the suggestion. We have now provided a section addressing the limitations in the Discussion.

9. In Fig. 3A, the resolution is too low, and the SAM is obscured by the "SAM" label. In Fig. 3bc, the supervised clustering of leaf tissue spots at the gene (b) and isoform (c) levels are not clear. The colors used in the tissue map are confused and not distinguishable.

REPLY:

Thanks for pointing out the low-resolution histology image in Fig. 3a. We have replaced it with a high-definition version and moved all the tissue domain labels to the right side for better visibility. We have also revised the color palette in Fig.3b-c to improve contrast against the tissue background and added outline to each spatial spot to enhance clarity.

10. In Fig. 6, did the authors check for distal CNSs, which are very prevalent in maize, or for

variation in LBD26 CNSs in different maize varieties?

REPLY:

[Page 20, lines 589-593 & Extended Data Fig. 5c]: Thanks for the insightful suggestion on examination of distal CNS in maize. We identified two ARF4 protein binding sites located 2,615 bp upstream of the *LBD26* TSS. We acknowledge that this single instance doesn't illustrate the full landscape of distal CNSs in maize, but a comprehensive investigation would require technique such as STARR-seq or HiChIP, for which we lack the expertise and the suitable antibody. We agree this is a compelling topic that warrants dedicated independent study.

Reviewer #2 (Comments for the Author):

This study presents an integration of long-read isoform sequencing (MAS-IsoSeq) with spatial transcriptomics to profile maize embryonic leaf development. The authors generate an isoform-annotated gene model database (MaizeV5_IsoAnn), uncover numerous novel isoforms and genes, and highlight isoform-level regulatory insights into embryonic leaf development. The resulting resource could be valuable to the maize community, and the methodological framework could be broadly applicable across plant systems. While the technical advance is clear, several biological interpretations are overstated, and additional validation is needed.

REPLY:

We sincerely appreciate the reviewer's recognition that our study provides a valuable resource for the maize community and offers a methodological framework with broad applications across plant systems.

To address the concerns regarding validation and biological interpretation, we have significantly strengthened the manuscript by incorporating (1) a biological replicate (sample VR01), (2) the putative functional impacts of intron retention and novel splice sites through functional domain scanning and protein structure thermostability, and (3) identification of binding motifs of LBD26 by DNA affinity purification sequencing (DAP-seq). We also rephrased some of our sentences to improve clarity and to prevent overstatement on the biological interpretation. We believe these additions provide the necessary biological support to complement our technical advances.

Major:

1. The claim of discovering >70% unannotated isoforms and 5,228 novel genes is significant but requires stronger validation. To confirm these findings are not artifacts, RT-PCR/qPCR confirmation of selected isoforms, proteomic evidence of translation, or in situ hybridization for spatially restricted isoforms would strengthen confidence. Additionally, corroborating the 10x spatial results with MAS-IsoSeq data from bulk samples of the same tissue would help curate the findings.

REPLY:

We agree that robust validation is essential, thus have obtained the spatial Iso-Seq datasets from a biological replicate, and conducted in situ localization of selected marker genes and isoforms.

[Pages 12-13, lines 348-367 & Extended Data Fig. 2]: For biological replication, we have conducted the MAS-IsoSeq analysis of an independent biological replicate (VR01) from the same Visium spatial experiment (Wu et al. 2026)[2] following the same experimental procedure for VR03 sample. We observed that 45.61% of the isoforms identified in sample VR03 were also captured in VR01.

[Page 13, lines 368-383 & Extended Data Fig. 3]: In parallel, we have validated some of the target genes that are essential to our conclusion by performing Rolling Circle Amplification (RCA) *in situ* hybridization of 3 selected genes (*WAT1-RELATED*, *COX6a1*, and *RPL35a*), confirming their localization in consistence with the spatial expression profiling.

2. A large fraction of novel isoforms and genes are predicted to be non-coding. The manuscript currently provides limited exploration of their possible roles (e.g., lncRNA-mediated regulation). Without such analysis, the biological implications remain underdeveloped.

REPLY:

[Page 17, lines 490–496 & Supplementary Table 14]: We agree that characterizing the non-coding potential of novel isoforms and genes is essential for understanding their biological implications. To identify potential long non- coding RNAs (lncRNAs) among the novel genes, we performed a BLASTn search of all novel isoforms against the *Z. mays* lncRNA database from GreeNC. Using stringent alignment criteria (query coverage ≥ 90%, identity ≥ 90%, and E-value ≤ 1×10^{-5}), we identified 228 isoforms from 201 novel genes. Of these, 200 out of 201 novel genes were confirmed as non-coding with GreenNC hits and with no detectable PFAM domains.

[Page 17, lines 496–499 & Supplementary Table 4]: We also characterized novel isoforms from the known gene, and identified 1,178 novel isoforms that matched the GreeNC lncRNA database under the same stringent criteria. Among these, 897 isoforms were predicted to be non-coding and lacked PFAM domain hits, suggesting they may represent previously

unannotated lncRNA variants of known loci.

We recognize that lncRNA-mediated regulation is a complex process involving diverse mechanisms, such as chromatin remodeling, transcriptional interference, or acting as molecular decoys. Given this complexity, a comprehensive investigation into the specific regulatory mechanisms of these putative lncRNAs is beyond the scope of current study and remains a compelling subject for future independent research.

3. The functional consequences of the isoform-level differences highlighted in the manuscript, such as for WAT1-RELATED, are speculative and require experimental support. The biological impact would be more convincing with additional analyses, such as assessments of domain retention/loss across a broader set of isoforms, protein stability predictions, or co-expression with downstream targets.

REPLY:

[Page 10, lines 268-278 & Extended Data Fig. 1]: We have now explored the functional consequences of *WAT1-RELATED* isoforms by conducting the following analyses. (1) Examination of intron retention in the novel isoform of *WAT1-RELATED* (*WAT1-RELATED.2* and *WAT1-RELATED.3*) by scanning for PFAM domains within the predicted ORF, and the result confirmed that the EamA domains are lost due to a frameshift in ORF. (2) We further examined the thermostability of protein structure from these isoforms using AlphaFold3, and uncovered that the protein structures of the novel isoforms are relatively unstable compared to the canonical counterpart.

[Page 7, lines 186-193 & Fig. 3b]: Following this lead, we investigated whether domain loss is a universal feature of novel isoform with intron retention (IR) and novel splice sites (NSS). Interestingly, we found that a large majority of the novel isoforms tentatively retain their functional domains, among which 90.11% and 74.43 % of novel isoforms with at least one novel splice site (NSS) and intron retention (IR), respectively.

4. The inference from network analysis, identifying LBD26 as a key transcription factor, relies largely on co-expression. Experimental validation - such as mutant analysis, reporter lines, or ChIP assays - is necessary. Without functional assays, the conclusion should be moderated to describe LBD26 as a "candidate regulator."

REPLY:

[Page 20-21, lines 595-628 & Extended Data Fig. 6 & Supplementary Table 22]: Thanks for the great suggestion. To address this issue, we have performed DAP-seq using the maize LBD26 protein sequence against a C4 crop *Setaria Italica* genomic DNA library. The reason we used millet *S. italica* genome library is due to the difficulty we encountered while preparing

Z. mays genomic DNA library given the time constraint for manuscript revision. Hence, we adopted the approach successfully employed in a previous study (Liu et. al. 2020)[3], which utilized *S. italica* genome library for DAP-seq of maize's TF. The *S. italica* library should be suitable to identify significant binding motifs based on the high orthology with maize that over 80% of maize and millet transcription factors (TFs) are orthologous (Lin et al. 2014)[4], facilitating the identification of shared transcription factor binding site (TFBS). We obtained the motifs from the DAP-seq experiment, and confirmed LBD26 protein's binding at *APC10*'s promoter, a gene involved in xylem and phloem histogenesis, which coincide with our TO-GCN result.

[Page 19, line 563]: Based on the new result, we have revised our claim by referring *LBD26* as a "putative regulator" rather than a "key transcription factor" in the revised manuscript.

5. The pseudo-time trajectory results are presented in great technical detail but lack biological insights. The authors should streamline these sections, emphasizing developmental implications (e.g., Kranz anatomy formation, vascular patterning) rather than computational procedures.

REPLY:

[Page 15, lines 437-448]: We appreciate the constructive suggestion, and have revised this section to emphasize the biological implication, specifically regarding developmental and vascular pattering.

6. The resolution of the 10x spatial platform is relatively low and captures multiple cells in a spot, potentially mixing signals from neighboring tissues. This limitation should be acknowledged more explicitly, especially when drawing conclusions about isoform-level regulation in narrow structures such as veins.

REPLY:

[Page 25, lines 742-759]: Thanks for the great comment. We have added a section of "Limitations of the study" to the Discussion. We have also noted that the recent emerging of single-cell or subcellular levels of high-throughput imaging and sequencing technologies could eventually overcome these constraints in future studies.

<u>Minor:</u>

1. Terms such as "isoform," "transcript," and "gene model" are sometimes used interchangeably, which may confuse readers. Consistent definitions should be maintained.

REPLY:

[Supplementary Table 2]: Thanks for noting these inconsistencies. We have standardized the usage of those terms throughout the manuscript, and provided a list of the nomenclature in Supplementary Table 2.

2. Several enrichment and marker analyses are described as "significant" with adjusted p-values < 0.05 (lines 220 and 657), but the correction method (e.g., Benjamini–Hochberg, Bonferroni) is not always stated. Clarifying this would enhance transparency.

REPLY:

[Page 11, line 303 & Page 29, line 885]: Thanks for pointing out this oversight. We have now indicated that the Bonferroni correction method was used for these analyses.

Reference

1 Liu, Y. *et al.* Constraint of accessible chromatins maps regulatory loci involved in maize speciation and domestication. *Nature Communications* **16**, 2477 (2025). https://doi.org/10.1038/s41467-025-57932-1

2 Wu, C.-C. *et al.* Serial Spatial Transcriptomes Reveal Regulatory Transitions in Maize Leaf Development. *Plant Biotechnology Journal* (2026). https://doi.org/https://doi.org/10.1111/pbi.70515

3 Liu, W.-Y. *et al.* Maize ANT1 modulates vascular development, chloroplast development, photosynthesis, and plant growth. *Proceedings of the National Academy of Sciences* **117**, 21747-21756 (2020). https://doi.org/doi:10.1073/pnas.2012245117

4 Lin, J. J., Yu, C. P., Chang, Y. M., Chen, S. C. & Li, W. H. Maize and millet transcription factors annotated using comparative genomic and transcriptomic data. *BMC Genomics* **15**, 818 (2014). https://doi.org/10.1186/1471-2164-15-818

Response to Reviewers

We thank the Editor and the Reviewers for their time, constructive feedback, and positive evaluation of our manuscript [NPLANTS-250920790A] during this second round of review. We are thrilled that our work has been accepted in principle for publication as a Resource article in *Nature Plants*. In this final revision, we have carefully addressed all the reviewer's comments by revising the tone of in the results section to avoid overclaiming, and improved the readability of the histology images. In addition, we also addressed all remaining editorial formatting guidelines and refined our biological terminology to ensure maximum precision and clarity. Specifically, we have implemented the requested minor text adjustments, streamlined our subheadings to meet the length requirements, and carefully qualified our computational predictions and *in vitro* candidate targets as suggested. Below, we provide a point-by-point response detailing the specific modifications made to the text.

Reviewer #1:
Comments for the Author:

The authors have made a serious effort to address the previous concerns, and the new analyses improve the manuscript. I appreciate the added ATAC-seq integration, independent MAS-IsoSeq replicate, padlock-RCA in situ validation, and DAP-seq experiment. I have several remaining comments.

Reply:

We are deeply grateful to the reviewer for the positive evaluation of the newly added analyses. We have carefully revised the manuscript in accordance with your constructive comments. We believe these modifications have significantly improved the manuscript.

1. ATAC-seq integration

The ATAC-seq analysis is informative for linking alternative TSS-associated isoforms with chromatin accessibility. However, it should avoid describing as direct validation of novel isoforms or TSS usage. It is more appropriate as regulatory context.

Reply:

Thank you for the comment. We completely agree that ATAC-seq provides supportive epigenetic evidence rather than direct validation of novel isoform expression or TSS usage. Accordingly, we have revised the text to reframe these findings within the scope of providing "regulatory context."

[Page 8, lines 213-215]: We have removed the phrase "direct evidence" and replaced it with terminology denoting regulatory potential. The revised sentence is now read as:

"Peaks in the 'Individual' and 'Alternative TSS-only' categories indicate potential regulatory context for novel transcription initiation by confirming chromatin accessibility specifically at the alternative site."

[Page 22, lines 644-647]: To emphasize that the ATAC-seq data serves as regulatory context, we added the following sentence to the Discussion section: "Furthermore, we identified 9% of novel isoforms with alternative TSSs associated with proximal ACR (Extended Data Fig. 1d and Supplementary Table 13), providing valuable insight into the regulatory context for these novel isoforms."

2. Novel transcriptome validation

The poly-A motif screening, coding-potential prediction, PFAM scanning, and AlphaFold/stability analyses are useful, but they are computational annotations. In particular, there is no annotation of protein coding potential specifically of the new potential domains detected in novel isoforms. They do not directly demonstrate translation or protein function, so it should not be described as validation experiments.

Reply:

Thank you and we agree with your comment. These analyses are predictive and do not definitively demonstrate *in vivo* translation or protein function. To prevent overclaiming, we have systematically removed the term "validation" when referring to these computational analyses across the manuscript.

[Page 12, lines 346–347; Page 17, line 499; Page 22, line 673; Page 42, line 1316]: We have replaced "validation" with "assessment" in the above sentences (line 347, 498, and 672), and replaced "validate" with "examine" (line 1313), using more accurate terms to properly reflect the predictive nature of these computational annotations.

3. Padlock-RCA in situ hybridization

The padlock-RCA experiment is a valuable addition for selected candidates. Since this assay generally detects specific junctions or short isoform-specific regions, it should not be presented as confirming full-length novel isoforms. The probe design, target regions, controls, and signal quantification should be clearly described.

Reply:

We thank the reviewer for this crucial distinction. We understand that padlock probes specifically target precise splice junctions or short isoform-unique sequences, and cannot definitively verify full-length transcript integrity. We have revised this section to clarify that we are validating the presence of specific splice features rather than full-length transcripts.

[Page 13, lines 368-370]: The text has been modified to emphasize feature-specific detection: "We further validated the presence and spatial distribution of isoforms with novel splice junction or novel exon for the three target genes using padlock-probe RCA in situ hybridization".

[Page 45-46, lines 1430-1438]: We have included a detailed description for the probe design, target regions, and controls in the Methods section. Since the purpose of this experiment was to confirm the spatial presence and localization of the target features within tissues, signal quantification was not performed. The revised paragraph now reads: "Padlock probes were designed as 100 nucleotides (nt) oligos comprising three distinct functional domains: a symmetric 15-nt target-complementary arm at both the 5' and 3' termini, a 20-nt oligo detection binding site, and a 31-nt central backbone containing the primer-binding domain. The target-complementary arms were specifically designed to span the unique splice junction or the target exon exclusive to individual isoform transcript (Supplementary Table 24). To ensure the probe specificity, a BLASTN search was performed for each oligo sequence. Probes were only selected if the top 5 hits with an E-value $< 1 \times 10^{-5}$ matched exclusively to the target gene."

4. DAP-seq for LBD26

DAP-seq supports the DNA-binding capacity of LBD26 and identifies candidate binding sites. However, as an *in vitro* assay, it does not establish in vivo binding or functional regulation. Genes such as APC10 should therefore be described as candidate or putative targets unless additional supporting evidence is provided. The lack of mutant analysis remains a major limitation of this largely computational study.

Reply:

Thank you for your comment. We have revised the manuscript to ensure that genes with LBD26 binding sites were the candidate or putative regulating target, such as *APC10*. We understand the importance of mutant analysis to prove the functional regulation of transcription factors, including *LBD26*. Due to the time constraint, we were unable to include mutant analysis in this study. However, we have added a dedicated passage to our Discussion section to address this limitation.

[Page 25, lines 756-761] The newly added limitation now reads: "Lastly, while our DAP-seq data provides valuable in vitro evidence of the DNA-binding capacity of LBD26, a recognized limitation of this study is the lack of in vivo functional validation, such as mutant analysis. Future functional genetic characterization and stable mutant analyses will be essential to definitively map the in vivo regulatory networks governed by LBD26 during embryonic vein development."

[Page 20, lines 592, 608, & 610; Page 21, 616, & 619]: We have explicitly added terms like "candidate" or "putative" when referring to downstream target genes and their associated binding sites.

[Page 21, lines 623–625]: We have revised the statement regarding our results from providing "compelling evidence" to stating that they "highly suggest" a role for LBD26 in embryonic vein development. The revised sentence now reads: "Collectively, these results strongly suggest that LBD26 plays a role in embryonic vein development."

5. In the Fig. 4a–b, I think the current leaf histology image is still difficult to interpret. The tissue domains and boundary is hard to distinguish. In addition, several cross-sectional histology images in Extended Data Figs. 2, 3, and 7, and supplementary figures, are also blurry, making tissue boundaries difficult to assess. Higher-resolution images, clearer color contrast, or enlarged insets for key regions may improve readability.

Reply:

We thank the reviewer for the suggestions. We have revised the Visium image in Fig. 3a (formerly Fig. 4a) to include colored borders to help readers distinguish between different tissue domains, while also providing a parallel histology image that highlights and enlarges the embryonic vein tissue in the inset for the cross-sectional leaf tissue. Furthermore, we have improved the color contrast of the histology images to enhance readability, including main Fig. 3a (formerly Fig. 4a), Extended Data Figs. 3a (Visium images, formerly 2a), 4a–c (RCA and Visium images, formerly 3a–c), 8a–d (Visium images, formerly 7a–d), as well as Supplementary Figs. 3a–c (Visium images).

[Page 48, 1522-1523]: In order for the readers to access higher resolution histology images, we have deposited all the histology images with the highest resolution in Zenodo. This is also mentioned in the Data Availability section of this revised manuscript: "All the high-resolution histology images were uploaded to Zenodo repository (https://doi.org/10.5281/zenodo.20741009)."

Overall, the revision is stronger than the original submission, but these points should be addressed to improve precision and figure readability.

Reply:

We greatly appreciate your recognition and positive view of our study.

Reviewer #2:

Comments for the Author:

The authors provided an extensive revision of the manuscript, and had answered all my earlier questions.

Reply:

We sincerely thank the Reviewer for your great effort and positive assessment of our revisions. Your constructive feedback during the previous round heavily guided our improvements and substantially elevated the final quality of this manuscript. We deeply appreciate your valuable contributions as a reviewer.
